# Neoadjuvant, Adjuvant and Perioperative Treatment in Early-Stage Non-Small Cell Lung Cancer (NSCLC) with Actionable Genomic Alterations: Current Landscape and Future Perspectives

**DOI:** 10.3390/cancers18030493

**Published:** 2026-02-02

**Authors:** Prodromos Koutoukoglou, Giannis Mountzios

**Affiliations:** 1 First Oncology Department, Theageneion Anticancer Hospital, 54639 Thessaloniki, Greece; 2 Clinical Trials Unit, Fourth Department of Medical Oncology, Henry Dunant Hospital Center, 11526 Athens, Greece; gmountzios@gmail.com

**Keywords:** non-small-cell lung cancer (NSCLC), oncogene-driven, actionable, early, resectable, adjuvant, neoadjuvant, perioperative, EGFR, ALK

## Abstract

Surgical resection remains the cornerstone of curative-intent treatment for both oncogene and non-oncogene addicted early-stage non-small cell lung cancer, with neoadjuvant/adjuvant chemotherapy providing only a modest benefit in terms of disease-free survival and overall survival. Currently, in non-small-cell lung cancer without actionable molecular alterations, the addition of neoadjuvant or perioperative immunotherapy to chemotherapy has become the standard of care for resectable disease, with significant improvements in the rates of complete pathological response, major pathological response, and event-free survival. In terms of oncogene-addicted non-small-cell lung cancer, adjuvant treatment of completely resected disease with the third-generation EGFR inhibitor osimertinib in patients harboring activating *EGFR* mutations or the second-generation ALK inhibitor alectinib in patients with an *ALK* fusion/translocation has brought about a paradigm shift by significantly improving event-free survival and, in the case of osimertinib, overall survival. A glimpse into the future management of patients with early-stage disease and a common *EGFR* mutation or an *ALK* fusion/translocation may reveal a perioperative use of the respective targeted treatment. Data for the rest of the actionable molecular alterations remain limited, and those tumors are usually treated with algorithms used for non-oncogene-addicted non-small-cell lung cancer. This review aims to summarize all existing evidence about the current management of patients with early-stage non-small-cell lung cancer and actionable molecular alterations and to examine the future directions in this rapidly evolving field.

## Simple Summary

Non-small-cell lung cancer is a leading cause of morbidity and mortality worldwide. The landscape of early non-small-cell lung cancer treatment is currently undergoing a massive paradigm shift. Chemoimmunotherapy has revolutionized perioperative management of non-oncogene addicted early disease. Concerning oncogene-driven early non-small-cell lung cancer, adjuvant treatment with osimertinib in *EGFR*-mutant disease and alectinib in *ALK*-rearranged disease is considered a standard of care option for patients after complete resection. Ongoing studies—at different stages—evaluate targeted approaches in the perioperative setting of *EGFR*-mutant, *ALK*-rearranged, and other actionable molecular alterations. This comprehensive review aims to summarize the major breakthroughs in the perioperative management of oncogene-driven non-small-cell lung cancer and give an overview of promising ongoing efforts.

## 1. Introduction

Lung cancer represents a major global health challenge, ranking first in both incidence and mortality with an estimated 2.5 million new cases and over 1.8 million deaths in 2022 [1]. Non-small-cell lung cancer (NSCLC) accounts for up to 85% of the lung cancer burden and is in turn divided into two major subgroups: (1) non-squamous, encompassing the histological subtypes of adenocarcinoma, large-cell carcinoma, and others, and (2) squamous cell carcinoma [2,3]. The stage and extent of disease at diagnosis continue to be one of the most important prognostic factors of NSCLC. Despite advances in treatment with the advent of immunotherapy (IO) and targeted therapy, 5-year survival rates for advanced and metastatic NSCLC are lower compared to early-stage disease, which represents less than half of new diagnoses [4].

In the era of precision medicine and personalized treatment, performing a comprehensive molecular testing for the assessment of the programmed death-ligand 1 (PD-L1) status and for several oncogenic alterations is considered the standard of care (SoC), at least in the advanced and metastatic NSCLC and in the case of non-squamous histology [5,6,7]. This testing can preferably be performed on the tissue of the biopsy because of a lower rate of false negative results and more reliable assays [8,9]. However, in cases where tissue is not available, it can also be performed via a liquid biopsy in the blood; in fine needle aspiration (FNA) samples, e.g., from a lymph node; or in body fluids with a positive cytology, e.g., from a malignant pleural effusion [8,9].

The identification of oncogenic alterations in somatic testing has prognostic implications as the genes or proteins involved have, in most cases, a driver role and contribute to tumor aggressiveness, proliferation, and progression [10]. More importantly, they are significant as predictive biomarkers as the patients bearing them may have a favorable response in tailored treatment with targeted agents while being unsuitable candidates for other therapeutic tools like chemoimmunotherapy [11]. These “actionable” alterations encompass epidermal growth factor receptor (*EGFR*) common—and lately uncommon—mutations; anaplastic lymphoma kinase (*ALK*) rearrangements/translocations; proto-oncogene tyrosine-protein kinase ROS (*ROS1*) rearrangements/translocations; the v-Raf murine sarcoma viral oncogene homolog B (*BRAF*) V600E point mutation, rearranged during transfection (*RET*) rearrangements/translocations; human epidermal growth factor receptor 2 (*HER-2*) amplification or mutations; the Kirsten rat sarcoma virus (*KRAS*) G12C mutation; neurotrophic tyrosine receptor kinase (*NTRK*) 1/2/3 gene fusions/translocations; and mesenchymal–epithelial transition (*MET*) factor exon 14 (*METex14*) skipping mutations.

Targeted agents—mostly tyrosine kinase inhibitors (TKIs) but also newer molecules like antibody-drug conjugates (ADCs) or bispecific antibodies—that are specific for all the aforementioned alterations are approved by the Food and Drug Administration (FDA) and the European Medicines Agency (EMA) in the advanced-metastatic setting, either as first-line therapy or in previously treated patients, alone or in combination with other modalities like chemotherapy (ChT) [5,6,11]. Furthermore, concurrently with the endeavor to bring already approved agents to earlier lines or stages, there is also a plethora of new molecules that are currently investigated in late-phase clinical trials.

Regarding early-stage NSCLC, complete resection (R0) remains the cornerstone of curative-intent treatment for both oncogene and non-oncogene addicted cases [5,6]. Completeness of resection has been defined as the co-existence of four criteria (microscopically free margins, performance of a systematic lymph node dissection, absence of extracapsular node extension, and no tumor in the highest mediastinal node that was removed) by the International Association for the Study of Lung Cancer (IASLC) [12]. Incomplete or uncertain resection has been recognized as an adverse prognostic factor compared to complete resection [13]. For patients who are unwilling to undergo surgery or are medically inoperable due to age and comorbidities, curative radiotherapy (RT) by means of concurrent chemoradiotherapy (CRT), hypofractionated high-dose RT, or lately stereotactic body radiation therapy (SBRT), especially in peripheral Stage I lesions, emerges as an alternative solution to surgery [5,7,14,15,16,17].

Despite advances in surgical management, 19% of Stage I and 50% of Stage II operated patients experience disease recurrence within 5 years, while the 5-year recurrence-free survival (RFS) rate of Stage III patients with complete resection of their tumor is less than 40% [18,19]. Adjuvant cisplatin-based ChT, the SoC until several years, brought about a modest 5.4% absolute 5-year benefit in overall survival (OS) of Stage II–III completely resected disease, as demonstrated in the LACE meta-analysis of five trials [20]. A similar 5% 5-year OS improvement with neoadjuvant ChT in Stage I–III NSCLC patients in a 15-trial meta-analysis further consolidated the limited benefit of ChT in early-stage NSCLC [21].

The landscape of perioperative management of non-oncogene-addicted early NSCLC has witnessed a massive paradigm shift in recent years with the addition of IO into the ChT backbone, mostly in resectable disease. In terms of oncogene-addicted early NSCLC, adjuvant treatment of completely resected disease with the third-generation EGFR inhibitor osimertinib in patients harboring activating *EGFR* mutations or the second-generation ALK inhibitor alectinib in patients with an *ALK* fusion/translocation has rejuvenated the effort of enhancing patient outcomes and reducing disease recurrence. This narrative review aims to give an overview of the current situation in the perioperative management of early NSCLC with actionable oncogenic alterations, summarize all the experience in this field, and offer a glimpse into future perspectives.

## 2. The Rationale Behind the Neoadjuvant and Perioperative Approach in Early NSCLC and the Paradigm of Chemoimmunotherapy

Not many years ago, the necessity for effective strategies to lower the potential of cancer to relapse and increase the possibility of cure with surgery was more evident than ever before in the management of early and resectable NSCLC. The advent of IO and targeted agents has paved the way towards achieving these goals. One of the first dilemmas that naturally occurred was the optimal placement of the adjunct treatment methods in the therapeutic algorithm. Is the classic adjuvant strategy able and sufficient to move the field forward? Or will the administration of systemic therapy preoperatively prevail, following the paradigm of breast cancer and allowing an extra window for adjuvant treatment in a perioperative-sandwich approach?

The neoadjuvant approach has multiple advantages. Firstly, it is better tolerated compared to adjuvant therapy, and thus, more patients comply with and are able to receive the whole course of treatment, bearing in mind that patients with early and resectable NSCLC do not have an extensive disease burden and are generally asymptomatic or oligosymptomatic and thus have an acceptable performance status [20,21,22]. Furthermore, the earlier initiation of systemic therapy may eradicate micrometastatic disease, which is responsible for distant disease relapses [23,24,25,26,27,28]. The adequate control of micrometastatic disease can be effectively assessed with circulating tumor DNA (ctDNA) clearance [23,24]. Achieving a downstaging of tumor burden, leading to a less extensive surgery and an increase in the R0 rate, is also considered as one of the merits of the neoadjuvant approach [22,26,29].

Neoadjuvant treatment elicits pathologic responses besides its clinical and radiological impact. Major pathologic responses (≤10% residual cancer cells in the surgical specimen, MPR)—and pathologic complete response (pCR) to a greater extent—are closely associated with survival in various cancers, including NSCLC [30,31]. This correlation has enabled the utilization of pCR as a surrogate biomarker in lieu of survival endpoints like OS, event-free survival (EFS), or disease-free survival (DFS). Importantly, MPR and pCR are included as endpoints in all early NSCLC trials with a neoadjuvant therapy component, and notably, preoperative CIT has led to a striking increase in pCR compared to the rarity of its existence with ChT [25,27,32,33,34]. Moreover, the preoperative approach enables in vivo evaluation of treatment efficacy and may be used as a guide for tailored adjuvant treatment by means of (de)escalation. One extra argument for the neoadjuvant approach regarding IO and its mechanism of action is the presence of the whole intact tumor, which allows exposure of a more extensive neoantigen repertoire to the unrestrained primed immune system [35,36].

On the other hand, despite the short duration of neoadjuvant treatment (two to four cycles in most ChT and three to four cycles in all CIT trials), some patients with early NSCLC may experience disease progression during the prolonged interval from diagnosis to curative-intent surgery [37]. Apart from that, another subgroup of patients with resectable disease may experience significant adverse effects owing to IO or targeted therapy, leading to postponement of surgery, impediment to its execution due to technical difficulties, or significant perioperative morbidity. Some of these adverse effects are as follows: impaired wound healing with RET inhibitors pralsetinib and selpercatinib [38,39]; pneumonitis and interstitial lung disease owing to EGFR, ALK, and MET inhibitors [40,41]; cardiotoxicity with EGFR inhibitor osimertinib and BRAF/MEK inhibitors [42,43,44]; hepatotoxicity with KRAS-G12C inhibitor sotorasib [45]—especially if it is given sequentially after IO—and various autoimmune conditions affecting all systems, including the infamous and dreadful triple M syndrome (combination of myocarditis, myositis, and myasthenia gravis) secondary to immune checkpoint inhibitor (ICI) treatment [46].

Following the success of IO in the metastatic setting, its integration into the treatment of early-stage, non-oncogene-driven NSCLC has triggered a paradigm shift from upfront surgery to perioperative chemoimmunotherapy. The pioneering Phase 2 NADIM trial first demonstrated a numerically high pCR of 63.4% along with a 5-year progression-free survival (PFS) of 65% and a 5-year OS rate of 69.3% by adding anti-programmed death-1 (PD-1) inhibitor nivolumab to the three-cycle neoadjuvant ChT backbone with carboplatin and paclitaxel and continuing it postoperatively for 1 year in resectable Stage IIIA disease [47]. Soon after, a wave of Phase 3 approvals followed. Current standards of care include adjuvant atezolizumab or pembrolizumab following ChT based on Phase 3 trials IMpower 010 and KEYNOTE-091, respectively; purely neoadjuvant nivolumab with ChT according to Phase 3 trial CheckMate-816; and “sandwich” perioperative approaches utilizing nivolumab, pembrolizumab, or durvalumab based on Phase 3 trials CheckMate 77T, KEYNOTE-671, and AEGEAN, respectively [25,26,27,29,34,48,49]. While global approvals vary by PD-L1 expression levels and regional regulatory bodies, these regimens—including the China-approved toripalimab and tislelizumab based on Phase 3 trials NEOTORCH and RATIONALE-315—collectively represent the new standard of care for resectable non-oncogene-driven NSCLC [32,33]. The key characteristics of the major Phase 2 and 3 IO trials in resectable non-oncogene-driven NSCLC are included in Supplementary Table S1.

## 3. Resectable NSCLC with Actionable Genomic Alterations—Important Considerations

The frequency of targeted genomic alterations, which constitute the majority of NSCLC across all stages, varies across the globe. Namely, it is estimated to be around 60% in Western populations, while in Asian populations, the percentage may approach 80% [11]. According to retrospective real-world patient series, their prevalence appears to be statistically not different across stages except for *KRAS* mutations, which are more frequent in earlier stages [11,50,51]. Oncogene-driven NSCLC has a distinct clinical course compared to non-oncogene-driven disease. Moreover, oncogene-driven NSCLC does not constitute a single entity. Alterations like *EGFR* mutations or *ALK* translocations in NSCLC confer unique properties upon the corresponding molecular subtypes. This leads to distinguishing biological behaviors, including a differential response to various treatment modalities [10].

The existence of an *EGFR* mutation or *ALK* fusion translates into increased OS across all stages or only in the advanced stage, respectively, while the presence of a *KRAS* mutation is linked to a worse prognosis [10,52]. However, these data reflect the improvement in each target’s therapeutics, as *EGFR* mutations or *ALK* translocations were historically associated with increased disease aggressiveness, resistance to conventional ChT, and inferior patient outcomes until the advent of a grand repertoire of targeted agents against them [53]. Concerning early NSCLC, the existence of a frequent alteration like a common *EGFR* mutation or a rare one like *RET* fusion or *MET*ex14 skipping mutation leads to aberrant and mostly discordant post-operative outcomes (RFS, OS) compared to “wild-type NSCLC” in various retrospective patient series [54,55,56].

Both *EGFR*-mutant and *ALK*-positive NSCLC have a higher risk for brain metastases and osteolytic bone involvement, with a higher percentage of sclerotic bone lesions in patients with *ALK* translocations. *ALK*-positive disease has a predilection for lymphangitic carcinomatosis, extensive hilar lymphadenopathy, and involvement of the lower lung lobes compared to *EGFR*-mutant disease, which follows a more hierarchical metastatic progression along lymph node stations and predominantly involves the upper lung lobes [57,58]. While both subtypes are regarded as relatively chemoresistant, the efficacy of various chemotherapeutic drugs varies between them. Namely, pemetrexed leads to a higher response rate and PFS in the case of *ALK*-positive NSCLC compared to *EGFR*-mutant disease [59]. On the other hand, the presence of *EGFR* mutations may be linked to a higher sensitivity to radiation, leading to superior locoregional control of disease after RT, with or without concomitant ChT in early or locally advanced NSCLC [60]. However, this does not translate to a survival benefit, most likely due to a higher rate of out-of-field failure and distant metastases [61]. The presence of *ALK* translocations does not seem to influence patient outcomes after RT or CRT in locally advanced disease [62].

The similar prevalence of molecular alterations in early compared to advanced NSCLC, along with their influence on patient outcomes, incited the efforts for a tailored management of resectable oncogene-driven NSCLC besides advanced disease. One extra argument supporting this differential approach is the limited efficacy of IO—especially monoimmunotherapy—in these patients, excluding *KRAS* G12C mutation, *BRAF* non-V600E mutation, and *MET* amplification [63]. This could be explained by low PD-L1 expression, tumor mutational burden and an immune-suppressive tumor microenvironment (TME), features consistent with poor response to ICIs [64,65]. With respect to the perioperative CIT trials, patients with *EGFR* mutations or *ALK* translocations, which are the only oncogenes included in the compulsory molecular testing of early disease as per most guidelines, were underrepresented. Specifically, CheckMate-77T excluded patients with *EGFR* alterations while AEGEAN enrolled 51 patients with *EGFR* alterations, which were characterized by a trend toward improved EFS and pCR with perioperative CIT compared with ChT alone but were later excluded from the intention-to-treat analysis [25,34,66]. In KEYNOTE-671, 33 patients with confirmed *EGFR* mutations were included and had an improved EFS, with only 1/14 patients in the CIT arm experiencing recurrence compared to 10/19 in the ChT arm [27,29]. Meanwhile, an additional 66% of the study population had unknown *EGFR* status due to *EGFR* testing not being a prerequisite for enrolment [27,29]. Regarding patients with *ALK* fusions/translocations, these patients constituted only 5.2% of the KEYNOTE-671 population and were excluded from the intention-to-treat analysis of the AEGEAN trial due to the limited efficacy of both arms, despite the initial allowance of their enrolment [27,29,34,66].

Following the paradigm of non-oncogene-driven early disease, pCR and MPR have been heavily used as endpoints in all major trials of neoadjuvant or perioperative treatment of early NSCLC with actionable alterations. A 2025 systematic review and meta-analysis, which included 14 trials with ChT, ChT with RT or ICI, and ICI only, demonstrated a strong correlation between pCR and EFS and a moderate correlation between pCR and OS [67]. The moderate association of pCR with OS, the wide 95% CI (0.04–1.00) reflecting real-world heterogeneity, a more rigorous statistical interpretation, and the role of adjuvant treatment may temper the enthusiasm for the use of pCR and MPR as sole primary endpoints in major perioperative or adjuvant trials [68]. In the case of oncogene-addicted NSCLC, the relationship between pathological response and long-term outcomes is less strong. Specifically in *EGFR*-mutant disease, pCR and MPR rates with EGFR inhibitors are lower compared to ChT-ICI combinations in non-oncogene-driven disease or ALK inhibitors in *ALK*-positive disease, which is most likely attributed to their cytostatic rather than cytotoxic or immune-modulating mechanism of action [37,69]. Lower pathological response rates, along with the fact that patients who do not achieve MPR or pCR can still have excellent long-term survival by continuing highly effective adjuvant TKI treatment, limit the use of MPR or pCR as robust surrogate endpoints in early oncogene-addicted NSCLC [70,71].

Minimal residual disease (MRD) assessment via circulating tumor cells (CTCs), ctDNA, or other tumor-related material strongly correlates with survival outcomes and recurrence in NSCLC according to accumulated evidence and thus is regarded as an emerging prognostic indicator [72]. Following curative-intent therapy, non-detectable MRD has been linked to prolonged survival, while detectable—especially when persistently detectable—has been linked to an increased risk for disease relapse and death [73]. These correlations have been confirmed in several prospective studies, including Phase III trials of perioperative ChT-ICI or TKIs [74,75]. Important points for consideration and limitations include its low negative predictive value (a large portion of disease recurrence takes place in patients with non-detectable MRD after curative-intent treatment) compared to a high positive predictive value, the undetermined timepoint for post-operative MRD assessment, and the lack of an optimal threshold due to a multitude of options and assays [73]. Specifically for early NSCLC, another limitation lies in the fact that the low shedding of CTCs or ctDNA in early-stage disease requires assays with ultra-high sensitivity to avoid false negatives. Furthermore, different oncogenic alterations and anatomical sites (e.g., CNS) exhibit differential shedding patterns that may decouple the MRD status from actual tumor burden [73]. Thus, MRD assessment has not yet earned its place in the treatment algorithm of early NSCLC, regardless of the presence of actionable alterations.

Despite the limitations, a dynamic longitudinal monitoring of MRD with an approved method or assay preoperatively, postoperatively, and after adjuvant therapy could emerge as a useful strategy for treatment personalization after validation in prospective trials. Detectable CTCs or ctDNA in the preoperative period could be used as a decision tool for the administration of preoperative treatment, which should not follow a “one-size-fits-all” approach. Likewise, CTCs or ctDNA beyond a prespecified threshold in the preoperative period could be a signal for the intensification of preoperative treatment, e.g., with the addition of ChT or RT or with the prolongation of systemic therapy. Moreover, disease progression via MRD assessment could prompt surgery or definitive RT along with a change in the systemic treatment component. In the postoperative period, non-detectable MRD could contribute to the avoidance of overtreatment by enabling a safe treatment deintensification in terms of dosage and duration or the complete omission of adjuvant treatment and radiologic surveillance. On the other hand, detectable levels of CTCs or ctDNA could bring about treatment escalation by prolonging the duration, increasing the dose of the standard of care regimen, or introducing extra treatment modalities like ChT or RT [73].

Beyond molecular, pathological, and radiological responses and survival outcomes, neoadjuvant therapy may introduce unique surgical considerations like dense nodal fibrosis, inflammation, and adhesions induced by neoadjuvant chemoimmunotherapy, which increase the extent and complexity of the operation [76]. Neoadjuvant treatment with an EGFR inhibitor led to no delays in planned surgery. Only one patient receiving afatinib passed away in the postoperative period due to respiratory failure, with the necropsy analysis indicating tumor replacement by fibrotic scar tissue and lymphocyte infiltration [77]. Intra- and postoperative complications occurred in less than 15% in all neoadjuvant EGFR TKI trials, were up to Grade 3 and reversible in most cases, could not be attributed solely to neoadjuvant treatment, and included pulmonary embolism, pneumothorax, chylothorax, respiratory failure, prolonged air leakage, bronchopleural fistula, and subcutaneous emphysema, among others [77,78,79,80,81,82,83,84]. R1/R2 resection was mostly an issue with first- and second-generation inhibitors (12–31%), while the corresponding rates for osimertinib and aumolertinib were 5.3% (2 studies) and 0%, respectively [77,78,79,81,82,83,84]. Conversion to pneumonectomy was a rare phenomenon, taking place only in 2/27 patients (7.4%) receiving osimertinib [83].

Neoadjuvant treatment with ALK inhibitors leads to a potentially steeper surgical pathway, most likely due to more extensive hilar/mediastinal lymphadenopathy and treatment-induced changes. Up to 10% of patients experienced a delay in surgery due to treatment-related adverse events (AEs). However, no deaths or significant intra- or postoperative complications were reported. R0 rates were higher compared to neoadjuvant EGFR TKI, ranging from 86% to 100% [85,86,87,88,89]. In total, 3/29 (10.3%) patients receiving alectinib or crizotinib and 1/11 (9%) patient, who received lorlatinib, had to convert from a minimally invasive lobectomy to open thoracotomy [85,86]. All patients in the lorlatinib study faced surgical difficulties due to adhesions and fibrosis, ranging from mild (3/11, 27.2%) to moderate–severe difficulties (8/11, 72.8%). However, the lorlatinib study enrolled only patients with locally advanced Stage III NSCLC [85]. Taking all this into account, it is imperative that thoracic surgeons are involved in a multidisciplinary decision-making approach for the optimal management of early oncogene-driven NSCLC.

## 4. EGFR

The prevalence of *EGFR* alterations and mutations in NSCLC varies significantly according to ethnicity. In Western populations, it is estimated to be around 15–20%, while it can surpass 50% in Asian populations [11]. The presence of an *EGFR* mutation is identified more frequently among female, non-smoker, or adenocarcinoma patients [90]. Moreover, there are various classifications of the different *EGFR* mutations. Classical or common mutations include deletions in exon 19 (Ex19del) and exon 21 codon p.Leu858Arg (L858R) point mutations, which comprise 85–90% of the entirety of this group and are sensitive to first-, second-, or third-generation inhibitors, with the latter being able to overcome the resistant T790M point mutation [91]. On the other hand, atypical mutations constitute 10–15% of all *EGFR* mutations; include exon 20 insertions, G719X, S768I, and L861Q, among others; express reduced and variable sensitivity to EGFR inhibitors; and need specific management like the second-generation afatinib for G719X, S768I, and L861Q or the bispecific antibody amivantamab for exon 20 insertions [91]. A more contemporary classification distinguishes *EGFR* mutations on the basis of structure differences and drug sensitivity into four groups: (i) “classical-like” (distant from the ATP-binding pocket), (ii) “T790M-like” in the hydrophobic core, (iii) “Ex20ins-L” (insertions in the loop at the C-terminal end of the αC helix in exon 20), and (iv) “PACC”, which are mutations recognized as P-loop and αC-helix compressing [92]. Concerning influence on prognosis, there is a discrepancy in data regarding the role of *EGFR* mutations in completely resected NSCLC. Several studies report a protective role in *EGFR* mutations; others report a higher risk of metastatic recurrence, including intracranial progression, while more recent patient series report no association between *EGFR* mutations and DFS or OS in the era before adjuvant osimertinib [93,94,95,96,97].

### 4.1. Adjuvant Approaches

The first evidence of the protective role of EGFR inhibitors in the adjuvant setting of completely resected early *EGFR* mutant NSCLC was observed in two retrospective patient series from the early 2010s, where the use of first-generation agents erlotinib or gefitinib resulted in significantly improved DFS or 2-year DFS with a trend toward improved OS or 2-year OS compared to placebo [98,99]. Another retrospective study, which compared adjuvant gefitinib (median duration of treatment 20.7 months) with four cycles of two-drug platinum-based adjuvant ChT in completely resected Stage II–IIIA (7th TNM Edition) NSCLC with common *EGFR* mutations, conferred a statistically significant benefit with the EGFR inhibitor in median DFS, with 34.9 versus 19.3 months (HR: 0.36, 95% CI 0.19–0.68, *p* = 0.001) and less frequent Grade 3 or higher AEs [100]. With respect to randomized studies on adjuvant administration of EGFR inhibitors, the first two Phase 3 trials investigated the use of first-generation agents for two years versus placebo on all-comers with completely resected Stage IB–IIIA disease according to the 6th TNM classification and did not focus on *EGFR* mutation carriers. Namely, in the prematurely terminated North American NCIC CTG BR19 (NCT00049543), with only 4% of patients harboring an *EGFR* mutation, there was no improvement with gefitinib in OS (HR: 1.24, 95% CI 0.94–1.64, *p* = 0.14) or DFS (HR: 1.22, 95% CI 0.93–1.61, *p* = 0.15) in the whole study population, with similar observations in the 15 *EGFR* mutant patients (OS → HR: 3.16, 95% CI 0.61–16.45, *p* = 0.15; DFS → HR: 1.84, 95% CI 0.44–7.73, *p* = 0.395) [101]. Similarly, only 161 of the 973 enrolled patients (16.5%) in the international RADIANT trial (NCT00373425) harbored a mutation or amplification in *EGFR*. This led to a non-significant difference in median DFS in the unselected population with erlotinib compared to placebo (50.5 vs. 48.2 months, HR: 0.90, 95% CI 0.74–1.10, *p* = 0.324), while in the *EGFR*+ group, a clinically meaningful but not statistically significant difference in median DFS with erlotinib (46.4 vs. 28.5 months) was observed because of hierarchical testing procedure (HR: 0.61, 95% CI 0.38–0.98, *p* = 0.039) [102]. OS was not statistically significant in the *EGFR*+ group (HR: 1.09, 955 CI 0.54–2.16) [102]. Adjuvant ChT was allowed in both trials, with the respective rates being 17% and 53% [101,102].

The next step was to evaluate first-generation EGFR inhibitors in a population with classical *EGFR* mutations. Adjuvant erlotinib for 2 years in IA-IIIA patients (7th TNM Edition) yielded a 5-year DFS of 56% and 5-year OS of 86% in the North American single-arm Phase 2 SELECT study (NCT00567359), which were improved compared with a historic matched population [103]. Four Chinese randomized, open-label Phase 2 trials further assessed first-generation agents. In GASTO1003-CORIN (NCT02264210), post-operative icotinib in Stage IB patients without prior ChT led to a significantly improved 5-year DFS compared to another observation (88.5% vs. 67.7%, respectively; HR: 0.38, 95% CI 0.18–0.83, *p* = 0.012), with a marginal improvement in 5-year OS due to the small sample size and 83% crossover rate (98.3% vs. 90.5%, respectively; HR: 0.15, 95% CI 0.02–1.27, *p* = 0.045) [104]. Icotinib for a duration of 4 to 8 months after four cycles of platinum-based ChT failed to improve 12-month (100% vs. 88.9%, *p* = 0.122), 18-month (95.2% vs. 83.3%, *p* = 0.225), and 24-month DFS (90.5% vs. 66.7%, *p* = 0.066) vs. another observation in 41 patients with IB (with high-risk features) to IIIA in the NCT02430974 study [105]. Meanwhile, 2 years of adjuvant erlotinib compared to four cycles of cisplatin/vinorelbine in completely resected Stage IIIA disease improved the median DFS (HR: 0.38, 95% CI 0.20–0.70, *p* < 0.001) and median OS (84.2 vs. 61.1 months, respectively; HR: 0.37, 95% CI 0.19–0.73, *p* = 0.003) in the EVAN trial (NCT01683175) [106]. The EVAN study, which included mostly N2 patients (94.1% in the erlotinib and 100% in the ChT arm), was the first to demonstrate a clinically meaningful improvement in OS with targeted treatment compared to ChT in completely resected Stage III *EGFR* mutant NSCLC. Finally, in another sequential approach, 6 months of gefitinib after four cycles of adjuvant ChT with carboplatin/pemetrexed in 60 patients with an R0 resection of Stage IIIA-N2 NSCLC harboring common *EGFR* mutations yielded an improvement compared to observation in 2-year DFS (78.9% vs. 54.2%; HR: 0.37, 95% CI 0.16–0.85, *p* = 0.014) but not in 2-year OS (92.4% vs. 77.4%; HR: 0.37, 95% CI 0.12–1.11, *p* = 0.076) [107].

Subsequently, three Phase 3 randomized studies evaluated 2 years of a first-generation EGFR inhibitor vs. four cycles of platinum-based ChT after a complete resection of Stage II–IIIA (N1–N2) NSCLC with *EGFR* exon 19 or 21 mutations with DFS as the primary end point [108,109,110]. In the Chinese ADJUVANT/CTONG1104 trial (NCT0140579), gefitinib significantly prolonged the median DFS (28.7 vs. 18 months; HR: 0.60, 95% CI 0.42–0.87, *p* = 0.0054) without an improvement in the median OS (75.5 vs. 62.8 months; HR: 0.92, 95% CI 0.62–1.36, *p* = 0.674), which was attributed mostly to subsequent therapy, while achieving a favorable safety profile [108]. Gefitinib failed to corroborate its activity by not prolonging median DFS compared to ChT (35.9 vs. 21 months; HR: 0.92, 95% CI 0.67–1.28, *p* = 0.63) and 5-year OS (78.0% vs. 74.6%; HR: 1.03, 95% CI 0.65–1.65, *p* = 0.89) in the Japanese IMPACT/WJOG6410L study with a crossover rate of 52% [109]. However, in the Chinese EVIDENCE trial (NCT02448797), icotinib led to a median DFS of 47 months compared to 22.1 months in the ChT arm (HR: 0.36, 95% CI 0.24–0.55, *p* < 0.0001), with immature OS data at the time of the publication and only 1% serious treatment-related adverse events [110]. Two different duration regimens for icotinib after ChT were compared to a placebo in the same population as the aforementioned studies in the early-terminated Phase 3 randomized GASTO1002/ICTAN trial (NCT01996098) with recently published results [111]. Both 6 and 12 months of adjuvant icotinib led to significant prolongation of DFS (HR: 0.41, 95% CI 0.27–0.62, *p*  <  0.001 and HR: 0.40, 95% CI 0.27–0.61, *p*  <  0.001, respectively) and OS (HR: 0.56, 95% CI 0.32–0.98, *p*  =  0.038 and HR: 0.55, 95% CI 0.32–0.96, *p*  =  0.032, respectively) compared to placebo while 6 additional months of icotinib does not provide extra benefit and thus is not recommended [111].

Before GASTO1002/ICTAN, improvements in DFS with a first-generation EGFR TKI did not translate into an OS benefit, which could be attributed to subsequent treatment with an EGFR TKI post-progression. Furthermore, DFS curves converged after cessation of adjuvant treatment, possibly due to a lack of protective action in the central nervous system (CNS), as demonstrated by the increased rate of brain relapse in all Phase 3 trials before GASTO1002/ICTAN [108,109,110]. The main difference between GASTO1002/ICTAN and the rest of the Phase III first-generation EGFR TKI trials was that it tested a combined sequential approach post-ChT instead of evaluating targeted therapy versus ChT. The mechanism of action of targeted treatment with TKIs is mainly cytostatic through applying selective pressure to cancer cells, which is evident during the resurgence of disease or flare after treatment interruption with an EGFR TKI in the metastatic setting. On the other hand, ChT’s benefit is primarily driven by cytotoxicity [112]. Thus, sequential use of ChT and EGFR inhibition and exploiting the merits of both modalities, as well as their synergistic effect, as demonstrated in in vitro studies, could be a rational strategy as adjuvant treatment for high-risk resected *EGFR* mutant NSCLC [113]. However, the use of first-generation EGFR inhibitors precludes inhibition of the T790M mutation, which is responsible for up to 60% of acquired resistance and treatment failure, while it also demonstrates inferior intracranial activity compared to third-generation inhibitors like osimertinib [69,114,115].

Following improvement in efficacy and safety with osimertinib compared to first-generation EGFR inhibitors in advanced metastatic disease, owing to its aforementioned qualities of effectively inhibiting T790M mutation and penetrating–exerting its effect in the CNS, it was also assessed in the adjuvant setting [116,117]. In the groundbreaking Phase 3 ADAURA trial, following complete resection of Stage IB–IIIA NSCLC (7th TNM Edition) with common *EGFR* mutations and optional post-operative ChT, 682 patients were randomized across 3 years with osimertinib or placebo, with investigator-assessed DFS among patients with Stage II–IIIA disease as the primary end point and secondary endpoints including DFS among patients with Stage IB–IIIA disease, OS, and safety. At 44.2 months, the median DFS for Stage II–IIIA disease for osimertinib was 65.8 months vs. 21.9 months with placebo (HR: 0.23, 95% CI 0.18 to 0.30), while a 4-year DFS rate was 70% with osimertinib vs. 29% with placebo [70]. The median DFS in the overall population was also improved with osimertinib vs. placebo (65.8 months vs. 28.1 months, HR: 0.27, 95% CI 0.21–0.34) without an overwhelming impact on quality of life, as observed with first- and second-generation inhibitors. The beneficial effect of osimertinib on DFS increased with stage (IB → HR: 0.41, 95% CI 0.23–0.69; II → HR: 0.34, 95% CI 0.23–0.52; and IIIA → HR: 0.20, 95% CI 0.14–0.29) [70].

ADAURA was the first Phase 3 trial with adjuvant EGFR TKI inhibition that led to CNS protection and improvement in OS and remained the only one until the recently published results of GASTO1002/ICTAN. Namely, CNS DFS HR with osimertinib compared to placebo was 0.24 (95% CI 0.14–0.42) in Stage II–IIIA disease and 0.36 (95% CI 0.23–0.57) in the overall population, while the estimated probability of a CNS relapse at the timepoint of 3 years was 2% with osimertinib vs. 13% with placebo [118]. Concerning OS, the 5-year OS in Stage II–IIIA patients was 85% with osimertinib compared to 73% with placebo (HR: 0.49, 95% CI 0.33–0.73, *p* < 0.001), while in the overall population, the corresponding rates were 88% and 78%, respectively (HR: 0.49, 95% CI 0.34–0.70, *p* < 0.001). Similarly with DFS, the benefit increased proportionately with respect to the stage (IB → HR: 0.44, 95% CI 0.17–1.02; II → HR: 0.63, 95% CI 0.34–1.12; and IIIA → HR: 0.37, 95% CI 0.20–0.64) [118]. Based on findings from the ADAURA trial, adjuvant osimertinib for 3 years after complete resection of Stage IB–IIIA NSCLC with common *EGFR* mutations is currently approved in the US, EU, China, and Japan. Interestingly, osimertinib’s benefit is independent of ChT administration (ChT → DFS HR: 0.29, 95% CI: 0.21–0.39; OS HR: 0.49, 95% CI: 0.30–0.79 and no ChT → DFS HR: 0.36, 95% CI: 0.24–0.55; OS HR: 0.47, 95% CI: 0.25–0.83) [112]. Furthermore, the trial was unblinded after superiority in terms of DFS was ascertained, which could affect OS estimates. The OS could also be influenced by the fact that only 43% of the patients (75/205) in the control arm received osimertinib as subsequent treatment, despite 85% of participants in the control arm (174/205) receiving anticancer treatment after confirmation of progression [112].

There are many challenges and unanswered questions regarding the optimal adjuvant treatment of patients with completely resected NSCLC with *EGFR* mutations. The 5-year DFS rate of *EGFR*-positive NSCLC patients from Stage IB to IIIA, who underwent complete surgical excision and did not receive a TKI post-operatively, reached 37.2%, suggesting that more than one-third of candidates for adjuvant targeted therapy could be cured without adjuvant osimertinib or icotinib [94]. By estimating this “favorable” subgroup, we could avoid overtreatment, increased healthcare burden, and the negative impact on patients’ lives from unwanted—and useless—toxicity. MRD is strongly associated with recurrence and could be utilized to identify those who would benefit most from post-operative treatment, as indicated by retrospective and prospective studies like the Phase 2 CELEBRATE-NSCLC [74,119,120,121]. MRD negativity at baseline and conversion from preoperative ctDNA positivity to postoperative MRD negativity led to improved outcomes, including DFS, compared to baseline MRD positivity and non-conversion, respectively, in multiple studies [122,123]. In an exploratory analysis of the ADAURA trial, MRD preceded most radiographic DFS events in both arms during adjuvant treatment and follow-up period by a median of 4.7 months (95% CI 2.2–5.6), similarly to previous retrospective and prospective studies [74,124,125]. Furthermore, the status of negativity in MRD and the absence of a DFS event were maintained for most patients in the osimertinib arm during treatment and follow-up after its cessation compared to placebo (86% vs. 36% at 36 months, HR: 0.23, 95% CI 0.15–0.36), and the majority of MRD or DFS events took place after and within one year of osimertinib discontinuation or completion (19/28, 68% and 11/19, 58%, respectively) [124]. Future studies could include MRD detection as a decision-making tool for personalization of adjuvant treatment after identification of the optimal technique for it, taking into account that, due to lower disease burden in early NSCLC, the cut-off limit is low, and increased sensitivity is needed.

The presence of co-mutations could further expand the personalization of treatment. For instance, *TP53* mutations in advanced *EGFR* mutant NSCLC are linked to resistance to EGFR inhibition and a higher mutation burden, while the concomitant existence of mutations in *TP53* and *RB1* is a risk factor for SCLC transformation [126,127,128]. Ongoing clinical trials explore the feasibility of treatment escalation strategies in patients with these co-mutations [129,130]. In early-stage *EGFR* mutant NSCLC, the *TP53* mutation is observed in up to 70% of patients and is also an adverse prognostic factor [131]. In a retrospective subgroup genomic analysis from the ADJUVANT trial, the identification of five predictive biomarkers, namely *TP53* mutations, *RB1* alterations, and amplification of *NKX2-1*, *CDK4*, and *MYC*, led to three prognostic subgroups based on efficacy of different treatment modalities (highly TKI-preferable, TKI-preferable, and ChT-preferable) [132]. Similar strategies could be implemented in the future following validation in prospective trials. Regarding ChT with stage as the only criterion, 5-year OS was improved in patients who received it compared to those who did not in Stages II–IIIA (87% vs. 80% in the osimertinib arm and 75% vs. 66% in the placebo group), suggesting that it can be omitted in Stage IB [118].

Patients with tumors less than 4 cm or with uncommon mutations were not represented in the ADAURA trial or the already completed aforementioned studies. Thus, caution should be exercised when treating patients with uncommon *EGFR* mutations or smaller tumors. Extrapolation of accumulated evidence from EGFR inhibitors in the adjuvant treatment of patients with >4 cm tumors with common *EGFR* mutations should not be encouraged without prospective validation. The prospective Phase 2 two-arm APPOINT study (NCT04922138) enrolled completely resected Stage IA patients with common and uncommon *EGFR* mutations and high-risk factors (≥10% of solid, micropapillary, and/or complex gland components) and evaluated the adjuvant treatment with 3 years of third-generation aumolertinib vs. the placebo with a 2-year DFS rate as primary endpoints, and 3-, 4-, and 5-year DFS rates and a 5-year OS rate were evaluated as secondary endpoints [133]. At a median follow-up time of 20 months, no patient in the aumolertinib arm and 4 out of 51 patients in the placebo arm experienced disease recurrence (2-year DFS → 100% vs. 75%; HR: 0.116, 95% CI 0.016–0.838, *p* = 0.033), with no ≥Grade 3 AEs or unexpected toxicity in the experimental arm [134]. The ongoing randomized Phase 3 ADAURA2 (NCT05120349) will address 3 years of adjuvant osimertinib vs. the placebo after R0 resection in patients with Stage IA2–IA3 (8th TNM Edition) non-squamous NSCLC and common *EGFR* mutations; will enroll Chinese, non-Chinese Asian, and non-Asian patients; and will stratify them according to race, *EGFR* mutation, and risk (high-risk when ≥1 of the following risk factors is present: largest diameter of invasive component of primary tumor >2 cm, lymphovascular invasion, and/or high-grade histology with ≥20% micropapillary, solid, or complex gland adenocarcinoma) [135]. The primary endpoint will be DFS in the high-risk subgroup, while secondary endpoints include DFS in the overall population, OS, CNS DFS, and safety [136]. Furthermore, a sub-cohort of the Phase 3 PACIFIC-4 trial (NCT03833154) will evaluate the efficacy of 3 years of osimertinib as consolidation treatment in medically inoperable Stage I/II EGFR mutant NSCLC after curative SBRT, with 4-year PFS as the primary endpoint and only common mutations allowed [137].

The maximum duration of adjuvant treatment with osimertinib was three years, while first-generation inhibitors were administered for up to two years in Phase 3 trials, with the exception of six months of icotinib in the GASTO1002/ICTAN trial [70,108,109,110,111]. Kaplan–Meier curves for DFS began to converge after discontinuation of treatment, following the same pattern with both intracranial and extracranial DFS events [70,108,109,110,118]. This feature fits the mechanism of action of EGFR TKIs, which cannot completely eradicate micrometastatic disease and quiescent cancer cells and thus can only delay disease relapse [112]. One possible course of action could be to prolong the duration of adjuvant treatment or increase the dose, or even continue until progression or unacceptable toxicity, following the paradigm of other solid tumors like gastrointestinal stromal tumors. Patients with a higher Stage (IIIA) and thus a higher recurrence rate could derive the greatest benefit from this approach. Extended by a definite amount of time, adjuvant treatment could also potentially prevent progression to metastatic disease and the personal, psychological, and financial cost of lifelong treatment for a subgroup of patients. However, balance with toxicity has to be maintained as only 66% in the ADAURA trial succeeded in completing 3 years of treatment with osimertinib [118]. Prolonged treatment could potentially lead to the development of drug resistance mutations like the C797S [138]. Another argument against prolonged treatment—and adjuvant TKI treatment by extension—is the contingency of overtreatment in patients already cured by surgery, a scenario that occurs most frequently in Stage IB patients. The appropriate use of MRD monitoring after prospective validation could emerge as an invaluable tool to help answer who to treat and for how long.

The respective rates of treatment completion for six months of icotinib, twelve months of icotinib, two years of icotinib, and two years of gefitinib in the IMPACT and the CTONG1104 trial were 78.5%, 77.3%, 45.6%, 60.3%, and 61.2%, respectively [108,109,110,111,118]. Specifically, for icotinib, the results are conflicting. Despite no extra benefit with twelve vs. six months in the GASTO1002/ICTAN trial, two years of adjuvant icotinib yielded a significant improvement in both median DFS (HR: 0.51, 95% CI 0.28–0.94, *p* = 0.029) and median OS (HR: 0.34, 95% CI 0.13–0.95, *p* = 0.0317) compared to one year in Stage II–IIIA disease in the exploratory, randomized, open-label Phase 2 ICOMPARE study (NCT01929200) [139]. The ongoing single-arm Phase 2 TARGET study (NCT05526755) will explore adjuvant treatment with five years of osimertinib in a resected Stage II–IIIB population [140]. All *EGFR* mutations will be included, and the option of adjuvant ChT will be allowed. The primary endpoint is investigator-assessed 5-year DFS in the common mutation cohort, and secondary endpoints include 3-, 4-, and 5-year DFS and OS in both the common and uncommon mutation cohorts, safety, and CNS DFS [140].

One unmet need concerning the adjuvant treatment of EGFR-mutant NSCLC is unraveling the mechanisms of resistance in third-generation inhibitors in early disease and either preventing or tackling it, following the footsteps of advanced disease management, with new targeted molecules like the EGFR-MET bispecific antibody amivantamab and combination approaches like amivantamab plus third-generation irreversible EGFR inhibitor lazertinib or osimertinib plus ChT in first-line (prevention) treatment or amivantamab plus ChT with or without lazertinib post-osimertinib (tackling) [141,142,143]. The optimal strategy at disease progression after post-operative osimertinib, whether it is a rechallenge or the offer of a subsequent monotherapy or treatment combination, has yet to be defined. Furthermore, in the era of potent TKIs, adjuvant ChT could be made redundant following the paradigm of the Phase 3 ALINA trial and taking into account the distinct clinical history and biological course of *EGFR*-mutant and *ALK*-positive NSCLC. In ALINA, which will be discussed in the section devoted to adjuvant approaches for *ALK*-positive NSCLC, 2 years of adjuvant treatment with the ALK inhibitor alectinib after complete resection of Stage IB–IIIA *ALK*-positive NSCLC outperformed platinum-based ChT in terms of PFS, with OS data still being immature [144]. The omission of ChT could concern only patients with a lower risk for recurrence, e.g., Stage IB, a larger subgroup, or even the whole cohort of patients with early, completely resected *EGFR*-mutant NSCLC. Emerging screening techniques, like MRD kinetics, could guide pertinent decision-making. Lastly, the key characteristics of ongoing adjuvant trials in early *EGFR*-mutant NSCLC are summarized in Table 1.

### 4.2. Neoadjuvant and Perioperative Approaches

The timeline of neoadjuvant administration of EGFR inhibitors followed the same hierarchical process as the evolution of adjuvant treatment. The first agents that were evaluated were first-generation inhibitors. The feasibility and efficacy of neoadjuvant gefitinib in *EGFR*-mutant early NSCLC were observed for the first time in a single-arm Phase 2 study (NCT00188617) in 36 unselected patients with Stage I disease (6th TNM Edition) [78]. Up to 28 days of gefitinib led to an objective response rate (ORR) of 11% (4/36 with partial response), and 3/4 responding patients received adjuvant gefitinib for up to two years. The only significant predicting factor of objective response was the presence of an *EGFR* mutation. Grade 3 toxicity was observed in three patients without a need for dose reduction or discontinuation. Despite 3 patients (8%) having progressive disease (PD), all 36 of them proceeded to curative surgery. At a median follow-up of 2.1 years, 28 patients (78%) remained disease-free, including all four responders [78]. In a retrospective, single-center study, 10 patients with borderline resectable NSCLC with common *EGFR* mutations (all were either T2-T3N2M0 or T4N0M0 according to the 6th TNM Edition) received neoadjuvant gefitinib for three to five months followed by “salvage surgery” and at least six months of adjuvant gefitinib along with other modalities (4/10 patients received adjuvant ChT and 1 patient underwent post-operative radiation therapy) [77]. Clinical downstaging took place in every patient, while pathological downstaging happened in 9/10 patients. All of them underwent complete surgical excision, with pneumonectomy taking place in only one patient, who passed away on post-operative day 7 due to respiratory failure. A median PFS of 14 months and median OS of ≥36 months showed that neoadjuvant EGFR TKI treatment was a feasible and effective option [77]. Neoadjuvant gefitinib was also administered for 42 days in 33 patients with Stage II–IIIA NSCLC (7th TNM Edition) with *EGFR* exon 19 deletion or exon 21 L858R point mutation in the Chinese single-arm, Phase 2 ECTOP-1001 (NCT01833572) [79]. All patients received adjuvant ChT. The primary endpoint, ORR, was 54.5% (18/33, all of them with partial response), the MPR rate was 24.2% (12.1% pCR), the median DFS was 33.5 months, and the median OS was not reached at data cut-off. A prolonged DFS with no difference in OS was observed in patients with MPR compared to those with no MPR (*p* = 0.015 and 0.134, respectively) [79]. These promising efficacy results coexisted with a heavy toll in terms of toxicity. Moreover, 68.6% of patients experienced skin toxicity, and 48.6% experienced gastrointestinal toxicity, raising concerns about tolerability [79].

Another first-generation EGFR inhibitor tested in the neoadjuvant setting was erlotinib. In the prospective, single-arm, Chinese NCT01217619 trial focusing on Stage IIIA disease (7th TNM Edition), 56 days of neoadjuvant erlotinib in NSCLC with common *EGFR* mutations was compared to 2 weeks of platinum-based ChT in *EGFR*-wild disease [145]. In total, 80% and 50% of patients proceeded to surgery, respectively. Concerning patients where surgical excision was undertaken, the TKI cohort had a higher ORR (66.7% vs. 18.7%, all PR, *p* = 0.018), MPR (66.7% vs. 37.5%), and OS (51 vs. 20.9 months, *p* = 0.12) compared to the ChT cohort [145]. A perioperative approach was utilized in the randomized, Chinese Phase 2 EMERGING-CTONG 1103 trial (NCT01407822) in patients with Stage IIIA-N2 NSCLC with common *EGFR* mutations only [146]. Erlotinib, administered for 6 weeks preoperatively and up to 1 year postoperatively, was compared to 2 weeks of cisplatin-gemcitabine both pre- and postoperatively. The ORR was not significantly improved with erlotinib (54.1% vs. 34.3%; OR: 2.26, 95% CI 0.87–5.84, *p* = 0.092) compared to ChT. MPR was 9.7% and 0%, respectively, with no pCR in either of the arms [146]. The median PFS was significantly longer with erlotinib (21.5 vs. 11.4 months; HR: 0.39, 95% CI 0.23–0.67, *p* < 0.001) [146]. At 62.5 months, the median OS was 42.2 months with erlotinib and 36.9 months with ChT (HR: 0.83, 95% CI 0.47–1.47, *p* = 0.513), with 5-year OS rates being 40.8% and 27.6%, respectively (*p* = 0.252) [147]. 13.5% of patients had Grade 3–4 AEs with erlotinib postoperatively, while the corresponding rate for postoperative ChT was 29.4%. At the time of relapse, 71.9% of patients in the erlotinib arm received subsequent treatment, while the corresponding rate in the ChT arm was 81.8%. Subsequent targeted therapy, which had the greatest contribution to OS (HR: 0.35, 95% CI 0.18–0.70), was given to 46.9% and 69.7% of the two arms, respectively, either alone or in combination with ChT or other modalities [147].

Following the modest results with gefitinib and erlotinib, the activity of second-generation afatinib in the neoadjuvant-perioperative setting was investigated. The Chinese single-arm Phase 2 TEAM-LungMate004 (NCT04201756) assessed neoadjuvant afatinib for 8–16 weeks (2–4 cycles) and postoperative administration for up to 1 year in 33 Stage III resectable and potentially resectable patients with *EGFR* mutations excluding T790M point mutation and exon 20 insertions [148]. The ORR with neoadjuvant afatinib was 70.2% (all were partial responders), while MPR, pCR, and pathological downstaging rates were 9.1% (three patients), 3.0% (one patient), and 57.6%, respectively. Remarkably, more than two cycles of preoperative afatinib were linked to a higher ORR and rate of pathologic tumor regression compared to just two (94% vs. 69% and 59% vs. 41%, respectively) [148]. Toxicity was an issue, with 6.4% (three patients) experiencing treatment-related Grade 3/4 AEs, including diarrhea, interstitial pneumonia, and hepatotoxicity, one patient discontinuing due to Grade 4 diarrhea, and most patients experiencing diarrhea (78.7%), rash (78.7%), and stomatitis (68.1%) [148]. The perioperative approach was explored in the US single-arm Phase 2 ASCENT trial (NCT01553942) with afatinib induction for two months followed by concurrent chemoradiotherapy, surgical excision of resectable disease, and finally, consolidation afatinib for up to two years in both resectable and unresectable Stage III NSCLC with *EGFR* alterations (common mutations and one patient with an exon 18 deletion delE709_T710insD) [82]. ORR to afatinib induction was 63%, and MPR reached 50% in those patients who underwent surgery (10% pCR). At 5 years of follow-up, the median PFS was 2.6 years, and the median OS was 5.8 years [82]. The benefit from this perioperative approach was limited by the fact that most patients had a disease recurrence (82%) with a short median time to progression after adjuvant afatinib cessation (2.8 months), similarly to the convergence of DFS Kaplan–Meier curves after discontinuation of adjuvant first- and third-generation EGFR inhibitors [70,82,108,109,110]. Another adverse feature was the high rate of intracranial-only relapses (38%), highlighting the inadequate CNS penetration and activity of first- and second-generation EGFR inhibitors compared to osimertinib and third-generation EGFR inhibitors [69,149]. Even though there are multiple ongoing trials with first- and second-generation inhibitors in the neoadjuvant and perioperative setting, which are included in Table 1, the success of osimertinib in the adjuvant setting fueled the interest and expectations in this field.

The US single-arm Phase 2 NCT03433469 study evaluated up to two months of osimertinib preoperatively in resectable Stage I–IIIA (7th TNM Edition) with *EGFR* common mutations [80]. In total, 24/27 (89%) patients underwent surgical excision, while 3/27 (11%) received definitive chemoradiotherapy because of disease progression (one patient) and refusal due to a requirement for pneumonectomy (two patients). Moreover, 6/24 surgical patients (25%) received adjuvant osimertinib, and 9/24 (37.5%) experienced disease recurrence [80]. The ORR was 52%, and the MPR rate was 14.8% (pCR 0%), which did not meet the prerequisite target of 50% achieved with afatinib. The median DFS in the surgical population was 40.9 months, which was not influenced by MPR [83]. Up to two months of neoadjuvant osimertinib was considered safe and feasible with no delays in surgery. The rate of treatment-related Grade 3/4 AEs was 11.1% (stomatitis, pulmonary embolism, and atrial fibrillation); two patients (7.4%) discontinued treatment; the occurrence of main toxicities—diarrhea and rash—was lower compared to previous generation agents (52% and 40.7%, respectively) [83]. Furthermore, the efficacy and safety of six weeks of neoadjuvant osimertinib were assessed in resectable Stage II–IIIB (T3-4N2) NSCLC with common *EGFR* mutations in the Chinese, single-arm, Phase 2b NEOS (ChiCTR1800016948) [150]. In total, 32/38 patients proceeded to surgical resection, with 30 (93.8%) having complete resection. The ORR was 71.1%, which was the highest observed among all preceding efforts. However, the MPR and pCR rates were 10.7% and 3.6%, respectively, and fell short of expectations. In total, 50% of patients had a rash, 30% had diarrhea, and 30% had stomatitis. Moreover, 3/38 patients (7.5%) had a preoperative treatment-related Grade 3 AE, including hypertension, rashes, and nephrotic syndrome, which did not compromise surgery feasibility. Eight weeks of neoadjuvant osimertinib followed by surgical resection and up to three years of adjuvant osimertinib were evaluated in the Korean, single-arm, Phase 2 NORA study (NCT04816838) in patients with Stage IA–IIA (8th TNM Edition) NSCLC with either *EGFR* exon 19 deletion or L858R point mutation [151]. At a median follow-up of 31 months, the ORR was 44% (11/25, all partial responders), which did not reach the pre-specified cut-off [84]. All participants had R0 resection, and none of them received adjuvant ChT. MPR and pCR rates were 24% (6/25) and 0%, respectively, with no Grade 3 treatment-related AEs during preoperative treatment. The median DFS, EFS, and OS were not reached. Regarding ctDNA and response to treatment, six patients (30%) achieved seroconversion after ctDNA positivity at baseline after one cycle of neoadjuvant osimertinib [84].

Another Chinese, single-arm, Phase 2 trial, the LungMate 007 (NCT04685070), evaluated the efficacy and safety of neoadjuvant third-generation aumolertinib for 8–24 weeks in unresectable Stage III *EGFR*-mutant NSCLC (uncommon mutations were included) with ORR as the primary endpoint [152]. In total, 23/51 (45.1%) of study participants had their disease converted into resectable, underwent complete surgical excision, and received additional therapy post-operatively, either EGFR-TKI monotherapy (20/23), or ChT (2/23) or a combination of the two (1/23) [81]. ORR in the intention-to-treat (ITT) population was 70.6%. In patients with completely resected disease, MPR and pCR rates were 21.7% (5/23) and 13% (3/23), respectively. At 24 months, the median EFS and OS were not reached, while 1-year EFS and OS and 2-year EFS and OS were 88.2%, 98%, 58.8%, and 90.2%, respectively. The most common treatment-related AEs in the ITT population were fatigue (49%), transaminasemia (39.2%), and rash (35.3%), with Grade 3/4 treatment-related AEs occurring in 9.8% of patients [81]. A post hoc analysis of both the ITT and the completely resected population revealed that patients with an exon 19 deletion had a significantly higher ORR and residual tumor cells on pathologic evaluation compared to their L858R point mutation counterparts, highlighting differences in the pathways involved and TME composition between these two major subgroups of *EGFR* alterations [81]. Moreover, a median of 16 weeks of neoadjuvant treatment was the longest among all prospective neoadjuvant–perioperative EGFR-TKI trials. Some patients received up to 24 weeks of treatment of neoadjuvant therapy, and this prolongation beyond 4 months enabled radical surgery in four more participants (17.3% of the surgical population). This improved rate of conversion to resectable disease could be a sign that prolonging the duration of neoadjuvant therapy in *EGFR*-mutant resectable–borderline resectable NSCLC could lead to an improvement in MPR-pCR and thus cure rates, despite the concern of potentially promoting EGFR-TKI resistance and adversely influencing the technical feasibility of surgical excision [81]. The optimal number of cycles of neoadjuvant therapy remains an active field of discussion.

The conclusion stemming from all neoadjuvant EGFR TKI trials is that despite improvements in safety and feasibility with newer-generation agents, monotherapy may not be the optimal approach to improve MPR and pCR rates and, by extension, the likelihood of cure. Specifically, the pathological response rates elicited are rather disappointing when compared to the corresponding ones from the major perioperative CIT trials. MPR ranges from 30.2% with pembrolizumab–ChT in KEYNOTE-671 to 56.2% with tislelizumab–ChT in RATIONALE-315, while pCR ranges from 17.2% with durvalumab–ChT in the AEGEAN to 40.7% again in RATIONALE-315 [27,32,34]. The cytostatic rather than cytotoxic identities of TKIs are most likely responsible for this outcome [112,113]. In light of this “failure” and considering the improved ORR, PFS, and OS rates in advanced disease when other modalities like ChT or the bispecific antibody amivantamab are added to TKIs, combination strategies are currently being evaluated in the next era of ongoing neoadjuvant–perioperative trials for resectable–borderline resectable *EGFR*-mutant NSCLC and are summarized in Table 1 [141,142,143]. However, it is important to emphasize once again that the association between pathological responses and long-term outcomes appears to be less potent in oncogene-driven NSCLC, as patients who do not achieve MPR or pCR can still have excellent long-term survival by continuing highly effective adjuvant TKI treatment [70,71].

An example of a combination strategy in the perioperative setting is currently being evaluated in the ongoing, randomized, multinational (in both Western and Asian populations), Phase 3, NeoADAURA study (NCT04351555) in patients with resectable Stage II–IIIB N2 NSCLC (8th TNM Edition) with common *EGFR* mutations, alone or in combination with uncommon mutations [153]. Patients were randomized with respect to neoadjuvant treatment, with either three cycles of platinum-based ChT plus placebo for ≥9 weeks (comparator arm), osimertinib for ≥9 weeks (experimental arm 1), or osimertinib for ≥9 weeks along with three cycles of platinum-based ChT (experimental arm 2). After surgical resection, osimertinib will be given for up to three years with permission of adjuvant RT and CT [154]. Patients will be stratified by stage, race, and common mutation type. The primary endpoint of NeoADAURA is centrally assessed MPR, while secondary endpoints include EFS, pCR, nodal downstaging, DFS, OS, safety, and quality of life [154]. In total, 92%, 97%, and 89% of the patients who received the combination regimen, osimertinib monotherapy, and ChT only proceeded to surgical resection, respectively. The MPR with the combination approach was 26%, while osimertinib monotherapy has an MPR of 25%. Both were significantly improved compared to the MPR rate of ChT only, which was 2% (difference 24%, OR: 19.82, 95% CI 4.60–85.33, *p* < 0.0001; difference 23%, OR: 19.28, 99.9% CI 1.71–217.39, *p* < 0.0001, respectively) [155]. pCR and 12-month EFS rates were 4%, 9%, and 0% and 93%, 95%, and 83% in the combination, osimertinib, and ChT arm, respectively (EFS osi/ChT vs. ChT HR: 0.50, 99.8% CI 0.17–1.41, *p* = 0.0382). Patients with MPR had significantly fewer EFS events compared to the non-MPR group (2% vs. 18%) [155]. Furthermore, the role of MRD and MRD clearance as potential biomarkers or surrogate endpoints in future studies emerged through the first results of NeoADAURA. In an exploratory analysis, the baseline MRD (before starting neoadjuvant treatment) was significantly correlated with disease extent and EFS as patients with non-detected baseline MRD had less extensive disease and longer EFS compared to their counterparts with detected baseline MRD (12- and 18-month EFS → 96% vs. 89% and 96% vs. 82%, respectively; median DFS HR: 0.24, 95% CI 0.07–0.80) [156]. Patients who received osimertinib, either as monotherapy or as a combination with ChT, had higher rates of pre-surgical MRD clearance compared to patients who received only ChT (84% and 83% vs. 58%, respectively). Finally, pre-surgical MRD clearance was significantly associated with MPR rates (MRD clearance 24% vs. non-clearance 6%, *p* = 0.0378) [156]. These early results from the NeoADAURA trial raised expectations for significant progress in the perioperative management of resectable *EGFR*-mutant NSCLC while awaiting the maturation of data; results from other combination approaches; and the development of fourth-generation EGFR inhibitors in order to overcome resistance to osimertinib and other third-generation inhibitors, as well as agents targeting new molecules like TROP-2, HER-2, and HER-3.

## 5. ALK

The prevalence of *ALK* rearrangements/translocations is estimated to be approximately 3–7% of all NSCLC cases, with no significant or consistent variation due to ethnicity, despite occasional case series reporting a percentage as high as 13% in Asian populations [157,158,159]. These alterations are typically found in younger patients, are strongly associated with adenocarcinoma histology—particularly the acinar variation and the rare signet ring cell pattern—and a never or light-smoker status, and are generally mutually exclusive with other common driver mutations like *EGFR* and *KRAS* [160,161,162,163]. They are also strong predictors of targeted treatment with ALK inhibitors [164,165]. More than 20 fusion partners with *ALK* have been identified. The most common fusion, accounting for most cases (ranging from 54% to 87% in studies), is the intrachromosomal inversion known as Echinoderm microtubule-associated protein-like 4 (*EML4*)-*ALK*, which was reported by Soda et al. in 2007 [162,166,167,168,169,170]. At least 15 variants of *EML4-ALK* fusion have been described and named according to the exons of *EML4* that are fused to *ALK* [171,172,173]. The two most common variants are variant 1 (V1, *EML4* exon 13-*ALK* exon 20) and variant 3a/b (V3a/b, *EML4* exon 6-*ALK* exon 20), with a frequency of 33% and 29%, respectively. Emerging evidence suggests that variants differ in terms of prognosis and responsiveness to targeted treatment with ALK inhibitors [172,173,174]. For instance, V3a/b may be associated with more aggressive features compared to V1 or V2, including a higher propensity for CNS metastasis, poorer prognosis, greater resistance to ALK inhibitors in cell lines, and worse ORR and PFS rates in patient series [174,175].

ALK inhibitors are classified in generations according to their features, like CNS penetration and activity, spectrum of targets, efficacy against resistance mutations, and toxicity profile [176]. Agents belonging to the first three generations are currently approved as a first or subsequent line treatment in advanced or metastatic ALK-rearranged NSCLC [5,6]. The first-generation inhibitor crizotinib was revolutionary when compared to ChT in advanced *ALK*-rearranged or positive NSCLC but had poor CNS penetration, inhibited a multitude of tyrosine kinase receptors besides ALK (including MET and ROS-1), which translated into a plethora of AEs, and had limited efficacy against acquired resistance mutations, with resistance developing rapidly in the first two years [177,178,179]. The subsequent development of second-generation ALK inhibitors (e.g., alectinib, brigatinib, ceritinib, ensartinib), which were more selective against *ALK* rearrangements, offered superior systemic efficacy, significantly improved CNS penetration and activity (alectinib, brigatinib, and ensartinib), and the ability to overcome many acquired single-resistance mutations with respect to crizotinib, like the L1196M gatekeeper mutation, G1269A, and S1206Y, despite significant toxicity, especially with ceritinib [178,180,181,182,183,184]. The third generation of ALK inhibitors, represented mainly by lorlatinib, further improved CNS penetration and activity, was more potent than earlier-generation agents, and retained its efficacy against an even wider range of single-resistance mutations, including the highly refractory to treatment G1202R point mutation [185,186,187,188,189]. A fourth generation of ALK inhibitors, including neladalkib (NVL-655), zotizalkib (TPX-0131), and gilterinib, is currently under investigation and has shown promising results in preclinical and early clinical trials [190,191,192]. Fourth-generation inhibitors, in comparison to earlier molecules, are characterized by an even increased CNS penetration ability, a higher potency and selectivity for ALK by not inhibiting the structurally related tropomyosin receptor kinase (TRK) family, and activity against multiple mutations in the same allele, also known as compound mutations [190,191,192].

Regarding the prognostic influence of *EML4-ALK* rearrangements/translocations in completely resected NSCLC, data are conflicting. Some studies report an unfavorable prognostic effect, with a higher recurrence risk and a trend towards inferior disease outcomes, like a preponderance for CNS relapse [193]. Other studies confer a protective role on the existence of an *EML4-ALK* rearrangement in terms of RFS and OS in the era before ALK inhibition [194], while others report no significant association with DFS or OS [195]. Nonetheless, the recent introduction of adjuvant treatment with an ALK-TKI in completely resected *ALK*-positive NSCLC has reinvigorated the strive for better outcomes in this subgroup of patients, following the paradigm of advanced disease.

### 5.1. Adjuvant Approaches

The advantages gained through the use of first-, second-, and third-generation ALK inhibitors in the advanced setting, along with the insight provided by preceding attempts and lessons in *EGFR*-mutant early NSCLC, prompted the evaluation of these agents in Phase 3 trials. Before the publication of the first successful results with adjuvant alectinib, a small Chinese retrospective study compared adjuvant first-generation crizotinib for two years with adjuvant platinum-based ChT for four cycles in 30 and 29 patients with completely resected Stage IB–IIIA (8th TNM Edition) *ALK*-positive disease, respectively [196]. Importantly, patients with small-cell lung cancer were included, and patients in the ChT group received crizotinib at disease recurrence. Crizotinib led to a statistically significant improvement in DFS (44.5 vs. 14 months, HR: 11.58, 95% CI 5.64–23.76, *p* < 0.0001) and OS (70 vs. 45 months, *p* = 0.021). DFS improvement with crizotinib was significant regardless of the N stage (N0 → HR: 40.58, 95% CI 6.6–249.52, *p* < 0.001; N1-2 → HR: 9.14, 95% CI 4.38–19.08, *p* < 0.001). Targeted therapy with crizotinib was associated with a lower incidence of hematologic toxicity but a higher incidence of QT prolongation, interstitial lung disease, and visual impairment [196].

The Phase 3, open-label, randomized ALINA trial (NCT03456076) evaluated two years of adjuvant second-generation ALK inhibitor alectinib compared to four cycles of platinum-based ChT in patients with completely resected Stage IB–IIIA (T1-3N1-2 or T4N0-1) *ALK*-positive NSCLC according to the 7th TNM Edition [197]. The primary endpoint of the study was the hierarchical estimation of DFS in the II-IIIA and in the ITT population, while the secondary endpoints included CNS DFS, OS, and safety. Unlike the GASTO1002/ICTAN and the ADAURA trial, where all patients received adjuvant ChT before being randomized with respect to EGFR inhibition or a placebo, the ALINA trial juxtaposed alectinib and ChT as sole modalities after surgical resection [70,111,197]. In patients with Stage II–IIIA disease, the 2-year DFS rate was 93.8% with alectinib and 63% with ChT, while the corresponding 3-year DFS rates were 88.3% and 53.3%, respectively [144]. In the ITT population, the 2-year and 3-year DFS rates were 93.6% and 88.7% with alectinib and 63.7% and 54% with ChT, respectively. At the most recent data-cutoff date, the median DFS with alectinib was not reached in either the Stage II–IIIA or the ITT population, while the respective median DFS in the ChT group was 44.4 (HR: 0.24, 95% CI 0.13–0.45, *p* < 0.001) and 41.3 months, respectively (HR: 0.24, 95% CI 0.13–0.43, *p* < 0.001) [144]. The DFS benefit with alectinib was evident across all subgroups according to age, sex, race, and smoking status. In total, 15 patients (11.5%) from the alectinib arm and 49 patients (38.6%) from the ChT arm suffered a disease recurrence, with 4 and 14 participants relapsing in the CNS, respectively. Thus, the 2-year CNS DFS was significantly superior with alectinib compared to ChT (98.4% vs. 85.8%, HR: 0.22, 95% CI 0.08–0.58). In a more recent analysis, adjuvant alectinib keeps conferring a CNS survival benefit in the ITT population with a 4-year CNS DFS rate of 90.4% compared to 76.1% with placebo (HR: 0.37, 95% CI 0.19–0.74) [71]. Toxicity was also improved in the alectinib arm. In total, 18% participants had Grade 3/4 treatment-related AEs with alectinib compared to 27.5% with ChT, with fewer patients discontinuing treatment in the alectinib arm compared to the ChT arm (5.5% and 12.5%, respectively) [144]. Finally, regarding OS results, there was a positive trend in the ITT population with adjuvant alectinib compared to placebo with 4 years of median follow-up (4-year OS 98.4% vs. 92.4%, HR: 0.40, 95% CI 0.12–1.32) [71]. While more mature OS results are pending, alectinib is currently approved in the US, EU, Canada, Japan, and Taiwan for the adjuvant treatment of completely resected, *ALK*-positive Stage IB–IIIA NSCLC, based on the findings of the ALINA trial.

The recently reported interim results from another Phase 3, which evaluated a second-generation ALK inhibitor in the adjuvant setting, corroborate the activity of this subgroup of molecules in early *ALK*-positive NSCLC. In the Chinese double-blind, randomized, ELEVATE trial (NCT05341583), 2 years of adjuvant ensartinib were assessed vs. placebo in patients with completely resected Stage IB–IIIB (only T3N2, 8th TNM Edition) *ALK*-positive NSCLC [198]. Adjuvant ChT was permitted; DFS in the Stage II–IIIB population was the primary endpoint; secondary endpoints included DFS in the ITT population, 3- and 5-year DFS rate, OS, and safety. In a pre-specified interim analysis, with ChT administered postoperatively in 94/137 (68.6%) patients of the experimental arm and in 97/137 (70.8%) patients of the placebo arm, ensartinib led to a significant improvement in the median DFS compared to the placebo in the Stage II–IIIB population (not reached vs. 24.8 months, HR: 0.20, 95% CI 0.11–0.38, *p* < 0.0001) and in the ITT population (not reached vs. 24.8 months, HR: 0.20, 95% CI 0.10–0.37, *p* < 0.0001) [199,200]. The 2-year DFS in patients with Stage II–IIIB disease was 86.4% with ensartinib compared to 53.5% with placebo, while the corresponding rates in the ITT population were 87.3% and 57.2%, respectively. The most common AEs with ensartinib were rash, transaminasemia, constipation, pruritus, and increased creatinine, creatine kinase, lactate dehydrogenase, and alkaline phosphatase. The frequency of Grade 3/4 treatment-related AEs was 35% in the ensartinib arm and 16.8% in the placebo arm, with one Grade 5 event in the ensartinib arm (cerebral hemorrhage) not deemed as treatment-related. Despite dose reduction or treatment interruption occurring in 29.9% and 35% of study participants receiving ensartinib, only 3/137 patients in the ensartinib arm discontinued adjuvant treatment [199,200]. Thus, adjuvant ensartinib after ChT emerges as another option of adjuvant treatment in completely resected early *ALK*-positive NSCLC while we are waiting for DFS results after a longer follow-up period, as well as OS results.

A Chinese real-world retrospective study of alectinib until disease progression or significant toxicity vs. four cycles of platinum-based ChT (68 patients: 19 received alectinib and 49 received ChT) led to less astonishing results when comparing targeted therapy to ChT [201]. The 3-year DFS was 91.7% in the alectinib group and 60.7% in the ChT group, albeit with a borderline significant benefit in DFS (HR: 0.17, 95% CI 0.02–1.28, *p* = 0.051), most likely due to the small sample size. The 5-year OS rate was not reached with alectinib, and 85.2% was reached with ChT, with no significant difference between the two groups (HR: 0.86, 95% CI 0.10–7.63, *p* = 0.89), possibly reflecting the majority of patients in the ChT group receiving an ALK inhibitor upon recurrence. In the IIIA-N2 subgroup, again, alectinib led to a borderline significant improvement in DFS (HR: 0.18, 95% CI, 0.02–1.41, *p* = 0.068) and had a detrimental effect on OS (HR: 1.43, 95% CI 0.13–15.85), which was confounded by the aforementioned factors [201]. However, this retrospective study is not the single counterargument to the favorable results of the ALINA and the ELEVATE trial.

The Adjuvant Lung Cancer Enrichment Marker Identification and Sequencing Trials (ALCHEMIST) screening platform (A151216-NCT02194738) is an umbrella trial undertaken by the ECOG-ACRIN Cancer Research Group, where different strategies are explored as adjuvant treatment for Stage IIA–IIIB (8th TNM Edition) completely resected NSCLC, after optional adjuvant ChT with or without post-operative RT [202]. About 2% of the unselected ALCHEMIST population were *ALK*-positive and were enrolled in the E4512 Phase 3 branch trial [203]. The enrollment in E4512 stopped by the time of the US FDA approval of adjuvant alectinib for completely resected *ALK*-positive NSCLC. In total, 166 patients were recruited in E4512 and were randomized 1:1 to receive either two years of adjuvant crizotinib or a placebo (154 patients were included in the data analysis). The primary endpoint was DFS, and the secondary endpoints included OS and toxicity. The N stage, whether they received radiation, and sex were used as stratification factors. Most patients in both arms underwent a lobectomy and adjuvant ChT. Namely, 58/75 (77%) and 65/75 (87%) in the crizotinib arm underwent a lobectomy and received ChT, while the corresponding numbers in the placebo arm were 66/79 (88%) and 74/79 (95%). The median DFS was 72.8 months with crizotinib and 75.1 months with placebo, with an HR of 1.06 (90% CI 0.63–1.77, *p* = 0.86), and the Kaplan–Meier curves converged and interwined almost from the beginning [203]. Despite immature data, OS was also not significantly improved (HR: 0.49, 90% CI 0.18–1.37, *p* = 0.26). In total, 34/75 (43%) patients in the crizotinib arm had treatment-related AEs (mostly diarrhea, edema, and eye disorders, as well as a Grade 4 stroke). The median duration of treatment with crizotinib was 13.5 (3.4–23.9) months, with only 40% of participants completing the treatment with crizotinib per protocol and 27% of them discontinuing because of toxicity [203].

The underperformance of crizotinib, contrary to the success of alectinib—and most likely of ensartinib—could be explained by a variety of reasons. Firstly, second-generation inhibitors outperform crizotinib in terms of PFS, OS, ORR, and toxicity in the advanced setting due to improved potency and systemic efficacy, greater selectivity for ALK, improved CNS penetration and activity, and delay in the onset of resistance [178,180,181]. Furthermore, the observation-placebo arm in the E4512 trial had an impressive median DFS of 75.1 months, which surpassed the median DFS of the control arm in the ALINA trial by 33.8 months [144,203]. This could be attributed to several factors. The most important among them is the fact that 95% of the placebo arm in the E4512 trial received ChT, which can eradicate micrometastatic disease compared to the cytostatic targeted ALK inhibition, while in the ALINA trial, targeted therapy is tested against ChT. This observation is an argument for a combination–sequential approach in future adjuvant trials with next-generation ALK inhibitors, like in the ELEVATE trial. However, the 2-year DFS rates are comparable in the investigational arm of the ALINA and the ELEVATE study (ITT → 87.3% with ensartinib and 93.6% with alectinib) [144,199]. These rates could render the utility of adjuvant ChT in early *ALK*-positive NSCLC doubtful despite the fact that inter-trial comparison is an improper strategy, mostly because of differences in study populations (ALINA trial used the 7th TNM Edition, participants’ stage ranged from IB to IIIA, and ~55% of the ITT population were of Asian origin, while the ELEVATE trial used the 8th TNM Edition and enrolled only Asian patients of Stage IB–IIIB).

Going back to potential confounding factors responsible for the remarkably better control arm of E4512 compared to ALINA, the baseline scan requirements in the ALINA trial included imaging of the chest, upper abdomen, and CNS, while only chest imaging was compulsory in the E4512 trial, raising suspicion that clinical understaging may have taken place in the latter. Moreover, the interval from surgical resection to randomization in the ALINA trial was 4–12 weeks, while in the E4512 trial, it was significantly longer (4 weeks-11 months), which may have acted as an additional confounding factor. Postoperative treatment with an ALK inhibitor for two years could be regarded as arbitrary, and thus, the optimal duration of adjuvant treatment needs to be defined in future trials. The use of the third-generation lorlatinib could also be explored in the postoperative setting, considering its improved performance compared to older-generation agents [185,186,187,188]. Lastly, (de-)intensification strategies must be deployed in order to avoid unnecessary toxicity in patients potentially cured with surgery only and treat only those at a higher risk for recurrence or those who will truly relapse. The MRD could emerge as a valuable tool in this direction. Some of these issues could be answered in the ongoing Phase 2 and 3 trials, which are summarized in Table 1 and include agents from the first two generations of ALK inhibitors.

### 5.2. Neoadjuvant and Perioperative Approaches

The first evidence concerning the activity of ALK inhibitors in the neoadjuvant–perioperative setting of early *ALK*-positive NSCLC comes from retrospective studies. Zhang et al. evaluated neoadjuvant administration of first-generation crizotinib in Chinese patients with Stage IIIA (N2) *ALK*-positive disease [86]. All 11 patients underwent a surgical resection (lobectomy in 10 and pneumonectomy in 1), 5 patients received adjuvant ChT with or without RT, and 4 patients received adjuvant crizotinib for up to one year. The median duration of neoadjuvant crizotinib was 30 days. In total, 10/11 (91%) had PR, 10/11 (91%) underwent an R0 resection, and 2/11 (18%) had a pCR (one of them suffered Grade 4 hepatotoxicity while on treatment). Six patients had a disease recurrence (two of them after 1 and 2 months of stopping adjuvant crizotinib), with a median DFS of 10.1 months. First-line crizotinib was used in five out of six relapsing patients, with their PFS ranging from 5 to 26 months [86]. Another Chinese single-center study compared “induction” treatment with alectinib to crizotinib in 39 patients with Stage IIIA–IIIB *ALK*-positive NSCLC [204]. Neoadjuvant TKI was given for a median duration of 95 days. In total, 29 patients proceeded to an R0 surgical excision, while 10 had either definitive CRT or continued ALK inhibition as unresected disease. In the surgical cohort, alectinib led to numerically improved pathologic responses compared to crizotinib (MPR → 9/16, 53% vs. 4/13, 30.8%, *p* = 0.26; pCR → 6/16, 37.5% vs. 2/13, 15.4%, *p* = 0.24). At a median follow-up of 3 years, patients treated with alectinib had a significantly longer PFS compared to those who received crizotinib (not reached vs. 17.9 months, *p* = 0.002) and a numerically improved OS (not reached vs. 62.6 months, *p* = 0.226) [204].

Initial efforts for the evaluation of neoadjuvant or perioperative use of ALK inhibitors in prospective trials were hindered by the lack of generalizability of testing for *ALK* rearrangement–translocation in early NSCLC, which, along with their rarity, led to a small number of candidates for enrollment and, thus, a low accrual. The US Phase 2 ARM trial (NCT03088930) aimed to investigate 6 weeks of crizotinib given preoperatively in patients with resectable Stage IA–IIIA (8th TNM Edition) NSCLC with an *ALK* rearrangement, *ROS1* rearrangement, or a *MET*ex14 skipping mutation, with ORR as the primary endpoint [205]. However, only three patients were enrolled, which led to the study’s termination without the publication of results. Likewise, the Japanese, single-arm, Phase 2 SAKULA trial (UMIN00017906), which explored the neoadjuvant use of the second-generation ALK inhibitor ceritinib for three 28-day cycles in patients with Stage IIIA *ALK*-positive disease and had MPR as its primary endpoint, enrolled only seven patients [206]. In total, six out of seven patients (85.7%) managed to complete all three cycles of neoadjuvant treatment, with five out of seven patients (71%) requiring a dose adjustment of ceritinib, which is a second-rate choice due to its high toxicity. ORR was 100%, six out of seven patients (85.7%) proceeded to surgery, and five out of seven (71%) had a complete resection. MPR and pCR were achieved in four (57%) and two (29%) patients, respectively. One patient died because of disease progression, and at a median follow-up of 3 years, the RFS rate was 57% [206].

Early struggles with ALK inhibitors in the neoadjuvant–perioperative setting gave way to progress in the last two years through the report of interim or final results of five prospective Phase 2 studies with second- or third-generation agents. The feasibility of “induction” treatment in locally advanced, Stage III (8th TNM Edition), *ALK*-positive NSCLC with third-generation lorlatinib was proven during the first phase of the Chinese, single-arm LORIN trial (NCT05740943) [207]. The primary endpoint was pCR, and secondary endpoints included PFS, EFS, OS, safety, and ctDNA clearance. In total, 11 out of 13 enrolled patients completed the induction treatment with lorlatinib (10/11 received three 30-day cycles while 1/11 received four cycles) and proceeded to surgical resection (lobectomy) [85]. Four patients had Stage IIIA, eight patients had Stage IIIB, and one patient had Stage IIIC disease. In total, 6/13 (46.2%) of study participants had baseline-detected levels of ctDNA, and all 6 achieved clearance after induction completion. Moreover, 7/13 (53.8%) had N3-unresectable disease at baseline (6 → Stage IIIB, 1 → Stage IIIC). Conversion to resectable disease took place in 6/7 (85.7%) after induction with lorlatinib. ORR was 76.9% (10/13, all partial response). MPR and pCR were encountered in 76.9% (10/13) and 23.1% (3/13) in the ITT population and 90.9% (10/11) and 27.3% (3/11) in the per-protocol population, respectively [85]. The most frequent treatment-related AEs were, as expected, hypercholesterolemia, hypertriglyceridemia, and weight gain, with surgical resection being moderately or severely difficult due to fibrosis or adhesions in 8/11 (72.7%) of patients who proceeded to surgical resection [85]. Currently, the study is recruiting participants for its second phase.

The neoadjuvant use of second-generation ALK inhibitor brigatinib was assessed in the Korean, single-arm, window-of-opportunity WILDERNESS trial (NCT05361564), which was designed with the aim of investigating molecular mechanisms of resistance to ALK inhibition [208]. In total, 12 patients with resectable Stage I–IIIA (8th TNM Edition) *ALK*-positive NSCLC received brigatinib for a median duration of 45 days [89]. All patients proceeded to curative surgery without significant perioperative morbidity. The ORR was 83.3% (10/12), while MPR and pCR were achieved in 58.3% (7/12) and 17% (2/12) of study participants, respectively [89]. Creatine phosphokinase elevation was encountered in six patients (50%). At a median follow-up of almost 2 years, three patients had a disease relapse, with the corresponding 2-year EFS being 70.1% [89]. Furthermore, the Chinese, single-arm, two-stage Neo-INFINITY study (NCT05765877) evaluated the second-generation ALK and ROS1 inhibitor iruplinalkib as perioperative treatment in resectable Stage IB–IIIB (T3N2 only, 8th TNM Edition) *ALK*- or *ROS1*-rearranged NSCLC [209]. The primary endpoint of the study was MPR, and secondary endpoints included pCR, resectability, R0 resection rate, ORR, EFS, DFS, and OS. Iruplinalkib would be administered for 8 weeks preoperatively and for up to 2 years postoperatively [209]. In the first stage of the study, 10 patients were enrolled. An *ALK* rearrangement was found in six out of eight enrolled patients (75%), who completed neoadjuvant treatment and proceeded to surgical resection [210]. All eight patients achieved an objective response and an R0 resection. Concerning the *ALK*-positive participants, an MPR was obtained in 67% (four patients), while a pCR was achieved in 33% (two patients) of the subgroup. Grade 3 transaminasemia was observed in 2/8 (25%) of the whole study population. Based on these promising safety and efficacy data, the second stage of NeoINFINITY is currently ongoing [210].

Alectinib, already established as an adjuvant treatment of surgically resected *ALK*-rearranged NSCLC, was also evaluated in the perioperative setting of early resectable disease. The Italian, single-arm ALNEO trial (NCT05015010) investigated 8 weeks of neoadjuvant and up to 2 years of adjuvant administration of alectinib in potentially resectable, Stage III (N2 or T4N0-1, 8th TNM Edition) *ALK*-positive NSCLC with MPR as the primary endpoint and pCR, ORR, EFS, DFS, OS, and safety included as secondary endpoints [211]. All 33 patients who were enrolled completed the 8 weeks of neoadjuvant treatment, 28 (85%) proceeded to surgery (21 → lobectomy, 3 → pneumonectomy, and 4 → other surgery), 26 (79%) started receiving adjuvant treatment with alectinib, and 5 (15%) managed to complete all 2 years of adjuvant treatment [87]. R0 resection was accomplished in 24/28 (86%) of the surgical cohort. ORR was 67% (22 patients), MPR was observed in 45.4% (15 patients), and pCR was achieved in 12% (4 patients) of the ITT population [87]. At a median follow-up of 15.2 months, 6 (18%) patients experienced a disease recurrence, and 31 (94%) were alive. The median EFS and OS were not reached, and ≥Grade 3 toxicity was limited to three (9%) and two (8%) patients in the preoperative and postoperative phase, respectively, supporting the perioperative use of alectinib despite the noted difficulty in adhering to treatment during the adjuvant phase [87].

Perioperative administration of alectinib is currently explored in the Phase 2 NAUTIKA1 trial (NCT04302025), which is a US umbrella study investigating the tailored use of multiple targeted approaches in resectable Stage IB–IIIB (T3N2 only, 8th TNM Edition) NSCLC harboring actionable molecular alterations [212]. Neoadjuvant treatment with the corresponding targeted agent(s) for 8 weeks is followed by surgical resection, up to four cycles of platinum-based ChT, and up to 2 years of adjuvant targeted therapy with the same agent(s). The molecular alterations used for stratification into each one of the seven included experimental arms were as follows: *ALK* rearrangement, *ROS1* rearrangement, *NTRK1/2/3* fusion, *BRAF* V600 point mutation, *RET* rearrangement, PD-L1 expression in ≥1% of tumor cells, and *KRAS* G12C mutation. The corresponding targeted agents were as follows: alectinib, entrectinib, vemurafenib/cobimetinib, pralsetinib, atezolizumab, and divarasib [212]. In the TKI arms, the primary endpoint is MPR, and the secondary endpoints include pCR, ORR, DFS, EFS, OS, safety, nodal downstaging, and ctDNA clearance rate.

Focusing on the alectinib arm, 33 patients were enrolled, with 69.7% (23) and 6.1% (2) of the participants having Stage IIIA and IIIB disease, respectively [88]. In total, 30 patients received neoadjuvant alectinib, 27/30 (90%) underwent surgical resection (25 had R0 resection), and 21/30 (70%) received adjuvant treatment. ORR was 63.3% (19/30 patients), while MPR and pCR were observed in 60.7% (17) and 25% (7) of the surgical cohort, respectively [88]. Moreover, 33.3% (10/30) of patients from the ITT population achieved a pathologic nodal downstaging. Regarding toxicity and tolerability, only two patients (6.1%) experienced a Grade 3/4 treatment-related AE, and three patients (9.1%) discontinued neoadjuvant treatment with alectinib before its completion [88]. The solely neoadjuvant, Chinese, umbrella Phase 2 NUMER study (NCT06563999), which has a similar design to NAUTIKA-1, is currently ongoing for patients with unresectable Stage III NSCLC with actionable molecular alterations and focuses on resectability rates [213].

The publication of the final survival results of the ALNEO and ALINA trials, as well as the survival results of the NAUTIKA1 study, could possibly assist in defining whether the optimal position of alectinib in resectable *ALK*-positive NSCLC is in the adjuvant or perioperative setting. One of the main advantages of the neoadjuvant-perioperative approach is that it is easier to incorporate (de-)escalation strategies compared to the upfront surgical approach. The main reason for this is that their deployment is based on the achievement of MPR/pCR, which are—at least at the moment—determined with more reliable methods compared to MRD/ctDNA clearance, which could be used to guide the design of these strategies in the adjuvant setting. Moreover, the role of ChT, whether pre- or postoperatively, must be clarified in the algorithm of perioperative management of early *ALK*-positive NSCLC. Contrary to preoperative TKI monotherapy for *EGFR*-mutant resectable NSCLC, the pCR and MPR rates with ALK inhibitor monotherapy range from 12% to 33% and from 45.4% to 67%, respectively, which are comparable to results with perioperative CIT. However, the fact that these rates originate from non-randomized, single-arm Phase 2 trials must be taken into account. Adjuvant ChT could be potentially omitted as well, but there is a lack of pertinent evidence. The arbitrariness of 8 weeks as neoadjuvant treatment with an ALK inhibitor, which is most frequently used across the aforementioned trials, is highlighted in addition to the rest of the points for discussion. The major ongoing perioperative studies in early *ALK*-positive NSCLC are summarized in Table 1.

## 6. The Rest of Actionable Molecular Alterations

The remaining actionable molecular alterations constitute a significant subgroup of the entire NSCLC—mostly adenocarcinoma—cohort, with a cumulative incidence of up to 60% in Western populations and 80% in Asian populations [11,214]. The *KRAS* G12C point mutation occurs in approximately 13% of patients [215], while *KRAS* mutations are identified in up to 30% of NSCLC (the frequency may rise up to 40% in Western populations) [11,214]. The dysregulation of the *MET* gene concerns up to 6% of NSCLC, with the *MET*ex14 skipping mutation occurring in 4% and *MET* amplification in 2% of patients [214,216]. *HER-2* mutations and amplification occur in around 3% and 1%, respectively [214,217,218]. *ROS1* rearrangements/translocations are identified in 1–2% of the NSCLC population [214,219,220]. Less frequent actionable molecular alterations include *BRAF* V600E point mutation (1–2% with the overall *BRAF* mutation rate reaching 5%), *RET* rearrangements/translocations (1–2%), and *NTRK1/2/3* fusions/translocations (0.3–0.5%) [11,214,221,222,223].

When encountered in the advanced metastatic setting, all aforementioned molecular alterations have one or more approved therapeutic choices as a first or subsequent line of treatment, either as monotherapy or in combination with other modalities like ChT, which leads to optimism regarding their potential use perioperatively [5,6,11]. However, there is currently no approved agent or strategy for any of them in the perioperative setting, meaning that the CIT approach is preferred upon occurrence, if indicated. Considering the absence of approved treatment options for early NSCLC besides common *EGFR* mutations and *ALK* rearrangements/translocations, comprehensive testing for the identification of the remaining actionable molecular alterations is currently neither warranted for an optimal management plan nor included in the guidelines of major societies and associations, nor financially compensated according to governmental policies [5,7]. There is currently a significant number of ongoing studies for the perioperative management of NSCLC with alterations other than *EGFR* or *ALK*, with agents being investigated either in single-arm Phase 1 or 2 trials or in larger Phase 3 trials or even in umbrella studies with multiple cohorts, including a multitude of targets like the NAUTIKA1 and NUMER studies [212,213].

The most promising—and anticipated—approaches are encountered in patients with *RET* rearrangements/translocations or a *KRAS* G12C point mutation. The international, randomized Phase 3 LIBRETTO-432 trial (NCT04819100) investigates the adjuvant use of RET inhibitor selpercatinib vs. a placebo after the complete resection—and ChT if needed—of Stage IB–IIIA (8th TNM Edition) *RET*-rearranged NSCLC (EFS as primary endpoint), while cohort 7 of the international, Phase 2, basket trial LIBRETTO-001 (NCT03157128) is still ongoing and aims to evaluate 56 days of neoadjuvant selpercatinib in patients with resectable stage IB-IIIA (8th TNM Edition) *RET*-rearranged NSCLC (MPR as primary endpoint) [224,225,226]. Concerning *KRAS* G12C, its inhibition in preclinical studies led to remodelling of the TME, increased immune activity against the tumor, and synergy with ICIs [227]. These features provide a strong rationale for combination strategies of KRAS G12C inhibitors with IO, some of which are investigated in ongoing studies. The international, randomized, Phase 3 SUNRAY-02 trial (NCT068890598) evaluates the addition of KRAS G12C inhibitor olomorasib for 3 years to PD-1 inhibitor pembrolizumab, with DFS as the primary endpoint, in patients with either completely resected Stage II–IIIB (N2, 8th TNM Edition) *KRAS* G12C-mutant NSCLC with no pCR after preoperative CIT or with the upfront complete resection of Stage II–IIIB disease [228]. There is also a cohort of patients with unresectable Stage III *KRAS* G12C-mutant NSCLC treated with CRT in the SUNRAY-02 trial, where consolidation treatment with 3 years of olomorasib and 1 year of PD-L1 inhibitor durvalumab is compared to the SoC of 1-year consolidation treatment with durvalumab, with PFS as the primary endpoint [228]. Regarding the neoadjuvant setting, the US, single-arm Phase 2 NCT05400577, which evaluated 4 weeks of KRAS G12C inhibitor sotorasib in resectable Stage IB–IIIA (8th TNM Edition) *KRAS* G12C-mutant NSCLC, was terminated due to slow accrual without results [229]. Another neoadjuvant approach with 6 weeks of KRAS G12C inhibitor adagrasib, with or without PD-1 inhibitor nivolumab, in resectable Stage IB–IIIA (8th TNM Edition) *KRAS* G12C-mutant disease is investigated in the ongoing US, open-label Phase 2 Neo-Kan trial (NCT05472623), with pCR as the primary endpoint [230]. Ongoing trials for early NSCLC with molecular alterations besides *EGFR* and *ALK* are included in Table 1.

**Table 1:** Ongoing neoadjuvant, adjuvant, and perioperative Phase 2 and 3 trials in early oncogene-addicted resectable NSCLC.

**Study, Phase**	**Stage, Population**	**Mutation-Target**	**Strategy**	**Study Arm(s)**	**Primary Endpoint**
TARGET (NCT05526755), 2 [140]	II–IIIB (8th TNM Edition), Asian and non-Asian, adjuvant ChT allowed	All *EGFR* mutations apart from exon 20 insertions	Adjuvant	5 years of osimertinib 80 mg vs. placebo after optional ChT	5-year DFS in the common mutation cohort
APPOINT (NCT04922138), 2 [133,134]	IA, Chinese, ≥1 high-risk factor (≥10% of solid, micropapillary, or complex gland components)	All *EGFR* mutations	Adjuvant	3 years of aumolertinib 110 mg vs. placebo	2-year DFS
ADAURA2 (NCT05120349), 3 [135,136]	IA2–IA3 (8th TNM Edition), Asian and non-Asian, high-risk when ≥1 of: >2 cm, LVI, high-grade histology with with ≥20% micropapillary, solid, or complex gland components	*EGFR* common mutations	Adjuvant	3 years of osimertinib 80 mg vs. placebo	DFS in the high-risk subgroup
PACIFIC-4 (NCT03833154), 3 [137]	Medically inoperable I/II (7th TNM Edition), Asian and non-Asian (sub-cohort)	*EGFR* common mutations	Consolidation	3 years of osimertinib 80 mg (single-arm) sub-cohort after curative SBRT	4-year PFS
ICWIP (NCT02125240), 3 [231]	II–IIIA, Chinese (7th TNM Edition)	*EGFR* common mutations	Adjuvant	3 years of icotinib 125 mg three times daily vs. placebo after ChT	3-year DFS
APEX (NCT04762459), 3 [232]	II–IIIA, Chinese (8th TNM Edition)	*EGFR* common with/without uncommon mutations	Adjuvant	3 years of almonertinib 110 mg + 4 cycles of ChT vs. almonertinib 110 mg vs. 4 cycles of ChT	DFS
FORWARD (NCT04853342), 3 [233]	II–IIIA, Chinese	*EGFR* common with/without uncommon mutations	Adjuvant	Furmonertinib 80 mg vs. placebo after optional ChT	DFS
BD-BF-III01 (NCT06041776), 3 [234]	IB–IIIB, Chinese (8th TNM Edition)	*EGFR* common mutations	Adjuvant	3 years of befotertinib 100 mg vs. 2 years of icotinib 125 mg three times daily	5-year DFS
UPLIFT (NCT06955325), 3 [235]	IA–IB (8th TNM Edition), Chinese, high-risk when ≥1 of: low differentiation, solid/micropapillary/complex glandular pattern, vascular or visceral pleural invasion, alveolar space spread, or intermediate/high risk on 14-gene test	*EGFR* common mutations or *ALK* rearrangement–fusion or *ROS1* rearrangement–fusion	Adjuvant	1 year of icotinib 125 mg three times daily or rezivertinib 100 mg or ensartinib 225 mg or 3 years of benmelstobart	DFS
NEOpredict-EGFR (NCT06784791), 2 [236]	Resectable IB–IIIA, European (8th TNM Edition)	*EGFR* common and uncommon mutations with susceptibility to amivantamab	Neoadjuvant	4 weeks of amivantamab + ChT vs. 4 weeks of amivantamab	Feasibility
NOCE01 (NCT05011487), 2 [237]	Resectable IIIA–IIIB (T3-4N2), Chinese (8th TNM Edition)	*EGFR* common with/without uncommon mutations	Neoadjuvant	8 weeks of osimertinib 80 mg + 2 cycles of ChT	ypN0 (complete LN clearance)
NeoIpower (NCT05104788), 2 [238]	Resectable IIA–IIIB (N2), Chinese (8th TNM Edition)	*EGFR* common mutations	Neoadjuvant	8 weeks of icotinib 125 mg three times daily + 2 cycles of ChT	MPR
NCT01470716, 2, [239]	Resectable II–IIIA, Korean (8th TNM Edition)	*EGFR* common mutations	Neoadjuvant	8 weeks of erlotinib 150 mg	PFS
NCT05987826, 2 [240]	Resectable II–IIIB (N2), Chinese (8th TNM Edition)	*EGFR* common with/without uncommon mutations	Neoadjuvant	8 weeks of furmonertinib 80 mg	MPR, DFS
FORESEE (NCT05430802), 2 [241]	Resectable IIIA–IIIB, Chinese (8th TNM Edition)	*EGFR* common with/without uncommon mutations	Neoadjuvant	9 weeks of furmonertinib 80 mg + 3 cycles of ChT	ORR
ANSWER (NCT04455594), 2 [242]	Resectable-potentially resectable IIIA–N2, Chinese (8th TNM Edition)	*EGFR* common with/without uncommon mutations	Neoadjuvant	8 weeks of almonertinib 110 mg vs. investigator’s choice between 8 weeks of erlotinib 150 mg or 3 cycles of ChT	ORR
NCT03203590, 3 [243]	Resectable II–IIIA (7th TNM Edition), Chinese	*EGFR* common and uncommon mutations	Neoadjuvant	8 weeks of gefitinib 250 mg vs. 2 cycles of ChT	2-year DFS
NeoLazer (NCT06268210), 2 [244]	Resectable IB–IIIB, Korean (8th TNM Edition)	*EGFR* common mutations	Perioperative	9 weeks of lazertinib 240 mg + 3 cycles of ChT vs. 9 weeks of lazertinib 240 mg → surgery → 3 years of Lazertinib 240 mg	Primary pathological response (MPR)
NEOAFA (NCT04470076), 2 [245]	Resectable IIA–IIIB, Chinese (8th TNM Edition)	All *EGFR* mutations	Perioperative	8 weeks of afatinib 40 mg + 3 cycles of ChT → surgery → 2 years of afatinib 40 mg	MPR, ORR
NCT03349203, 2 [246]	Potentially resectable IIIB or oligometastatic, Chinese (7th NTM Edition)	*EGFR* common mutations	Perioperative	8 weeks of icotinib 125 mg three times daily → surgery → 2 years of icotinib 125 mg three times daily	ORR
NeolazBAL (NCT05469022), 2 [247]	Resectable I–IIIB or single metastasis IVA, Korean (8th TNM Edition), liquid biopsy for EGFR	*EGFR* common with/without uncommon mutations	Perioperative	9 weeks of lazertinib 240 mg → surgery → 3 years of Lazertinib 240 mg (≥Stage II)	ORR
NCT03749213, 2 [248]	Resectable IIIA–N2, Chinese (8th TNM Edition)	*EGFR* common mutations	Perioperative	8 weeks of icotinib 125 mg three times daily → surgery → 2 years of icotinib 125 mg three times daily	ORR
NCT05132985, 2 [249]	Resectable II–IIIB (N2), Chinese (8th TNM Edition)	*EGFR* common with/without uncommon mutations	Perioperative	8 weeks of icotinib 125 mg three times daily + 2 cycles of ChT → surgery → 2 cycles of ChT + 2 years of icotinib 125 mg three times daily	MPR
NeoADAURA (NCT04351555), 3 [153]	Resectable II–IIIB N2 (8th TNM Edition), Asian and non-Asian	*EGFR* common with/without uncommon mutations	Perioperative	9 weeks of osimertinib 80 mg + 3 cycles of ChT vs. 9 weeks of osimertinib 80 mg vs. 3 cycles of ChT → surgery → 3 years of osimertinib 80 mg	MPR
NEOLA (NCT06194448), 2 [250]	Unresectable III, Asian and non-Asian	*EGFR* common mutations	Induction-consolidation	8 weeks of osimertinib 80 mg → 6 weeks of CRT → osimertinib 80 mg maintenance until progression or unacceptable toxicity	PFS
BD-EN-IV006 (NCT05241028), 2 [251]	IB–IIIA (8th TNM Edition), Chinese	*ALK* rearrangement–fusion	Adjuvant	3 years of ensartinib 225 mg	3-year DFS
NCT05186506, 2 [252]	IIA–IIIA (8th TNM Edition), Chinese	*ALK* rearrangement–fusion	Adjuvant	3 years of ensartinib 225 mg vs. 4 cycles of ChT	DFS
MOTION-NSCLC (NCT06709274), 2 [253]	IA–IB (8th TNM Edition), Chinese	*ALK* rearrangement–fusion	Adjuvant	2 years of alectinib 600 mg twice daily guided by MRD	3-year DFS
NCT06780839, 2 [254]	IA3 with high-risk recurrence factors-IIIB (T3N2 only, 8th TNM Edition), Chinese	*ALK* rearrangement–fusion	Adjuvant	3 years of ensartinib 225 mg +/− ChT according to MRD	DFS
NCT06772610, 2 [255]	I with high-risk recurrence factors (8th TNM Edition), Chinese	*ALK* rearrangement–fusion	Adjuvant	2 years of ensartinib 200 mg	2-year DFS
NCT02201992 (sub-trial of the ALCHEMIST), 3 [256]	IB–IIIA (7th TNM Edition), US	*ALK* rearrangement–fusion	Adjuvant	2 years of crizotinib 250 mg twice daily vs. placebo	OS
ELEVATE (NCT05341583), 3 [198]	IB–IIIB (8th TNM Edition), Chinese, adjuvant ChT allowed	*ALK* rearrangement–fusion	Adjuvant	2 years of ensartinib 225 mg vs. placebo after optional ChT	DFS
E4512 (NCT02194738, sub-trial of the ALCHEMIST), 3 [202]	IIA–IIIB (8th TNM Edition), US, adjuvant ChT allowed	*ALK* rearrangement–fusion	Adjuvant	2 years of crizotinib 250 mg twice daily vs. placebo after optional ChT	DFS
ALINA (NCT03456076), 3 [197]	IB–IIIA (T1-3N1-2 or T4N0-1, 7th TNM Edition), Asian and non-Asian	*ALK* rearrangement–fusion	Adjuvant	2 years of alectinib 600 mg twice daily vs. 4 cycles of ChT	DFS in Stage II–IIIA and ITT population
NCT06862869, 4 [257]	II–IIIB (8th TNM Edition), Chinese	*ALK* rearrangement–fusion	Adjuvant	2 years of alectinib 600 mg twice daily	Real-world DFS
HORIZON 2 (NCT06624059), 3 [258]	Resectable II–IIIB (T3N2 only, 8th TNM Edition), Asian and non-Asian	*ALK* rearrangement–fusion	Adjuvant-perioperative	Surgery → 12 weeks of alectinib 600 mg twice daily + 4 cycles of ChT → 5 years of alectinib 600 mg twice daily (B1 cohort) vs. 12 weeks of alectinib 600 mg twice daily + 3 cycles of ChT → surgery → 5 years of alectinib 600 mg twice daily (B2 cohort)	Safety (B1 cohort) and pCR (B2 cohort)
LORIN (NCT05740943), 2 [207]	Unresectable III (8th TNM Edition), Chinese	*ALK* rearrangement–fusion	Neoadjuvant	12 weeks of lorlatinib 100 mg	pCR
WILDERNESS (NCT05361564), 2 [208]	Resectable I–IIIA (8th TNM Edition), Korean	*ALK* rearrangement–fusion	Neoadjuvant	1 week of brigatinib 90 mg → 3–9 weeks of brigatinib 180 mg	Identification of resistance mechanisms
NUMER (NCT06563999), 2 [213]	Unresectable III (8th TNM Edition), Chinese	*ALK* or *ROS1* or *NTRK1/2/3* or *RET* rearrangement–fusion; *BRAF* V600E or *KRAS* G12C point mutation; *HER-2* mutation or *MET* exon 14 skipping mutation or *EGFR* exon 20 insertion mutation	Neoadjuvant	12 weeks of ensartinib 225 mg or crizotinib 250 mg or larotrectinib 100 mg twice daily or pralsetinib 400 mg or dabrafenib 150 mg twice daily + trametinib 2 mg or glecirasib 800 mg or pyrotinib 400 mg or savolitinib 400–600 mg or sunvozertinib 300 mg	Resectability rate
NEOEAST (NCT05380024), 2 [259]	Resectable IIA–IIIB (T3N2 only, 8th TNM Edition), Chinese	*ALK* rearrangement–fusion	Neoadjuvant	8 weeks of ensartinib 225 mg	MPR
CLOG-003 (NCT06779539), 2 [260]	Resectable II–III (8th TNM Edition), Chinese	*ALK* rearrangement–fusion	Neoadjuvant	12 weeks of ensartinib 225 mg	pCR
NEOLORA (NCT06682884), 2 [261]	Resectable IB–IIIB (including T4N2, 8th TNM Edition), Chinese	*ALK* rearrangement–fusion	Neoadjuvant	6–8 weeks or lorlatinib 100 mg	pCR
NCT06785584, 4 [262]	Resectable-potentially resectable IIA–IIIB (8th TNM Edition), Chinese	*ALK* rearrangement–fusion	Neoadjuvant	8 weeks of ensartinib 225 mg	MPR
NCT06736561, 4 [263]	Resectable-potentially resectable IIA–IIIB (including T4N2, 8th TNM Edition), Chinese	*ALK* rearrangement–fusion	Neoadjuvant	Received neoadjuvant ensartinib in the past or about to receive 6–12 weeks of ensartinib 225 mg	MPR
Neo-INFINITY (NCT05765877), 2 [209]	Resectable IB–IIIB (T3N2 only, 8th TNM Edition), Chinese	*ALK* or *ROS1* rearrangement–fusion	Perioperative	1 week of iruplinalkib 60 mg → 7 weeks of iruplinalkib 180 mg → surgery → 2 years of iruplinalkib 180 mg	MPR
ALNEO (NCT05015010), 2 [211]	Potentially resectable III (N2 or T4N0-1, 8th TNM Edition), Italian	*ALK* rearrangement–fusion	Perioperative	8 weeks of alectinib 600 mg twice daily → surgery → 2 years of alectinib 600 mg twice daily	MPR
NAUTIKA1 (NCT04302025), 2 [212]	Resectable IB–IIIB (T3N2 only, 8th TNM Edition), US	*ALK* or *ROS1* or *NTRK1/2/3* or *RET* rearrangement–fusion; *BRAF* V600 or *KRAS* G12C point mutation; PD-L1 ≥ 1%	Perioperative	8 weeks of alectinib or entrectinib or pralsetinib or vemurafenib + cobimetinib or divarasib or 4 cycles of atezolizumab + low-dose SBRT → surgery → 4 cycles of ChT + 2 years of alectinib or entrectinib or pralsetinib or vemurafenib + cobimetinib or 3 years of divarasib or 16 cycles of atezolizumab	MPR for TKIs, pCR for checkpoint inhibitor, 3–5 Grade 3–5 AEs and delay of surgery for divarasib
LungMate-018 (NCT06282536), 2 [264]	Potentially resectable III–IVA (8th TNM Edition), Chinese	*ALK* or *ROS1* rearrangement–fusion	Perioperative	1 week of iruplinalkib 60 mg → 11 weeks of iruplinalkib 180 mg → surgery → 2 years of iruplinalkib 180 mg	ORR
NCT05118854, 2 [265]	Resectable IIA–IIIB (T3-4N2, 8th TNM Edition), US	*KRAS* G12C point mutation	Neoadjuvant	12 weeks of sotorasib 960 mg + 4 cycles of ChT	MPR
Neo-Kan (NCT05472623), 2 [230]	Resectable, IB–IIIA (8th TNM Edition), US	*KRAS* G12C point mutation	Neoadjuvant	6 weeks of adagrasib 600 mg twice daily vs. 6 weeks of adagrasib 400 mg twice daily + 3 cycles of nivolumab 240 mg	pCR
CCTG I242 (NCT05714891), 2 [266]	Resectable IA2–IIIA (8th TNM Edition), Canadian	*KRAS* G12C point mutation	Perioperative	6 weeks of opnurasib 200 mg twice daily → surgery → 52 weeks of opnurasib 200 mg twice daily after SoC treatment	EFS, MPR
SUNRAY-02 (NCT06890598), 3 [228]	II–IIIB/III unresectable (N2, 8th TNM Edition), Asian and non-Asian	*KRAS* G12C point mutation	Adjuvant/consolidation	1 year of olomorasib 100 mg twice daily + pembrolizumab → 2 years of olomorasib 100 mg twice daily vs. 1 year of pembrolizumab/1 year of olomorasib 100 mg twice daily + durvalumab → 2 years of olomorasib 100 mg twice daily vs. 1 year of durvalumab	DFS/PFS
ECTOP-1033 (NCT07156604), 2 [267]	Resectable IIA–IIIB (N2, 8th TNM Edition), Chinese	*MET*ex14 skipping mutation	Neoadjuvant	8 weeks of vebreltinib 200 mg twice daily	MPR
NEORM (NCT05800340), 2 [268]	Resectable IIB–IIIB (8th TNM Edition), Chinese	*MET*ex14 skipping mutation or *MET* amplification or *HER-2* exon 20 insertion or *RET* rearrangement–fusion or *BRAF* (V600E or non-V600E) mutation	Neoadjuvant	3 cycles of toripalimab 240 mg + ChT	pCR
LungMate-025 (NCT06644313), 2 [269]	Resectable-potentially resectable IIIA–IIIB (N2, 8th TNM Edition), Chinese	*MET*ex14 skipping mutation or *MET* amplification	Perioperative	8–16 weeks of vebreltinib 200 mg twice daily → surgery → 2 years of vebreltinib 200 mg twice daily	ORR
GEOMETRY-N (NCT04926831), 2 [270]	Resectable IB–IIIB (T3-4N2, 8th TNM Edition), US	*MET*ex14 skipping mutation or *MET* amplification	Perioperative	8 weeks of capmatinib 400 mg twice daily → surgery → 3 years of capmatinib 400 mg twice daily	MPR
128TiP (NCT06054191), 2 [271]	Resectable IB–IIIB (T3-4N2, 8th TNM Edition), Chinese	*MET*ex14 skipping mutation or *BRAF* V600 mutation	Perioperative	8 weeks of capmatinib 400 mg twice daily or dabrafenib 150 mg twice daily + trametinib 2 mg → surgery → 2 years of capmatinib 400 mg twice daily or dabrafenib 150 mg twice daily + trametinib 2 mg	pCR
Beamion LUNG-3 (NCT07195695), 3 [272]	II–IIIB (8th TNM Edition), Asian and non-Asian	*HER-2* mutations	Adjuvant	3 years of zongertinib 120–180 mg vs. 1 year of pembrolizumab or atezolizumab or durvalumab or nivolumab	DFS
NEOVISION (NCT06734182), 2 [273]	Resectable II–IIIB (9th TNM Edition), Chinese	*HER-2* mutations	Neoadjuvant	4 cycles of enfavolimab 300 mg + disitamab vedotin 2.5 mg/kg plus ChT	MPR
LIBRETTO-432 (NCT04819100), 3 [224]	IB–IIIA (8th TNM Edition), Asian and non-Asian	*RET* rearrangement–fusion	Adjuvant	3 years of selpercatinib 120–160 mg twice daily vs. placebo	EFS
LIBRETTO-001 (NCT03157128), 2 [225,226]	Cohort 7, resectable IB–IIIA (8th TNM Edition), Asian and non-Asian	*RET* rearrangement–fusion	Neoadjuvant	8 weeks of selpercatinib 160 mg twice daily	MPR

Abbreviations: AE → adverse event; *ALK* → anaplastic lymphoma kinase; *BRAF* → v-Raf murine sarcoma viral oncogene homolog B; ChT → chemotherapy; CRT → concurrent chemoradiotherapy; DFS → disease-free survival; EFS → event-free survival; *EGFR* → epidermal growth factor receptor; *HER-2* → human epidermal growth factor receptor 2; ITT → intention-to-treat; *KRAS* → Kirsten rat sarcoma virus; LN → lymph node; LVI → lymphovascular invasion; MET → mesenchymal–epithelial transition; *METex*14 → MET exon 14; MPR → major pathological response; MRD → minimal residual disease; *NTRK* → neurotrophic tyrosine receptor kinase; ORR → objective response rate; pCR → pathologic complete response; PD-L1 → programmed death-ligand 1; PFS → progression-free survival; *RET* → rearranged during transfection; *ROS1* → proto-oncogene tyrosine-protein kinase ROS; SBRT → stereotactic body radiation therapy; SoC → standard of care; TKI → tyrosine kinase inhibitor.

## 7. Conclusions—Future Perspectives

In summary, the landscape of the management of early NSCLC with actionable molecular alterations has entered a breakthrough era. More than one choice is—or will be soon—available as adjuvant treatment options for common *EGFR* mutations and *ALK* rearrangement–fusion, with the neoadjuvant–perioperative approach gradually gaining ground. Simultaneously, advances in the field of the remaining actionable alterations are eagerly awaited, taking into account that the benefit of targeted approaches in the advanced setting has reinvigorated the strive for success in the early setting as well, leading to a multitude of ongoing trials. A glimpse into the future may reveal rapid and accurate comprehensive molecular testing for all patients with early NSCLC, a new age of perioperative clinical trials with surrogate biomarkers like pCR, MRD, and ctDNA clearance replacing the “old” survival endpoints, the elimination of upfront surgical resection from the algorithm of resectable NSCLC, and neoadjuvant–perioperative strategies employed for the conversion of unresectable disease to resectable. Lastly, concerning the perioperative “sandwich” approach, the rationale for mandatory adjuvant therapy after achieving pCR warrants critical reevaluation. Future efforts must distinguish patients who are truly cured by neoadjuvant intervention, which could allow treatment de-escalation, from those who harbor occult micrometastatic disease and thus would benefit from adjuvant therapy, whether it is standard or intensified.

## Data Availability

No new data were created or analyzed in this study. Data sharing is not applicable to this article.

## Supplementary Materials

The following supporting information can be downloaded at https://www.mdpi.com/article/10.3390/cancers18030493/s1. Table S1: Major Phase 2 and 3 trials of neoadjuvant, adjuvant and perioperative use of immunotherapy (checkpoint inhibitors) in early resectable NSCLC.

## References

[1] Bray F., Laversanne M., Sung H., Ferlay J., Siegel R.L., Soerjomataram I., Jemal A. (2024). Global Cancer Statistics 2022: GLOBOCAN Estimates of Incidence and Mortality Worldwide for 36 Cancers in 185 Countries. CA Cancer J. Clin..

[2] Tsao M.S., Nicholson A.G., Maleszewski J.J., Marx A., Travis W.D. (2022). Introduction to 2021 WHO Classification of Thoracic Tumors. J. Thorac. Oncol..

[3] Travis W.D., Brambilla E., Noguchi M., Nicholson A.G., Geisinger K.R., Yatabe Y., Beer D.G., Powell C.A., Riely G.J., Van Schil P.E. (2011). International Association for the Study of Lung Cancer/American Thoracic Society/European Respiratory Society International Multidisciplinary Classification of Lung Adenocarcinoma. J. Thorac. Oncol..

[4] Jeon H., Wang S., Song J., Gill H., Cheng H. (2025). Update 2025: Management of Non-Small-Cell Lung Cancer. Lung.

[5] (2025). National Comprehensive Cancer Network NCCN Clinical Practice Guidelines in Oncology (NCCN Guidelines^®^): Non–Small Cell Lung Cancer, Version 8. https://www.nccn.org/professionals/physician_gls/pdf/nscl.pdf.

[6] Hendriks L.E., Kerr K.M., Menis J., Mok T.S., Nestle U., Passaro A., Peters S., Planchard D., Smit E.F., Solomon B.J. (2023). Oncogene-Addicted Metastatic Non-Small-Cell Lung Cancer: ESMO Clinical Practice Guideline for Diagnosis, Treatment and Follow-Up☆. Ann. Oncol..

[7] Zer A., Ahn M.-J., Barlesi F., Bubendorf L., De Ruysscher D., Garrido P., Gautschi O., Hendriks L.E., Jänne P.A., Kerr K.M. (2025). Early and Locally Advanced Non-Small-Cell Lung Cancer: ESMO Clinical Practice Guideline for Diagnosis, Treatment and Follow-Up. Ann. Oncol..

[8] Kerr K.M., Bibeau F., Thunnissen E., Botling J., Ryška A., Wolf J., Öhrling K., Burdon P., Malapelle U., Büttner R. (2021). The Evolving Landscape of Biomarker Testing for Non-Small Cell Lung Cancer in Europe. Lung Cancer.

[9] Penault-Llorca F., Kerr K.M., Garrido P., Thunnissen E., Dequeker E., Normanno N., Patton S.J., Fairley J., Kapp J., de Ridder D. (2022). Expert Opinion on NSCLC Small Specimen Biomarker Testing—Part 2: Analysis, Reporting, and Quality Assessment. Virchows Arch..

[10] Carbone D.P., Hirsch F.R., Osarogiagbon R.U., Nishimura K.K., Tsao M.S., Travis W.D., Yang D., Yang S.-R., Yatabe Y., Araujo L.H. (2025). The International Association for the Study of Lung Cancer Staging Project: The Impact of Common Molecular Alterations on Overall Survival in NSCLC in Initial Analyses of the IASLC Ninth Edition Staging Database. J. Thorac. Oncol..

[11] Tan A.C., Tan D.S.W. (2022). Targeted Therapies for Lung Cancer Patients with Oncogenic Driver Molecular Alterations. J. Clin. Oncol..

[12] Rami-Porta R., Wittekind C., Goldstraw P. (2005). Complete Resection in Lung Cancer Surgery: Proposed Definition. Lung Cancer.

[13] Gagliasso M., Migliaretti G., Ardissone F. (2017). Assessing the Prognostic Impact of the International Association for the Study of Lung Cancer Proposed Definitions of Complete, Uncertain, and Incomplete Resection in Non-Small Cell Lung Cancer Surgery. Lung Cancer.

[14] Wolf A., Loo B.W., Mak R.H., Liptay M., Pettiford B., Rocco G., Lanuti M., Merritt R.E., Keshavarz H., Suh R.D. (2025). Systematic Review of Stereotactic Ablative Radiotherapy (SABR)/Stereotactic Body Radiation Therapy (SBRT) for Treatment of High-Risk Patients with Stage I Non-Small Cell Lung Cancer. Semin. Thorac. Cardiovasc. Surg..

[15] Laeseke P., Ng C., Ferko N., Naghi A., Wright G.W.J., Zhang Y., Laidlaw A., Kalsekar I., Laxmanan B., Ghosh S.K. (2023). Stereotactic Body Radiation Therapy and Thermal Ablation for Treatment of NSCLC: A Systematic Literature Review and Meta-Analysis. Lung Cancer.

[16] Wu G.J., Zhang Y., Liang R., Peng L., Zhang S.H. (2025). Comparison of Survival Outcomes of Early-Stage Non-Small-Cell Lung Cancer in Elderly Patients (≥70 Years) Treated with Stereotactic Body Radiotherapy Versus Surgical Resection. World J. Surg..

[17] Ijsseldijk M.A., Shoni M., Siegert C., Wiering B., van Engelenburg A.K.C., Tsai T.C., ten Broek R.P.G., Lebenthal A. (2021). Oncologic Outcomes of Surgery Versus SBRT for Non–Small-Cell Lung Carcinoma: A Systematic Review and Meta-Analysis. Clin. Lung Cancer.

[18] Potter A.L., Costantino C.L., Suliman R.A., Haridas C.S., Senthil P., Kumar A., Mayne N.R., Panda N., Martin L.W., Yang C.F.J. (2023). Recurrence After Complete Resection for Non-Small Cell Lung Cancer in the National Lung Screening Trial. Ann. Thorac. Surg..

[19] Rajaram R., Huang Q., Li R.Z., Chandran U., Zhang Y., Amos T.B., Wright G.W.J., Ferko N.C., Kalsekar I. (2024). Recurrence-Free Survival in Patients with Surgically Resected Non-Small Cell Lung Cancer: A Systematic Literature Review and Meta-Analysis. Chest.

[20] Pignon J.P., Tribodet H., Scagliotti G.V., Douillard J.Y., Shepherd F.A., Stephens R.J., Dunant A., Torri V., Rosell R., Seymour L. (2008). Lung Adjuvant Cisplatin Evaluation: A Pooled Analysis by the LACE Collaborative Group. J. Clin. Oncol..

[21] Burdett S. (2014). Preoperative Chemotherapy for Non-Small-Cell Lung Cancer: A Systematic Review and Meta-Analysis of Individual Participant Data. Lancet.

[22] Felip E., Rosell R., Maestre J.A., Rodríguez-Paniagua J.M., Morán T., Astudillo J., Alonso G., Borro J.M., González-Larriba J.L., Torres A. (2010). Preoperative Chemotherapy plus Surgery versus Surgery plus Adjuvant Chemotherapy versus Surgery Alone in Early-Stage Non-Small-Cell Lung Cancer. J. Clin. Oncol..

[23] Magbanua M.J.M., Swigart L.B., Wu H.T., Hirst G.L., Yau C., Wolf D.M., Tin A., Salari R., Shchegrova S., Pawar H. (2021). Circulating Tumor DNA in Neoadjuvant-Treated Breast Cancer Reflects Response and Survival. Ann. Oncol..

[24] Romero A., Nadal E., Serna R., Insa A., Campelo M.R.G., Benito C., Domine M., Majem M., Abreu D.R., Martinez-Marti A. (2021). OA20.02 Pre-Treatment Levels of CtDNA for Long-Term Survival Prediction in Stage IIIA NSCLC Treated with Neoadjuvant Chemo-Immunotherapy. J. Thorac. Oncol..

[25] Cascone T., Awad M.M., Spicer J.D., He J., Lu S., Sepesi B., Tanaka F., Taube J.M., Cornelissen R., Havel L. (2024). Perioperative Nivolumab in Resectable Lung Cancer. N. Engl. J. Med..

[26] Forde P.M., Spicer J.D., Provencio M., Mitsudomi T., Awad M.M., Wang C., Lu S., Felip E., Swanson S.J., Brahmer J.R. (2025). Overall Survival with Neoadjuvant Nivolumab plus Chemotherapy in Lung Cancer. N. Engl. J. Med..

[27] Spicer J.D., Garassino M.C., Wakelee H., Liberman M., Kato T., Tsuboi M., Lee S.H., Chen K.N., Dooms C., Majem M. (2024). Neoadjuvant Pembrolizumab plus Chemotherapy Followed by Adjuvant Pembrolizumab Compared with Neoadjuvant Chemotherapy Alone in Patients with Early-Stage Non-Small-Cell Lung Cancer (KEYNOTE-671): A Randomised, Double-Blind, Placebo-Controlled, Phase 3 Trial. Lancet.

[28] NSCLC Meta-analyses Collaborative Group (2010). Adjuvant Chemotherapy, with or without Postoperative Radiotherapy, in Operable Non-Small-Cell Lung Cancer: Two Meta-Analyses of Individual Patient Data. Lancet.

[29] Wakelee H., Liberman M., Kato T., Tsuboi M., Lee S.-H., Gao S., Chen K.-N., Dooms C., Majem M., Eigendorff E. (2023). Perioperative Pembrolizumab for Early-Stage Non–Small-Cell Lung Cancer. N. Engl. J. Med..

[30] Mouillet G., Monnet E., Milleron B., Puyraveau M., Quoix E., David P., Ducoloné A., Molinier O., Zalcman G., Depierre A. (2012). Pathologic Complete Response to Preoperative Chemotherapy Predicts Cure in Early-Stage Non-Small-Cell Lung Cancer Combined Analysis of Two IFCT Randomized Trials. J. Thorac. Oncol..

[31] 1243P Pathologic Response as Early Endpoint for Survival Following Neoadjuvant Therapy (NEO-AT) in Resectable Non-Small Cell Lung Cancer (RNSCLC): Systematic Literature Review and Meta-Analysis—Annals of Oncology. https://www.annalsofoncology.org/article/S0923-7534(20)40112-7/fulltext.

[32] Yue D., Wang W., Liu H., Chen Q., Chen C., Liu L., Zhang P., Zhao G., Yang F., Han G. (2025). Perioperative Tislelizumab plus Neoadjuvant Chemotherapy for Patients with Resectable Non-Small-Cell Lung Cancer (RATIONALE-315): An Interim Analysis of a Randomised Clinical Trial. Lancet Respir. Med..

[33] Lu S., Zhang W., Wu L., Wang W., Zhang P., Fang W., Xing W., Chen Q., Yang L., Mei J. (2024). Perioperative Toripalimab Plus Chemotherapy for Patients with Resectable Non-Small Cell Lung Cancer: The Neotorch Randomized Clinical Trial. JAMA.

[34] Heymach J.V., Harpole D., Mitsudomi T., Taube J.M., Galffy G., Hochmair M., Winder T., Zukov R., Garbaos G., Gao S. (2023). Perioperative Durvalumab for Resectable Non-Small-Cell Lung Cancer. N. Engl. J. Med..

[35] Friedman J., Moore E.C., Zolkind P., Robbins Y., Clavijo P.E., Sun L., Greene S., Morisada M.V., Mydlarz W.K., Schmitt N. (2020). Neoadjuvant PD-1 Immune Checkpoint Blockade Reverses Functional Immunodominance among Tumor Antigen-Specific T Cells. Clin. Cancer Res..

[36] Topalian S.L., Taube J.M., Pardoll D.M. (2020). Neoadjuvant Checkpoint Blockade for Cancer Immunotherapy. Science.

[37] Lee J.M., McNamee C.J., Toloza E., Negrao M.V., Lin J., Shum E., Cummings A.L., Kris M.G., Sepesi B., Bara I. (2023). Neoadjuvant Targeted Therapy in Resectable NSCLC: Current and Future Perspectives. J. Thorac. Oncol..

[38] Kim J., Bradford D., Larkins E., Pai-Scherf L.H., Chatterjee S., Mishra-Kalyani P.S., Wearne E., Helms W.S., Ayyoub A., Bi Y. (2021). FDA Approval Summary: Pralsetinib for the Treatment of Lung and Thyroid Cancers with RET Gene Mutations or Fusions. Clin. Cancer Res..

[39] Bradford D., Larkins E., Mushti S.L., Rodriguez L., Skinner A.M., Helms W.S., Lauren S.L.P., Zirkelbach J.F., Li Y., Liu J. (2021). FDA Approval Summary: Selpercatinib for the Treatment of Lung and Thyroid Cancers with RET Gene Mutations or Fusions. Clin. Cancer Res..

[40] Marjanski T., Dziedzic R., Kowalczyk A., Rzyman W. (2021). Safety of Surgery after Neoadjuvant Targeted Therapies in Non-Small Cell Lung Cancer: A Narrative Review. Int. J. Mol. Sci..

[41] Kanemura H., Takeda M., Shimizu S., Nakagawa K. (2021). Interstitial Lung Disease Associated with Capmatinib Therapy in a Patient with Non-Small Cell Lung Cancer Harboring a Skipping Mutation of MET Exon 14. Thorac. Cancer.

[42] Patel S.R., Brown S.-A.N., Kubusek J.E., Mansfield A.S., Duma N. (2020). Osimertinib-Induced Cardiomyopathy. JACC Case Rep..

[43] Ewer M.S., Tekumalla S.H., Walding A., Atuah K.N. (2021). Cardiac Safety of Osimertinib: A Review of Data. J. Clin. Oncol..

[44] Mincu R.I., Mahabadi A.A., Michel L., Mrotzek S.M., Schadendorf D., Rassaf T., Totzeck M. (2019). Cardiovascular Adverse Events Associated with BRAF and MEK Inhibitors: A Systematic Review and Meta-Analysis. JAMA Netw. Open.

[45] Chour A., Denis J., Mascaux C., Zysman M., Bigay-Game L., Swalduz A., Gounant V., Cortot A., Darrason M., Fallet V. (2023). Brief Report: Severe Sotorasib-Related Hepatotoxicity and Non-Liver Adverse Events Associated with Sequential Anti–Programmed Cell Death (Ligand)1 and Sotorasib Therapy in KRASG12C-Mutant Lung Cancer. J. Thorac. Oncol..

[46] Furlepa M., Watts I., Carr A.S. (2025). Management of Triple M Syndrome: A Narrative Review of Immune Checkpoint Inhibitor-Induced Myasthenia Gravis, Myositis and Myocarditis. Cancers.

[47] Provencio M., Nadal E., Insa A., García Campelo R., Casal J., Dómine M., Massuti B., Majem M., Rodríguez-Abreu D., Martínez-Martí A. (2024). Perioperative Chemotherapy and Nivolumab in Non-Small-Cell Lung Cancer (NADIM): 5-Year Clinical Outcomes from a Multicentre, Single-Arm, Phase 2 Trial. Lancet Oncol..

[48] Felip E., Altorki N., Zhou C., Vallières E., Martínez-Martí A., Rittmeyer A., Chella A., Reck M., Goloborodko O., Huang M. (2023). Overall Survival with Adjuvant Atezolizumab after Chemotherapy in Resected Stage II-IIIA Non-Small-Cell Lung Cancer (IMpower010): A Randomised, Multicentre, Open-Label, Phase III Trial. Ann. Oncol..

[49] O’Brien M., Paz-Ares L., Marreaud S., Dafni U., Oselin K., Havel L., Esteban E., Isla D., Martinez-Marti A., Faehling M. (2022). Pembrolizumab versus Placebo as Adjuvant Therapy for Completely Resected Stage IB–IIIA Non-Small-Cell Lung Cancer (PEARLS/KEYNOTE-091): An Interim Analysis of a Randomised, Triple-Blind, Phase 3 Trial. Lancet Oncol..

[50] Terrenato I., Ercolani C., Di Benedetto A., Gallo E., Melucci E., Casini B., Rollo F., Palange A., Visca P., Pescarmona E. (2022). A Real-World Systematic Analysis of Driver Mutations’ Prevalence in Early- and Advanced-Stage NSCLC: Implications for Targeted Therapies in the Adjuvant Setting. Cancers.

[51] Hondelink L.M., Ernst S.M., Atmodimedjo P., Cohen D., Wolf J.L., Dingemans A.M.C., Dubbink H.J., von der Thüsen J.H. (2023). Prevalence, Clinical and Molecular Characteristics of Early Stage EGFR-Mutated Lung Cancer in a Real-Life West-European Cohort: Implications for Adjuvant Therapy. Eur. J. Cancer.

[52] Califano R., Landi L., Cappuzzo F. (2012). Prognostic and Predictive Value of K-RAS Mutations in Non-Small Cell Lung Cancer. Drugs.

[53] Chevallier M., Borgeaud M., Addeo A., Friedlaender A. (2021). Oncogenic Driver Mutations in Non-Small Cell Lung Cancer: Past, Present and Future. World J. Clin. Oncol..

[54] Mizuno T., Arimura T., Kuroda H., Sakakura N., Yatabe Y., Sakao Y. (2018). Current Outcomes of Postrecurrence Survival in Patients after Resection of Non-Small Cell Lung Cancer. J. Thorac. Dis..

[55] Park H.K., Choi Y.D., Yun J.S., Song S.Y., Na K.J., Yoon J.Y., Yoon C.S., Oh H.J., Kim Y.C., Oh I.J. (2023). Genetic Alterations and Risk Factors for Recurrence in Patients with Non-Small Cell Lung Cancer Who Underwent Complete Surgical Resection. Cancers.

[56] Ghazali N., Feng J., Hueniken K., Waddell T.K., Yasufuku K., Pierre A., Donahoe L., Wakeam E., Cypel M., Yeung J. (2024). Analysis of Outcomes in Resected Early-Stage Non-Small Cell Lung Cancer (NSCLC) with Rare Targetable Driver Mutations. J. Clin. Oncol..

[57] Zhou F., Guo H., Xia Y., Le X., Tan D.S.W., Ramalingam S.S., Zhou C. (2025). The Changing Treatment Landscape of EGFR-Mutant Non-Small-Cell Lung Cancer. Nat. Rev. Clin. Oncol..

[58] Mendoza D.P., Lin J.J., Rooney M.M., Chen T., Sequist L.V., Shaw A.T., Digumarthy S.R. (2020). Imaging Features and Metastatic Patterns of Advanced ALK-Rearranged Non-Small Cell Lung Cancer. AJR Am. J. Roentgenol..

[59] Camidge D.R., Kono S.A., Lu X., Okuyama S., Barón A.E., Oton A.B., Davies A.M., Varella-Garcia M., Franklin W., Doebele R.C. (2011). Anaplastic Lymphoma Kinase Gene Rearrangements in Non-Small Cell Lung Cancer Are Associated with Prolonged Progression-Free Survival on Pemetrexed. J. Thorac. Oncol..

[60] Mak R.H., Doran E., Muzikansky A., Kang J., Choi N.C., Willers H., Jackman D.M., Sequist L.V. (2010). Thoracic Radiation Therapy in Locally Advanced NSCLC Patients (Pts) with EGFR Mutations. J. Clin. Oncol..

[61] Soon Y.Y., Vellayappan B., Tey J.C.S., Leong C.N., Koh W.Y., Tham I.W.K. (2017). Impact of Epidermal Growth Factor Receptor Sensitizing Mutations on Outcomes of Patients with Non-Small Cell Lung Cancer Treated with Definitive Thoracic Radiation Therapy: A Systematic Review and Meta-Analysis. Oncotarget.

[62] Nakamura M., Kageyama S.I., Niho S., Okumura M., Hojo H., Motegi A., Nakamura N., Zenda S., Yoh K., Goto K. (2019). Impact of EGFR Mutation and ALK Translocation on Recurrence Pattern After Definitive Chemoradiotherapy for Inoperable Stage III Non-Squamous Non–Small-Cell Lung Cancer. Clin. Lung Cancer.

[63] Chen J., Lu W., Chen M., Cai Z., Zhan P., Liu X., Zhu S., Ye M., Lv T., Lv J. (2024). Efficacy of Immunotherapy in Patients with Oncogene-Driven Non-Small-Cell Lung Cancer: A Systematic Review and Meta-Analysis. Ther. Adv. Med. Oncol..

[64] Li H., Zhang Y., Xu Y., Huang Z., Cheng G., Xie M., Zhou Z., Yu Y., Xi W., Fan Y. (2022). Tumor Immune Microenvironment and Immunotherapy Efficacy in BRAF Mutation Non-Small-Cell Lung Cancer. Cell Death Dis..

[65] Mazieres J., Drilon A., Lusque A., Mhanna L., Cortot A.B., Mezquita L., Thai A.A., Mascaux C., Couraud S., Veillon R. (2019). Immune Checkpoint Inhibitors for Patients with Advanced Lung Cancer and Oncogenic Driver Alterations: Results from the IMMUNOTARGET Registry. Ann. Oncol..

[66] Voruganti T., Marar R., Bleiberg B., Garbo E., Ricciuti B., Parikh K., Aggarwal C. (2025). Perioperative Therapy in Oncogene-Driven Non-Small Cell Lung Cancer: Current Strategies and Unanswered Questions. Am. Soc. Clin. Oncol. Educ. Book.

[67] Li C., Kadeerhan G., Zhang T., Yeerjiang Z., Yang Y., Meng J., Wang D. (2025). Evaluating Pathological Complete Response as an Surrogate Endpoint for Long-Term Survival in Patients with Non-Small Cell Lung Cancer: A Systematic Review and Meta-Analysis. Int. J. Surg..

[68] Ding N., Chen T., Chen Y. (2025). Comment on “Evaluating Pathological Complete Response as an Surrogate Endpoint for Long-Term Survival in Patients with Non-Small Cell Lung Cancer: A Systematic Review and Meta-Analysis”. Int. J. Surg..

[69] Reungwetwattana T., Nakagawa K., Cho B.C., Cobo M., Cho E.K., Bertolini A., Bohnet S., Zhou C., Lee K.H., Nogami N. (2018). CNS Response to Osimertinib versus Standard Epidermal Growth Factor Receptor Tyrosine Kinase Inhibitors in Patients with Untreated EGFR-Mutated Advanced Non-Small-Cell Lung Cancer. J. Clin. Oncol..

[70] Herbst R.S., Wu Y.L., John T., Grohe C., Majem M., Wang J., Kato T., Goldman J.W., Laktionov K., Kim S.W. (2023). Adjuvant Osimertinib for Resected EGFR-Mutated Stage IB-IIIA Non-Small-Cell Lung Cancer: Updated Results From the Phase III Randomized ADAURA Trial. J. Clin. Oncol..

[71] 1787MO—Updated Results from the Phase III ALINA Study of Adjuvant Alectinib vs Chemotherapy (Chemo) in Patients (Pts) with Early-Stage ALK+ Non-Small Cell Lung Cancer (NSCLC). https://cslide.ctimeetingtech.com/play/8L24028p03ot.

[72] Denis M.G., Herbreteau G., Pons-Tostivint E. (2023). Molecular Minimal Residual Disease in Resected Non-Small Cell Lung Cancer (NSCLC): Results of Specifically Designed Interventional Clinical Trials Eagerly Awaited. Transl. Lung Cancer Res..

[73] Boukouris A.E., Michaelidou K., Joosse S.A., Charpidou A., Mavroudis D., Syrigos K.N., Agelaki S. (2025). A Comprehensive Overview of Minimal Residual Disease in the Management of Early-Stage and Locally Advanced Non-Small Cell Lung Cancer. Npj Precis. Oncol..

[74] Zhang J.T., Liu S.Y., Gao W., Liu S.Y.M., Yan H.H., Ji L., Chen Y., Gong Y., Lu H.L., Lin J.T. (2022). Longitudinal Undetectable Molecular Residual Disease Defines Potentially Cured Population in Localized Non-Small Cell Lung Cancer. Cancer Discov..

[75] Wakelee H., Reck M., Felip E., Altorki N.K., Vallieres E., Liersch R., Oizumi S., Tanaka H., Hamm J.T., McCune S. (2024). 1211P IMpower010: CtDNA Status and 5y DFS Follow up in Patients (Pts) with Resected NSCLC Who Received Adjuvant Chemotherapy (Chemo) Followed by Atezolizumab (Atezo) or Best Supportive Care (BSC). Ann. Oncol..

[76] Dickhoff C., Heineman D.J., van Dorp M., Senan S., Bahce I. (2024). The Surgical Resection Difficulty From Neoadjuvant Chemoimmunotherapy Is Minimal and Neoadjuvant Therapy Should Be the Standard. J. Thorac. Oncol..

[77] Ning Y., Bao M., Yan X., Xie D., Jiang G. (2018). Surgery for Advanced Non-Small Cell Lung Cancer Patient after Epidermal Growth Factor Receptor Tyrosine Kinase Inhibitor Neoadjuvant Therapy. Ann. Transl. Med..

[78] Lara-Guerra H., Waddell T.K., Salvarrey M.A., Joshua A.M., Chung C.T., Paul N., Boerner S., Sakurada A., Ludkovski O., Ma C. (2009). Phase II Study of Preoperative Gefitinib in Clinical Stage I Non–Small-Cell Lung Cancer. J. Clin. Oncol..

[79] Zhang Y., Fu F., Hu H., Wang S., Li Y., Hu H., Chen H. (2021). Gefitinib as Neoadjuvant Therapy for Resectable Stage II-IIIA Non–Small Cell Lung Cancer: A Phase II Study. J. Thorac. Cardiovasc. Surg..

[80] Aredo J.V., Urisman A., Gubens M.A., Mulvey C., Allen G.M., Rotow J.K., Kerr D.L., Chakrabarti T., Bacaltos B., Gee M. (2023). Phase II Trial of Neoadjuvant Osimertinib for Surgically Resectable EGFR-Mutated Non-Small Cell Lung Cancer. J. Clin. Oncol..

[81] Bian D., Ji S., Liu Y., Huang Z., Jiang L., Liu M., Bao X., Yang J., Zhou Y., Hu J. (2025). Neoadjuvant Aumolertinib for Unresectable Stage III EGFR-Mutant Non-Small Cell Lung Cancer: A Single-Arm Phase II Trial. Nat. Commun..

[82] Chang A.E.B., Piper-Vallillo A.J., Mak R.H., Lanuti M., Muzikansky A., Rotow J., Jänne P.A., Mino-Kenudson M., Swanson S., Wright C.D. (2024). The ASCENT Trial: A Phase 2 Study of Induction and Consolidation Afatinib and Chemoradiation with or without Surgery in Stage III EGFR-Mutant NSCLC. Oncologist.

[83] Blakely C.M., Urisman A., Gubens M.A., Mulvey C.K., Allen G.M., Shiboski S.C., Rotow J.K., Chakrabarti T., Kerr D.L., Aredo J.V. (2024). Neoadjuvant Osimertinib for the Treatment of Stage I-IIIA Epidermal Growth Factor Receptor-Mutated Non-Small Cell Lung Cancer: A Phase II Multicenter Study. J. Clin. Oncol..

[84] Lee J.B., Choi S.J., Shim H.S., Park B.J., Lee C.Y., Sudhaman S., Velichko S., Hong M.H., Cho B.C., Lim S.M. (2025). Neoadjuvant and Adjuvant Osimertinib in Stage IA to IIIA, EGFR-Mutant NSCLC (NORA). J. Thorac. Oncol..

[85] Zhong W.-Z., Zhang C., Yang Y., Yang J., Zhou Q., Zhong Q., Liang W., Chen Z., Wu Y. (2024). P3.08F.01 Interim Analysis of a Phase 2 Prospective Trial of Induction Lorlatinib in Locally Advanced ALK-Positive NSCLC (LORIN). J. Thorac. Oncol..

[86] Zhang C., Li S.L., Nie Q., Dong S., Shao Y., Yang X.N., Wu Y.L., Yang Y., Zhong W.Z. (2019). Neoadjuvant Crizotinib in Resectable Locally Advanced Non–Small Cell Lung Cancer with ALK Rearrangement. J. Thorac. Oncol..

[87] Leonetti A., Boni L., Gnetti L., Cortinovis D.L., Pasello G., Mazzoni F., Bearz A., Gelsomino F., Passiglia F., Pilotto S. (2025). Alectinib as Neoadjuvant Treatment in Potentially Resectable Stage III ALK-Positive NSCLC: Final Analysis of ALNEO Phase II Trial (GOIRC-01-2020-ML42316). J. Clin. Oncol..

[88] Lee J.M., Negrao M.V., Cummings A., Shum E., Patil T., Lin J., Shu C.A., Saltos A., Florez N., Blakely C.M. (2025). NAUTIKA1: Clinical Outcomes and Pathologic Regression with Neoadjuvant Alectinib in Resectable Stage IB−IIIB ALK+ NSCLC. J. Thorac. Oncol..

[89] Kim C.G., Hong M.H., Kim J., Hong M., Shim H.S., Han M., Kim E.Y., Park B.J., Kim H.E., Yang Y.H. (2025). A Window of Opportunity Study for Preoperative Brigatinib in Resectable Anaplastic Lymphoma Kinase (ALK)-Positive Non-Small-Cell Lung Cancer (NSCLC): WILDERNESS Trial. J. Clin. Oncol..

[90] Pi C., Xu C.R., Zhang M.F., Peng X.X., Wei X.W., Gao X., Yan H.H., Zhou Q. (2018). EGFR Mutations in Early-Stage and Advanced-Stage Lung Adenocarcinoma: Analysis Based on Large-Scale Data from China. Thorac. Cancer.

[91] Gu L., Huang H., Xu Z., Niu X., Li Z., Xia L., Yu Y., Lu S. (2023). Landscape and Predictive Significance of the Structural Classification of EGFR Mutations in Chinese NSCLCs: A Real-World Study. J. Clin. Med..

[92] Robichaux J.P., Le X., Vijayan R.S.K., Hicks J.K., Heeke S., Elamin Y.Y., Lin H.Y., Udagawa H., Skoulidis F., Tran H. (2021). Structure-Based Classification Predicts Drug Response in EGFR-Mutant NSCLC. Nature.

[93] Galvez C., Jacob S., Finkelman B.S., Zhao J., Tegtmeyer K., Chae Y.K., Mohindra N., Salgia R., Jovanovic B., Behdad A. (2020). The Role of EGFR Mutations in Predicting Recurrence in Early and Locally Advanced Lung Adenocarcinoma Following Definitive Therapy. Oncotarget.

[94] Saw S.P.L., Zhou S., Chen J., Lai G., Ang M.K., Chua K., Kanesvaran R., Ng Q.S., Jain A., Tan W.L. (2021). Association of Clinicopathologic and Molecular Tumor Features with Recurrence in Resected Early-Stage Epidermal Growth Factor Receptor-Positive Non-Small Cell Lung Cancer. JAMA Netw. Open.

[95] Mordant P., Brosseau S., Milleron B., Santelmo N., Fraboulet-Moreau S., Besse B., Langlais A., Gossot D., Thomas P.A., Pujol J.L. (2023). Outcome of Patients with Resected Early-Stage Non-Small Cell Lung Cancer and EGFR Mutations: Results From the IFCT Biomarkers France Study. Clin. Lung Cancer.

[96] Zhang Z., Wang T., Zhang J., Cai X., Pan C., Long Y., Chen J., Zhou C., Yin X. (2014). Prognostic Value of Epidermal Growth Factor Receptor Mutations in Resected Non-Small Cell Lung Cancer: A Systematic Review with Meta-Analysis. PLoS ONE.

[97] Zhang S.M., Zhu Q.G., Ding X.X., Lin S., Zhao J., Guan L., Li T., He B., Zhang H.Q. (2018). Prognostic Value of EGFR and KRAS in Resected Non-Small Cell Lung Cancer: A Systematic Review and Meta-Analysis. Cancer Manag. Res..

[98] Janjigian Y.Y., Park B.J., Zakowski M.F., Ladanyi M., Pao W., D’Angelo S.P., Kris M.G., Shen R., Zheng J., Azzoli C.G. (2011). Impact on Disease-Free Survival of Adjuvant Erlotinib or Gefitinib in Patients with Resected Lung Adenocarcinomas That Harbor EGFR Mutations. J. Thorac. Oncol..

[99] D’Angelo S.P., Janjigian Y.Y., Ahye N., Riely G.J., Chaft J.E., Sima C.S., Shen R., Zheng J., Dycoco J., Kris M.G. (2012). Distinct Clinical Course of EGFR-Mutant Resected Lung Cancers: Results of Testing of 1118 Surgical Specimens and Effects of Adjuvant Gefitinib and Erlotinib. J. Thorac. Oncol..

[100] Xie H., Wang H., Xu L., Li M., Peng Y., Cai X., Feng Z., Ren W., Peng Z. (2018). Gefitinib Versus Adjuvant Chemotherapy in Patients with Stage II-IIIA Non–Small-Cell Lung Cancer Harboring Positive EGFR Mutations: A Single-Center Retrospective Study. Clin. Lung Cancer.

[101] Goss G.D., O’Callaghan C., Lorimer I., Tsao M.S., Masters G.A., Jett J., Edelman M.J., Lilenbaum R., Choy H., Khuri F. (2013). Gefitinib versus Placebo in Completely Resected Non-Small-Cell Lung Cancer: Results of the NCIC CTG BR19 Study. J. Clin. Oncol..

[102] Kelly K., Altorki N.K., Eberhardt W.E.E., OBrien M.E.R., Spigel D.R., Crinò L., Tsai C.M., Kim J.H., Cho E.K., Hoffman P.C. (2015). Adjuvant Erlotinib Versus Placebo in Patients with Stage IB-IIIA Non-Small-Cell Lung Cancer (RADIANT): A Randomized, Double-Blind, Phase III Trial. J. Clin. Oncol..

[103] Pennell N.A., Neal J.W., Chaft J.E., Azzoli C.G., Jänne P.A., Govindan R., Evans T.L., Costa D.B., Wakelee H.A., Heist R.S. (2019). SELECT: A Phase II Trial of Adjuvant Erlotinib in Patients with Resected Epidermal Growth Factor Receptor–Mutant Non–Small-Cell Lung Cancer. J. Clin. Oncol..

[104] Wang S.Y., Jiang H., Ou W., Wang B.-X., Zhu T.-F., Chang Z.-H., Hu X.-X. (2025). Adjuvant Icotinib of 12 Months versus Observation as Adjuvant Therapy for Completely Resected EGFR-Mutated Stage IB Non-Small-Cell Lung Cancer: 5-Year Update from CORIN (GASTO1003). J. Clin. Oncol..

[105] Feng S., Wang Y., Cai K., Wu H., Xiong G., Wang H., Zhang Z. (2015). Randomized Adjuvant Chemotherapy of EGFR-Mutated Non-Small Cell Lung Cancer Patients with or without Icotinib Consolidation Therapy. PLoS ONE.

[106] Yue D., Xu S., Wang Q., Li X., Shen Y., Zhao H., Chen C., Mao W., Liu W., Liu J. (2022). Updated Overall Survival and Exploratory Analysis From Randomized, Phase II EVAN Study of Erlotinib Versus Vinorelbine Plus Cisplatin Adjuvant Therapy in Stage IIIA Epidermal Growth Factor Receptor+ Non–Small-Cell Lung Cancer. J. Clin. Oncol..

[107] Li N., Ou W., Ye X., Sun H.B., Zhang L., Fang Q., Zhang S.L., Wang B.X., Wang S.Y. (2014). Pemetrexed-Carboplatin Adjuvant Chemotherapy with or without Gefitinib in Resected Stage IIIA-N2 Non-Small Cell Lung Cancer Harbouring EGFR Mutations: A Randomized, Phase II Study. Ann. Surg. Oncol..

[108] Zhong W.Z., Wang Q., Mao W.M., Xu S.T., Wu L., Wei Y.C., Liu Y.Y., Chen C., Cheng Y., Yin R. (2021). Gefitinib Versus Vinorelbine Plus Cisplatin as Adjuvant Treatment for Stage II-IIIA (N1-N2) EGFR-Mutant NSCLC: Final Overall Survival Analysis of CTONG1104 Phase III Trial. J. Clin. Oncol..

[109] Tada H., Mitsudomi T., Misumi T., Sugio K., Tsuboi M., Okamoto I., Iwamoto Y., Sakakura N., Sugawara S., Atagi S. (2022). Randomized Phase III Study of Gefitinib Versus Cisplatin Plus Vinorelbine for Patients with Resected Stage II-IIIA Non–Small-Cell Lung Cancer with EGFR Mutation (IMPACT). J. Clin. Oncol..

[110] He J., Su C., Liang W., Xu S., Wu L., Fu X., Zhang X., Ge D., Chen Q., Mao W. (2021). Icotinib versus Chemotherapy as Adjuvant Treatment for Stage II–IIIA EGFR-Mutant Non-Small-Cell Lung Cancer (EVIDENCE): A Randomised, Open-Label, Phase 3 Trial. Lancet Respir. Med..

[111] Li N., Ou W., Cheng C., You J., Yang L., Chen F.X., Liang Y., Yang Z., Wang B.X., Chang Z.H. (2025). Adjuvant Icotinib for Resected EGFR-Mutated Stage II–IIIA Non-Small-Cell Lung Cancer (ICTAN, GASTO1002): A Randomized Comparison Study. Signal Transduct. Target. Ther..

[112] Remon J., Saw S.P.L., Cortiula F., Singh P.K., Menis J., Mountzios G., Hendriks L.E.L. (2024). Perioperative Treatment Strategies in EGFR-Mutant Early-Stage NSCLC: Current Evidence and Future Challenges. J. Thorac. Oncol..

[113] La Monica S., Minari R., Cretella D., Flammini L., Fumarola C., Bonelli M., Cavazzoni A., Digiacomo G., Galetti M., Madeddu D. (2019). Third Generation EGFR Inhibitor Osimertinib Combined with Pemetrexed or Cisplatin Exerts Long-Lasting Anti-Tumor Effect in EGFR-Mutated Pre-Clinical Models of NSCLC. J. Exp. Clin. Cancer Res..

[114] Yu H.A., Arcila M.E., Rekhtman N., Sima C.S., Zakowski M.F., Pao W., Kris M.G., Miller V.A., Ladanyi M., Riely G.J. (2013). Analysis of Tumor Specimens at the Time of Acquired Resistance to EGFR-TKI Therapy in 155 Patients with EGFR-Mutant Lung Cancers. Clin. Cancer Res..

[115] Bencze E., Bogos K., Kohánka A., Báthory-Fülöp L., Sárosi V., Csernák E., Bittner N., Melegh Z., Tóth E. (2022). EGFR T790M Mutation Detection in Patients with Non-Small Cell Lung Cancer After First Line EGFR TKI Therapy: Summary of Results in a Three-Year Period and a Comparison of Commercially Available Detection Kits. Pathol. Oncol. Res..

[116] Soria J.-C., Ohe Y., Vansteenkiste J., Reungwetwattana T., Chewaskulyong B., Lee K.H., Dechaphunkul A., Imamura F., Nogami N., Kurata T. (2018). Osimertinib in Untreated EGFR-Mutated Advanced Non-Small-Cell Lung Cancer. N. Engl. J. Med..

[117] Cheng Y., He Y., Li W., Zhang H.L., Zhou Q., Wang B., Liu C., Walding A., Saggese M., Huang X. (2021). Osimertinib Versus Comparator EGFR TKI as First-Line Treatment for EGFR-Mutated Advanced NSCLC: FLAURA China, A Randomized Study. Target. Oncol..

[118] Tsuboi M., Herbst R.S., John T., Kato T., Majem M., Grohé C., Wang J., Goldman J.W., Lu S., Su W.-C. (2023). Overall Survival with Osimertinib in Resected EGFR-Mutated NSCLC. N. Engl. J. Med..

[119] Gale D., Heider K., Ruiz-Valdepenas A., Hackinger S., Perry M., Marsico G., Rundell V., Wulff J., Sharma G., Knock H. (2022). Residual CtDNA after Treatment Predicts Early Relapse in Patients with Early-Stage Non-Small Cell Lung Cancer. Ann. Oncol..

[120] Chaudhuri A.A., Chabon J.J., Lovejoy A.F., Newman A.M., Stehr H., Azad T.D., Khodadoust M.S., Esfahani M.S., Liu C.L., Zhou L. (2017). Early Detection of Molecular Residual Disease in Localized Lung Cancer by Circulating Tumor DNA Profiling. Cancer Discov..

[121] Tian X., Liu X., Wang K., Wang R., Li Y., Qian K., Wang T., Zhao X., Liu L., Zhang P.L. (2024). Postoperative CtDNA in Indicating the Recurrence Risk and Monitoring the Effect of Adjuvant Therapy in Surgical Non-Small Cell Lung Cancer. Thorac. Cancer.

[122] Masago K., Horio Y., Fujita S., Yatabe Y. (2019). Minimal Residual Disease after Radical Surgery in EGFR-Mutant Non-Small Cell Lung Cancer. Transl. Lung Cancer Res..

[123] Jung H.A., Ku B.M., Kim Y.J., Park S., Sun J.M., Lee S.H., Ahn J.S., Cho J.H., Kim H.K., Choi Y.S. (2023). Longitudinal Monitoring of Circulating Tumor DNA From Plasma in Patients with Curative Resected Stages I to IIIA EGFR-Mutant Non–Small Cell Lung Cancer. J. Thorac. Oncol..

[124] Herbst R.S., John T., Grohé C., Goldman J.W., Kato T., Laktionov K., Bonanno L., Tiseo M., Majem M., Dómine M. (2025). Molecular Residual Disease Analysis of Adjuvant Osimertinib in Resected EGFR-Mutated Stage IB–IIIA Non-Small-Cell Lung Cancer. Nat. Med..

[125] Xia L., Mei J., Kang R., Deng S., Chen Y., Yang Y., Feng G., Deng Y., Gan F., Lin Y. (2022). Perioperative CtDNA-Based Molecular Residual Disease Detection for Non-Small Cell Lung Cancer: A Prospective Multicenter Cohort Study (LUNGCA-1). Clin. Cancer Res..

[126] Ferrara M.G., Belluomini L., Smimmo A., Sposito M., Avancini A., Giannarelli D., Milella M., Pilotto S., Bria E. (2023). Meta-Analysis of the Prognostic Impact of TP53 Co-Mutations in EGFR-Mutant Advanced Non-Small-Cell Lung Cancer Treated with Tyrosine Kinase Inhibitors. Crit. Rev. Oncol. Hematol..

[127] Lee J.K., Lee J., Kim S., Kim S., Youk J., Park S., An Y., Keam B., Kim D.W., Heo D.S. (2017). Clonal History and Genetic Predictors of Transformation Into Small-Cell Carcinomas From Lung Adenocarcinomas. J. Clin. Oncol..

[128] Offin M., Chan J.M., Tenet M., Rizvi H.A., Shen R., Riely G.J., Rekhtman N., Daneshbod Y., Quintanal-Villalonga A., Penson A. (2019). Concurrent RB1 and TP53 Alterations Define a Subset of EGFR-Mutant Lung Cancers at Risk for Histologic Transformation and Inferior Clinical Outcomes. J. Thorac. Oncol..

[129] Study Details|NCT04500717|Almonertinib Plus Chemotherapy as First-Line Treatment in Patients with EGFR Concomitant Tumor Suppressor Gene Mutation|ClinicalTrials.Gov. NCT04500717.

[130] Search ClinicalTrials.Gov for: NCT03567642|Card Results|ClinicalTrials.Gov. NCT03567642.

[131] Jung H.A., Lim J., Choi Y.L., Lee S.H., Joung J.G., Jeon Y.J., Choi J.W., Shin S., Cho J.H., Kim H.K. (2022). Clinical, Pathologic, and Molecular Prognostic Factors in Patients with Early-Stage EGFR-Mutant NSCLC. Clin. Cancer Res..

[132] Liu S.Y., Bao H., Wang Q., Mao W.M., Chen Y., Tong X., Xu S.T., Wu L., Wei Y.C., Liu Y.Y. (2021). Genomic Signatures Define Three Subtypes of EGFR-Mutant Stage II-III Non-Small-Cell Lung Cancer with Distinct Adjuvant Therapy Outcomes. Nat. Commun..

[133] Han B., Fang W.V., Chen C., Zhang W., Zhang B., Yao F., Shi J.-X., Yue D., Qi Y., Zhu Z. (2025). 145P: Efficacy and Safety of Aumolertinib as Adjuvant Therapy in Resectable Stage I A NSCLC with High-Risk Factors and EGFRsensitizing Mutations (APPOINT). J. Thorac. Oncol..

[134] Zhang W., Fang W., Chen C., Zhang B., Yao F., Shi J., Yue D., Qi Y., Zhu Z., Xie J. (2025). Efficacy and Safety of Aumolertinib as Adjuvant Therapy in Resectable Stage IA EGFR Mutated NSCLC with High Risk (APPOINT). J. Thorac. Oncol..

[135] Study Details|NCT05120349|A Global Study to Assess the Effects of Osimertinib in Participants with EGFRm Stage IA2-IA3 NSCLC Following Complete Tumour Resection|ClinicalTrials.Gov. NCT05120349.

[136] Tsutani Y., Goldman J.W., Dacic S., Yatabe Y., Majem M., Huang X., Chen A., van der Gronde T., He J. (2023). Adjuvant Osimertinib vs. Placebo in Completely Resected Stage IA2–IA3 EGFR-Mutated NSCLC: ADAURA2. Clin. Lung Cancer.

[137] Study Details|NCT03833154|Durvalumab with Stereotactic Body Radiation Therapy (SBRT) vs Placebo with SBRT in Early Stage Unresected Non-Small Cell Lung Cancer (NSCLC) Patients/Osimertinib Following SBRT in Patients with Early Stage Unresected NSCLC Harboring an EGFR Mutation|ClinicalTrials.Gov. NCT03833154.

[138] Dong R.F., Zhu M.L., Liu M.M., Xu Y.T., Yuan L.L., Bian J., Xia Y.Z., Kong L.Y. (2021). EGFR mutation mediates resistance to EGFR tyrosine kinase inhibitors in NSCLC: From molecular mechanisms to clinical research. Pharmacol Res..

[139] Lv C., Wang R., Li S., Yan S., Wang Y., Chen J., Wang L., Liu Y., Guo Z., Wang J. (2023). Randomized Phase II Adjuvant Trial to Compare Two Treatment Durations of Icotinib (2 Years versus 1 Year) for Stage II-IIIA EGFR-Positive Lung Adenocarcinoma Patients (ICOMPARE Study). ESMO Open.

[140] Soo R.A., de Marinis F., Han J.Y., Ho J.C.M., Martin E., Servidio L., Sandelin M., Popat S. (2024). TARGET: A Phase II, Open-Label, Single-Arm Study of 5-Year Adjuvant Osimertinib in Completely Resected EGFR-Mutated Stage II to IIIB NSCLC Post Complete Surgical Resection. Clin. Lung Cancer.

[141] Cho B.C., Lu S., Felip E., Spira A.I., Girard N., Lee J.-S., Lee S.-H., Ostapenko Y., Danchaivijitr P., Liu B. (2024). Amivantamab plus Lazertinib in Previously Untreated EGFR-Mutated Advanced NSCLC. N. Engl. J. Med..

[142] Passaro A., Wang J., Wang Y., Lee S.H., Melosky B., Shih J.Y., Azuma K., Juan-Vidal O., Cobo M., Felip E. (2024). Amivantamab plus Chemotherapy with and without Lazertinib in EGFR-Mutant Advanced NSCLC after Disease Progression on Osimertinib: Primary Results from the Phase III MARIPOSA-2 Study. Ann. Oncol..

[143] Planchard D., Jänne P.A., Cheng Y., Yang J.C.-H., Yanagitani N., Kim S.-W., Sugawara S., Yu Y., Fan Y., Geater S.L. (2023). Osimertinib with or without Chemotherapy in EGFR-Mutated Advanced NSCLC. N. Engl. J. Med..

[144] Wu Y.-L., Dziadziuszko R., Ahn J.S., Barlesi F., Nishio M., Lee D.H., Lee J.-S., Zhong W., Horinouchi H., Mao W. (2024). Alectinib in Resected ALK-Positive Non–Small-Cell Lung Cancer. N. Engl. J. Med..

[145] Xiong L., Lou Y., Bai H., Li R., Xia J., Fang W., Zhang J., Han-Zhang H., Lizaso A., Li B. (2020). Efficacy of Erlotinib as Neoadjuvant Regimen in EGFR-Mutant Locally Advanced Non-Small Cell Lung Cancer Patients. J. Int. Med. Res..

[146] Zhong W.Z., Chen K.N., Chen C., Gu C.D., Wang J., Yang X.N., Mao W.M., Wang Q., Qiao G.B., Cheng Y. (2019). Erlotinib Versus Gemcitabine Plus Cisplatin as Neoadjuvant Treatment of Stage IIIA-N2 EGFR-Mutant Non-Small-Cell Lung Cancer (EMERGING-CTONG 1103): A Randomized Phase II Study. J. Clin. Oncol..

[147] Zhong W.Z., Yan H.H., Chen K.N., Chen C., Gu C.D., Wang J., Yang X.N., Mao W.M., Wang Q., Qiao G.B. (2023). Erlotinib versus Gemcitabine plus Cisplatin as Neoadjuvant Treatment of Stage IIIA-N2 EGFR-Mutant Non-Small-Cell Lung Cancer: Final Overall Survival Analysis of the EMERGING-CTONG 1103 Randomised Phase II Trial. Signal Transduct. Target. Ther..

[148] Bian D., Sun L., Hu J., Duan L., Xia H., Zhu X., Sun F., Zhang L., Yu H., Xiong Y. (2023). Neoadjuvant Afatinib for Stage III EGFR-Mutant Non-Small Cell Lung Cancer: A Phase II Study. Nat. Commun..

[149] Ahluwalia M.S., Becker K., Levy B.P. (2018). Epidermal Growth Factor Receptor Tyrosine Kinase Inhibitors for Central Nervous System Metastases from Non-Small Cell Lung Cancer. Oncologist.

[150] Lv C., Fang W., Wu N., Jiao W., Xu S., Ma H., Wang J., Wang R., Ji C., Li S. (2023). Osimertinib as Neoadjuvant Therapy in Patients with EGFR-Mutant Resectable Stage II-IIIB Lung Adenocarcinoma (NEOS): A Multicenter, Single-Arm, Open-Label Phase 2b Trial. Lung Cancer.

[151] Study Details|NCT04816838|A Window of Opportunity Study for Investigating Drug Tolerant Persister (DTP) to Neoadjuvant Osimertinib in Resectable Non-Small Cell Lung Cancer (NSCLC) Harbouring EGFR Mutations|ClinicalTrials.Gov. NCT04816838.

[152] Study Details|NCT04685070|Neoadjuvant HS-10296 (Almonertinib) Therapy for Potential Resectable Stage III EGFR Mutation-Positive Non-Small Cell Lung Cancer|ClinicalTrials.Gov. NCT04685070.

[153] Study Details|NCT04351555|A Study of Osimertinib with or Without Chemotherapy Versus Chemotherapy Alone as Neoadjuvant Therapy for Patients with EGFRm Positive Resectable Non-Small Cell Lung Cancer|ClinicalTrials.Gov. NCT04351555.

[154] Tsuboi M., Weder W., Escriu C., Blakely C., He J., Dacic S., Yatabe Y., Zeng L., Walding A., Chaft J.E. (2021). Neoadjuvant Osimertinib With/Without Chemotherapy Versus Chemotherapy Alone for EGFR-Mutated Resectable Non-Small-Cell Lung Cancer: NeoADAURA. Future Oncol..

[155] Chaft J.E., Weder W., He J., Chen K., Hochmair M., Shih J.Y., Lee S.Y., Lee K.Y., Nhung N., Saeteng S. (2025). Neoadjuvant (neoadj) Osimertinib (Osi) ± Chemotherapy (CT) vs CT Alone in Resectable (R) Epidermal Growth Factor Receptor-Mutated (EGFRm) NSCLC: NeoADAURA. J. Clin. Oncol..

[156] Blakely C.M., Robichaux J., Yong Lee S., Shih J.-Y., Lee K.-Y., Viet Nhung N., Saeteng S., Chaft J.E., He J., Weder W. (2025). Molecular Residual Disease (MRD) Analysis from NeoADAURA: Neoadjuvant Osimertinib ± Chemotherapy in Resectable EGFRm NSCLC. J. Thorac. Oncol..

[157] Takeuchi K., Choi Y.L., Soda M., Inamura K., Togashi Y., Hatano S., Enomoto M., Takada S., Yamashita Y., Satoh Y. (2008). Multiplex Reverse Transcription-PCR Screening for EML4-ALK Fusion Transcripts. Clin. Cancer Res..

[158] Koivunen J.P., Mermel C., Zejnullahu K., Murphy C., Lifshits E., Holmes A.J., Choi H.G., Kim J., Chiang D., Thomas R. (2008). EML4-ALK Fusion Gene and Efficacy of an ALK Kinase Inhibitor in Lung Cancer. Clin. Cancer Res..

[159] Shaw A.T., Yeap B.Y., Mino-Kenudson M., Digumarthy S.R., Costa D.B., Heist R.S., Solomon B., Stubbs H., Admane S., McDermott U. (2009). Clinical Features and Outcome of Patients with Non-Small-Cell Lung Cancer Who Harbor EML4-ALK. J. Clin. Oncol..

[160] Peters S., Taron M., Bubendorf L., Blackhall F., Stahel R. (2013). Treatment and Detection of ALK-Rearranged NSCLC. Lung Cancer.

[161] Scagliotti G., Stahel R.A., Rosell R., Thatcher N., Soria J.C. (2012). ALK Translocation and Crizotinib in Non-Small Cell Lung Cancer: An Evolving Paradigm in Oncology Drug Development. Eur. J. Cancer.

[162] Soda M., Choi Y.L., Enomoto M., Takada S., Yamashita Y., Ishikawa S., Fujiwara S.I., Watanabe H., Kurashina K., Hatanaka H. (2007). Identification of the Transforming EML4-ALK Fusion Gene in Non-Small-Cell Lung Cancer. Nature.

[163] Inamura K., Takeuchi K., Togashi Y., Hatano S., Ninomiya H., Motoi N., Mun M.Y., Sakao Y., Okumura S., Nakagawa K. (2009). EML4-ALK Lung Cancers Are Characterized by Rare Other Mutations, a TTF-1 Cell Lineage, an Acinar Histology, and Young Onset. Mod. Pathol..

[164] Le T., Gerber D.E. (2017). ALK Alterations and Inhibition in Lung Cancer. Semin. Cancer Biol..

[165] Poei D., Ali S., Ye S., Hsu R. (2024). ALK Inhibitors in Cancer: Mechanisms of Resistance and Therapeutic Management Strategies. Cancer Drug Resist..

[166] Lei Y., Lei Y., Shi X., Wang J. (2022). EML4-ALK Fusion Gene in Non-Small Cell Lung Cancer. Oncol. Lett..

[167] Kang J., Zhang X.C., Chen H.J., Zhong W.Z., Xu Y., Su J., Zhou Q., Tu H.Y., Wang Z., Xu C.R. (2020). Complex ALK Fusions Are Associated with Better Prognosis in Advanced Non-Small Cell Lung Cancer. Front. Oncol..

[168] Wei Q., Zhang Y., Wang Y., Desai A., Tan S., Huang Q., Pu X., Tian P., Li Y. (2023). Superior Clinical Outcomes in Patients with Non-Small Cell Lung Cancer Harboring Multiple ALK Fusions Treated with Tyrosine Kinase Inhibitors. Transl. Lung Cancer Res..

[169] Ren W., Zhang B., Ma J., Li W., Lan J., Men H., Zhang Q. (2015). EML4-ALK Translocation Is Associated with Early Onset of Disease and Other Clinicopathological Features in Chinese Female Never-Smokers with Non-Small-Cell Lung Cancer. Oncol. Lett..

[170] He Y., Sun L.Y., Gong R., Liu Q., Long Y.K., Liu F., Wang F. (2019). The Prevalence of EML4-ALK Variants in Patients with Non-Small-Cell Lung Cancer: A Systematic Review and Meta-Analysis. Biomark. Med..

[171] Sabir S.R., Yeoh S., Jackson G., Bayliss R. (2017). EML4-ALK Variants: Biological and Molecular Properties, and the Implications for Patients. Cancers.

[172] Zhang S.S., Nagasaka M., Zhu V.W., Ou S.H.I. (2021). Going beneath the Tip of the Iceberg. Identifying and Understanding EML4-ALK Variants and TP53 Mutations to Optimize Treatment of ALK Fusion Positive (ALK+) NSCLC. Lung Cancer.

[173] Sasaki T., Rodig S.J., Chirieac L.R., Jänne P.A. (2010). The Biology and Treatment of EML4-ALK Non-Small Cell Lung Cancer. Eur. J. Cancer.

[174] Zou Z., Wu L., Hao X., Li Y., Liang L., Gu Y., Ying J., Li J., Xing P. (2025). Impact of EML4-ALK Variants and TP53 Status on the Efficacy of ALK Inhibitors in Patients with Non-Small Cell Lung Cancer. Thorac. Cancer.

[175] Woo C.G., Seo S., Kim S.W., Jang S.J., Park K.S., Song J.Y., Lee B., Richards M.W., Bayliss R., Lee D.H. (2017). Differential Protein Stability and Clinical Responses of EML4-ALK Fusion Variants to Various ALK Inhibitors in Advanced ALK-Rearranged Non-Small Cell Lung Cancer. Ann. Oncol..

[176] Overview of Systematic Reviews of ICIs in NSCLC with EGFR, ALK, ROS1, and RET Actionable Driver Mutations: Drugs [Internet]—PubMed. https://pubmed.ncbi.nlm.nih.gov/39693461/.

[177] Wang M., Wang G., Ma H., Shan B. (2019). Crizotinib Versus Chemotherapy on ALK-Positive NSCLC: A Systematic Review of Efficacy and Safety. Curr. Cancer Drug Targets.

[178] Luo Y., Zhang Z., Guo X.Z., Tang X., Li S., Gong G., Gao S., Zhang Y., Lin S. (2023). Comparative Safety of Anaplastic Lymphoma Kinase Tyrosine Kinase Inhibitors in Advanced Anaplastic Lymphoma Kinase-Mutated Non-Small Cell Lung Cancer: Systematic Review and Network Meta-Analysis. Lung Cancer.

[179] Cameron L.B., Hitchen N., Chandran E., Morris T., Manser R., Solomon B.J., Jordan V. (2022). Targeted Therapy for Advanced Anaplastic Lymphoma Kinase (ALK)-Rearranged Non-Small Cell Lung Cancer. Cochrane Database Syst. Rev..

[180] Yang Y.L., Xiang Z.J., Yang J.H., Wang W.J., Xiang R.L. (2020). Effect of Alectinib versus Crizotinib on Progression-Free Survival, Central Nervous System Efficacy and Adverse Events in ALK-Positive Non-Small Cell Lung Cancer: A Systematic Review and Meta-Analysis. Ann. Palliat. Med..

[181] Samacá-Samacá D., Prieto-Pinto L., Peréz A.Y., Valderrama C., Hernández F. (2023). Alectinib for Treating Patients with Metastatic ALK-Positive NSCLC: Systematic Review and Network Metanalysis. Lung Cancer Manag..

[182] Xing P., Hao X., Zhang X., Li J. (2022). Efficacy and Safety of Brigatinib in ALK-Positive Non-Small Cell Lung Cancer Treatment: A Systematic Review and Meta-Analysis. Front. Oncol..

[183] Golding B., Luu A., Jones R., Viloria-Petit A.M. (2018). The Function and Therapeutic Targeting of Anaplastic Lymphoma Kinase (ALK) in Non-Small Cell Lung Cancer (NSCLC). Mol. Cancer.

[184] Horn L., Wang Z., Wu G., Poddubskaya E., Mok T., Reck M., Wakelee H., Chiappori A.A., Lee D.H., Breder V. (2021). Ensartinib vs Crizotinib for Patients with Anaplastic Lymphoma Kinase-Positive Non-Small Cell Lung Cancer: A Randomized Clinical Trial. JAMA Oncol..

[185] Zou H.Y., Friboulet L., Kodack D.P., Engstrom L.D., Li Q., West M., Tang R.W., Wang H., Tsaparikos K., Wang J. (2015). PF-06463922, an ALK/ROS1 Inhibitor, Overcomes Resistance to First and Second Generation ALK Inhibitors in Preclinical Models. Cancer Cell.

[186] Shaw A.T., Solomon B.J., Besse B., Bauer T.M., Lin C.C., Soo R.A., Riely G.J., Ignatius Ou S.H., Clancy J.S., Li S. (2019). ALK Resistance Mutations and Efficacy of Lorlatinib in Advanced Anaplastic Lymphoma Kinase-Positive Non–Small-Cell Lung Cancer. J. Clin. Oncol..

[187] Shaw A.T., Bauer T.M., de Marinis F., Felip E., Goto Y., Liu G., Mazieres J., Kim D.-W., Mok T., Polli A. (2020). First-Line Lorlatinib or Crizotinib in Advanced ALK-Positive Lung Cancer. N. Engl. J. Med..

[188] Ou S.H., Kilvert H., Candlish J., Lee B., Polli A., Thomaidou D., Le H. (2024). Systematic Review and Network Meta-Analysis of Lorlatinib with Comparison to Other Anaplastic Lymphoma Kinase (ALK) Tyrosine Kinase Inhibitors (TKIs) as First-Line Treatment for ALK-Positive Advanced Non-Smallcell Lung Cancer (NSCLC). Lung Cancer.

[189] Cooper A.J., Sequist L.V., Lin J.J. (2022). Third-Generation EGFR and ALK Inhibitors: Mechanisms of Resistance and Management. Nat. Rev. Clin. Oncol..

[190] Lin J.J., Horan J.C., Tangpeerachaikul A., Swalduz A., Valdivia A., Johnson M.L., Besse B., Camidge D.R., Fujino T., Yoda S. (2024). NVL-655 Is a Selective and Brain-Penetrant Inhibitor of Diverse ALK-Mutant Oncoproteins, Including Lorlatinib-Resistant Compound Mutations. Cancer Discov..

[191] Murray B.W., Zhai D., Deng W., Zhang X., Ung J., Nguyen V., Zhang H., Barrera M., Parra A., Cowell J. (2021). TPX-0131, a Potent CNS-Penetrant, Next-Generation Inhibitor of Wild-Type ALK and ALK-Resistant Mutations. Mol. Cancer Ther..

[192] Nuvalent Presents Preliminary Data for Neladalkib in Advanced ALK-Positive Solid Tumors Beyond NSCLC at ESMO 2025. https://www.prnewswire.com/news-releases/nuvalent-presents-preliminary-data-for-neladalkib-in-advanced-alk-positive-solid-tumors-beyond-nsclc-at-esmo-2025-302588111.html.

[193] Chaft J.E., Dagogo-Jack I., Santini F.C., Eng J., Yeap B.Y., Izar B., Chin E., Jones D.R., Kris M.G., Shaw A.T. (2018). Clinical Outcomes of Patients with Resected, Early-Stage ALK-Positive Lung Cancer. Lung Cancer.

[194] Blackhall F.H., Peters S., Bubendorf L., Dafni U., Kerr K.M., Hager H., Soltermann A., O’Byrne K.J., Dooms C., Sejda A. (2014). Prevalence and Clinical Outcomes for Patients with ALK-Positive Resected Stage I to III Adenocarcinoma: Results from the European Thoracic Oncology Platform Lungscape Project. J. Clin. Oncol..

[195] Liu Y., Ye X., Yu Y., Lu S. (2019). Prognostic Significance of Anaplastic Lymphoma Kinase Rearrangement in Patients with Completely Resected Lung Adenocarcinoma. J. Thorac. Dis..

[196] Wang B., Song Y., Chen Z., Su X., Yang X., Wei Z., Chen J., Chen C., Li M. (2023). A Retrospective Study of Postoperative Targeted Therapy in ALK-Positive Lung Cancer. Sci. Rep..

[197] Study Details|NCT03456076|A Study Comparing Adjuvant Alectinib Versus Adjuvant Platinum-Based Chemotherapy in Patients with ALK Positive Non-Small Cell Lung Cancer|ClinicalTrials.Gov. NCT03456076.

[198] Study Details|NCT05341583|Ensartinib as Adjuvant Treatment in Anaplastic Lymphoma Kinase (ALK) Positive Non-Small Cell Lung Cancer|ClinicalTrials.Gov. NCT05341583.

[199] ESMO Congress 2025|OncologyPRO. https://oncologypro.esmo.org/congress-resources/esmo-congress-2025?presentation=ensartinib_as_adjuvant_therapy_in_patients__pts__w.

[200] Adjuvant ALK TKIs Show Robust and Durable DFS Benefit in NSCLC. https://dailyreporter.esmo.org/esmo-congress-2025/non-small-cell-lung-cancer/adjuvant-alk-tkis-show-robust-and-durable-disease-free-survival-benefit-in-non-small-cell-lung-cancer.

[201] Chang Z.H., Zhu T.F., Ou W., Jiang H., Wang S.Y. (2024). A Real-World Retrospective Study to Assess Efficacy and Safety of Alectinib as Adjuvant Therapy in IB-IIIB NSCLC Patients Harboring ALK Rearrangement. Front. Oncol..

[202] Study Details|NCT02194738|Genetic Testing in Screening Patients with Stage IB-IIIA Non-Small Cell Lung Cancer That Has Been or Will Be Removed by Surgery (The ALCHEMIST Screening Trial)|ClinicalTrials.Gov. NCT02194738.

[203] Gerber D.E., Wang Y., Langer C.J., Khullar O.V., Kozono D.E., Cornelius L.A., Forde P.M., Nwaneri M.O., Neal J.W., Talsania A. ECOG-ACRIN E4512: Phase 3 Trial of Crizotinib vs Observation for Surgically Resected Early-Stage ALK+ Non-Small Cell Lung Cancer. Proceedings of the Presented at World Conference on Lung Cancer 2025.

[204] Induction ALK-Tkis for Stage III Non-Small Cell Lung Cancer Harboring ALK Fusion: A Single-Center Experience with 3-Year Follow-Up|The American Association for Thoracic Surgery|AATS. https://www.aats.org/resources/induction-alk-tk-is-for-stage-iii-non-small-cell-lung-cancer-harboring-alk-fusion-a-single-center-experience-with-3-year-follow-up.

[205] Study Details|NCT03088930|Evaluating Crizotinib in the Neoadjuvant Setting in Patients with Non-Small Cell Lung Cancer|ClinicalTrials.Gov. NCT03088930.

[206] Zenke Y., Yoh K., Sakakibara-Konishi J., Daga H., Hosomi Y., Nogami N., Okamoto I., Matsumoto S., Kuroda S., Wakabayashi M. (2019). P1.18-04 Neoadjuvant Ceritinib for Locally Advanced Non-Small Cell Lung Cancer with ALK Rearrangement: SAKULA Trial. J. Thorac. Oncol..

[207] Record History|Ver. 2: 2023-05-20|NCT05740943|ClinicalTrials.Gov. NCT05740943.

[208] Study Details|NCT05361564|A Window of Opportunity Study for Investigating Drug Tolerant Persister (DTP) to Preoperative Brigatinib in Resectable Non-Small Cell Lung Cancer (NSCLC) Harboring ALK Fusions|ClinicalTrials.Gov. NCT05361564.

[209] Study Details|NCT05765877|Neoadjuvant WX-0593 in Resectable ALK-Positive or ROS1-Positive Non-Small Cell Lung Cancer|ClinicalTrials.Gov. NCT05765877.

[210] Zhang G., Liu Y., Shen D., Chen Y., He Y., Wang H., Wang L., Zhang H., Zhang H., Zhao J. (2025). Neoadjuvant and Adjuvant Iruplinalkib in Resectable ALK or ROS1 Fusion-Positive, Non-Small Cell Lung Cancer (NSCLC): The Preliminary Results of the Single-Arm, Exploratory Neo-INFINITY Study. J. Clin. Oncol..

[211] Study Details|NCT05015010|Alectinib in Neo-Adjuvant Treatment of Stage III NSCLC|ClinicalTrials.Gov. NCT05015010.

[212] Study Details|NCT04302025|A Study of Multiple Therapies in Biomarker-Selected Participants with Resectable Stages IB-III Non-Small Cell Lung Cancer (NSCLC)|ClinicalTrials.Gov. NCT04302025.

[213] Study Details|NCT06563999|Neoadjuvant Umbrella Trial for Patients with Unresectable Stage III NSCLC Harboring Rare Mutations.|ClinicalTrials.Gov. NCT06563999.

[214] Friedlaender A., Perol M., Banna G.L., Parikh K., Addeo A., Ch A.A. (2024). REVIEW Open Access Oncogenic Alterations in Advanced NSCLC: A Molecular Super-Highway. Biomark. Res..

[215] Lim T.K.H., Skoulidis F., Kerr K.M., Ahn M.J., Kapp J.R., Soares F.A., Yatabe Y. (2023). KRAS G12C in Advanced NSCLC: Prevalence, Co-Mutations, and Testing. Lung Cancer.

[216] Lawrence R.E., Salgia R. (2010). MET Molecular Mechanisms and Therapies in Lung Cancer. Cell Adh. Migr..

[217] Odintsov I., Makarem M., Nishino M., Bachert S.E., Zhang T., LoPiccolo J., Paweletz C.P., Gokhale P.C., Ivanova E., Saldanha A. (2024). Prevalence and Therapeutic Targeting of High-Level ERBB2 Amplification in NSCLC. J. Thorac. Oncol..

[218] Pillai R.N., Behera M., Berry L.D., Rossi M.R., Kris M.G., Johnson B.E., Bunn P.A., Ramalingam S.S., Khuri F.R. (2017). HER2 Mutations in Lung Adenocarcinomas: A Report from the Lung Cancer Mutation Consortium. Cancer.

[219] Gainor J.F., Shaw A.T. (2013). Novel Targets in Non-Small Cell Lung Cancer: ROS1 and RET Fusions. Oncologist.

[220] Davies K.D., Doebele R.C. (2013). Molecular Pathways: ROS1 Fusion Proteins in Cancer. Clin. Cancer Res..

[221] Westphalen C.B., Krebs M.G., Le Tourneau C., Sokol E.S., Maund S.L., Wilson T.R., Jin D.X., Newberg J.Y., Fabrizio D., Veronese L. (2021). Genomic Context of NTRK1/2/3 Fusion-Positive Tumours from a Large Real-World Population. Npj Precis. Oncol..

[222] Michels S., Scheel A.H., Scheffler M., Schultheis A.M., Gautschi O., Aebersold F., Diebold J., Pall G., Rothschild S., Bubendorf L. (2016). Clinicopathological Characteristics of RET Rearranged Lung Cancer in European Patients. J. Thorac. Oncol..

[223] Chen D., Zhang L.Q., Huang J.F., Liu K., Chuai Z.R., Yang Z., Wang Y.X., Shi D.C., Liu Q., Huang Q. (2014). BRAF Mutations in Patients with Non-Small Cell Lung Cancer: A Systematic Review and Meta-Analysis. PLoS ONE.

[224] Study Details|NCT04819100|A Study of Selpercatinib After Surgery or Radiation in Participants with Non-Small Cell Lung Cancer (NSCLC)|ClinicalTrials.Gov. NCT04819100.

[225] Study Details|NCT03157128 | A Study of Selpercatinib (LOXO-292) in Participants with Advanced Solid Tumors, RET Fusion-Positive Solid Tumors, and Medullary Thyroid Cancer (LIBRETTO-001)|ClinicalTrials.Gov. NCT03157128.

[226] Rajaram R., Sholl L.M., Dacic S., Goldman J.W., Tan D.S.-W., Gautschi O., Loong H.H.F., De Braud F.G., Massarelli E., Levy B.P. (2022). LIBRETTO-001 Cohort 7: A Single-Arm, Phase 2 Study of Neoadjuvant Selpercatinib in Patients with Resectable Stage IB-IIIA RET Fusion-Positive NSCLC. J. Clin. Oncol..

[227] Canon J., Rex K., Saiki A.Y., Mohr C., Cooke K., Bagal D., Gaida K., Holt T., Knutson C.G., Koppada N. (2019). The Clinical KRAS(G12C) Inhibitor AMG 510 Drives Anti-Tumour Immunity. Nature.

[228] Study Details|NCT06890598|Study of Olomorasib (LY3537982) in Combination with Standard of Care in Participants with Resected or Unresectable KRAS G12C-Mutant Non-Small Cell Lung Cancer|ClinicalTrials.Gov. NCT06890598.

[229] Study Details|NCT05400577|Sotorasib in KRAS G12C Mutated, Resectable, Stage Ib-IIIA NSCLC|ClinicalTrials.Gov. NCT05400577.

[230] Study Details|NCT05472623|Neoadjuvant KRAS G12C Directed Therapy with Adagrasib (MRTX849) with or Without Nivolumab|ClinicalTrials.Gov. NCT05472623.

[231] Liu Y.T., Hao X.Z., Liu D.R., Cheng G., Zhang S.C., Xiao W.H., Hu Y., Liu J.F., He M., Ding C.M. (2020). Icotinib as Adjuvant Treatment for Stage II-IIIA Lung Adenocarcinoma Patients with Egfr Mutation (ICWIP Study): Study Protocol for a Randomised Controlled Trial. Cancer Manag. Res..

[232] Study Details|NCT04762459|Efficacy and Safety of Almonertinib Combined with or Without Chemotherapy as an Adjuvant Treatment for Stage II-IIIA Non-Small Cell Lung Carcinoma Following Complete Tumour Resection|ClinicalTrials.Gov. NCT04762459.

[233] To Assess the Efficacy and Safety of Furmonertinib Versus Placebo, in Patients with Epidermal Growth Factor Receptor Mutation Positive Stage II-IIIA Non-Small Cell Lung Carcinoma, Following Complete Tumour Resection with or Without Adjuvant Chemotherapy. https://trialfinder.panfoundation.org/en-US/trial/listing/282490.

[234] Study Details|NCT06041776|Adjuvant Befotertinib in Stage IB-IIIB Non-Small Cell Lung Cancer with Positive EGFR Sensitive Mutations|ClinicalTrials.Gov. NCT06041776.

[235] Study Details|NCT06955325|Umbrella Trial of Adjuvant Therapy in Completely Resected High-Risk Stage IA-IB NSCLC: Focus on Driver Mutations|ClinicalTrials.Gov. NCT06955325.

[236] Study Details|NCT06784791|Preoperative Amivantamab or Amivantamab and Carboplatin/Pemetrexed Treatment in Patients with Resectable Non-Small-Cell Lung Cancer Harboring Oncogenic EGFR Mutations (NEOpredict-EGFR)|ClinicalTrials.Gov. NCT06784791.

[237] Study Details|NCT05011487|Neoadjuvant Osimertinib + Chemotherapy for EGFR-Mutant Stage III NSCLC|ClinicalTrials.Gov. NCT05011487.

[238] Study Details|NCT05104788|A Study of Icotinib with Chemotherapy as Neoadjuvant Therapy for Patients with EGFRm Positive Resectable Non-Small Cell Lung Cancer|ClinicalTrials.Gov. NCT05104788.

[239] Study Details|NCT01470716|Neoadjuvant Erlotinib for Operable Stage II or IIIA NSCLC with EGFR Mutations|ClinicalTrials.Gov. NCT01470716.

[240] Study of Furmonertinib as Neoadjuvant Therapy for Resectable Stage B EGFR Sensitive Mutant NSCLC (NCT05987826)|CareAcross Lung Cancer Service. NCT05987826.

[241] Study Details|NCT05430802|Neoadjuvant Furmonertinib and Cisplatin/Pemetrexed as in EGFR Mutated Stage IIIA-IIIB Resectable NSCLC (FORESEE)|ClinicalTrials.Gov. NCT05430802.

[242] Study Details|NCT04455594|Almonertinib Vs. Erlotinib/Chemotherapy for Neo-AdjuVant Treatment of Stage IIIA-N2 EGFR-Mutated NSCLC|ClinicalTrials.Gov. NCT04455594.

[243] Study Details|NCT03203590|Clinical Trial of Neoadjuvant Targeted Treatment to NSCLC Patients|ClinicalTrials.Gov. NCT03203590.

[244] Study Details|NCT06268210|Neoadjuvant Lazertinib with or Without Chemotherapy for Patients with EGFR-Mutated Resectable Non-Small Cell Lung Cancer|ClinicalTrials.Gov. NCT06268210.

[245] Study Details|NCT04470076|Neoadjuvant Afatinib Combination with Chemotherapy for Stage Ⅱa-Ⅲb NSCLC with EGFR Activating Mutation|ClinicalTrials.Gov. NCT04470076.

[246] Study Details|NCT03349203|Icotinib as Neoadjuvant and Adjuvant Therapy in EGFR-Mutant Stage IIIB or Oligometastasis Non-Small Cell Lung Cancer|ClinicalTrials.Gov. NCT03349203.

[247] Study Details|NCT05469022|Neoadjuvant Lazertinib Therapy in EGFR-Mutation Positive Lung Adenocarcinoma Detected by BALF Liquid Biopsy|ClinicalTrials.Gov. NCT05469022.

[248] Study Details|NCT03749213|Icotinib as Neoadjuvant Therapy in EGFR-Mutant Stage ⅢA-N2 Non-Small Cell Lung Cancer|ClinicalTrials.Gov. NCT03749213.

[249] Study Details|NCT05132985|Neoadjuvant Icotinib with Chemotherapy for Epidermal Growth Factor Receptor(EGFR)-Mutated Resectable Lung Adenocarcinoma|ClinicalTrials.Gov. NCT05132985.

[250] Study Details|NCT06194448|To Evaluate the Efficacy/Safety of Osimertinib Prior to CRT and Maintenance of It with Stage III, Unresectable NSCLC with EGFR Mutations|ClinicalTrials.Gov. NCT06194448.

[251] Study Details|NCT05241028|Adjuvant Therapy of Ensartinib in Stage IB-IIIA ALK-Positive Non-Small Cell Lung Cancer|ClinicalTrials.Gov. NCT05241028.

[252] Study Details|NCT05186506|A Study Comparing Ensatinib Versus Platinum-Based Chemotherapy as Adjuvant Treatment for Stage II-IIIA ALK -Positive Non-Small Cell Lung Cancer|ClinicalTrials.Gov. NCT05186506.

[253] Minimal Residual Disease Guiding Adjuvant Therapy in Stage I NSCLC. https://ctv.veeva.com/study/minimal-residual-disease-guiding-adjuvant-therapy-in-stage-i-nsclc.

[254] Study Details|NCT06780839|Adjuvant Treatment of ALK-Positive Non-Small Cell Lung Cancer with Ensartinib Guided by MRD|ClinicalTrials.Gov. NCT06780839.

[255] Study Details|NCT06772610|The Efficacy and Safety of Ensartinib As Adjuvant Therapy in Stage I ALK-Positive NSCLC Patients with High Risk Factors|ClinicalTrials.Gov. NCT06772610.

[256] Study Details|NCT02201992|Crizotinib in Treating Patients with Stage IB-IIIA Non-Small Cell Lung Cancer That Has Been Removed by Surgery and ALK Fusion Mutations (An ALCHEMIST Treatment Trial)|ClinicalTrials.Gov. NCT02201992.

[257] Study Details|NCT06862869|Real World Clinical Outcomes of Resected ALK-Positive Early Stage NSCLC Patients Treated with Alectinib as Adjuvant Therapy|ClinicalTrials.Gov. NCT06862869.

[258] Study Details|NCT06624059|A Study to See How Well and How Safely Different Treatments Work in a Group of Participants with Non-Small Cell Lung Cancer (NSCLC)|ClinicalTrials.Gov. NCT06624059.

[259] Study Details|NCT05380024|A Study of Ensartinib as Neoadjuvant Therapy for Patients with ALK Positive Resectable Non-Small Cell Lung Cancer|ClinicalTrials.Gov. NCT05380024.

[260] Study Details|NCT06779539|Neoadjuvant Ensartinib in ALK Positive Resectable Stage II to III Non-Small Cell Lung Cancer|ClinicalTrials.Gov. NCT06779539.

[261] Study Details|NCT06682884|Lorlatinib as Neoadjuvant Treatment in Stage IB-IIIB ALK-Rearranged Non-Small Cell Lung Cancer|ClinicalTrials.Gov. NCT06682884.

[262] Study Details|NCT06785584|Efficacy and Safety of Ensartinib in Neoadjuvant Therapy for Stage IIA—IIIB (Operable or Potentially Operable) ALK-Positive Lung Adenocarcinoma: A Multicenter, Real-World Clinical Study|ClinicalTrials.Gov. NCT06785584.

[263] Study Details|NCT06736561|A Multicenter Real-World Clinical Study on the Efficacy and Safety of Ensartinib As Neoadjuvant Treatment for Anaplastic Lymphoma Kinase (ALK) Positive Non-Small Cell Lung Cancer (NSCLC) Patients|ClinicalTrials.Gov. NCT06736561.

[264] Study Details|NCT06282536|Neoadjuvant Therapy with Iruplinalkib for Potentially Resectable ALK Positive NSCLC: A Single Arm, Exploratory Trial|ClinicalTrials.Gov. NCT06282536.

[265] Study Details|NCT05118854|A Phase II Study of Neoadjuvant Sotorasib in Combination with Cisplatin or Carboplatin and Pemetrexed for Surgically Resectable Stage IIA-IIIB Non-Squamous Non-Small Cell Lung Cancer with a KRAS p.G12C Mutation|ClinicalTrials.Gov. NCT05118854.

[266] Study Details|NCT05714891|Neoadjuvant Platform Trial in Non-Small Cell Lung Cancer|ClinicalTrials.Gov. NCT05714891.

[267] Study Details|NCT07156604|Vebreltinib for Neoadjuvant in METex 14 Skipping Mutant Stage IIA-IIIB (N2) Non-Small Cell Lung Cancer (NSCLC)|ClinicalTrials.Gov. NCT07156604.

[268] Study Details|NCT05800340|Neoadjuvant Immunotherapy in Rare Mutations Localized NSCLC|ClinicalTrials.Gov. NCT05800340.

[269] Study Details|NCT06644313|Vebreltinib for Neoadjuvant in MET-Altered Stage IIIA-IIIB (N2) Non-Small Cell Lung Cancer (NSCLC)|ClinicalTrials.Gov. NCT06644313.

[270] Study Details|NCT04926831|Phase II of Neoadjuvant and Adjuvant Capmatinib in NSCLC|ClinicalTrials.Gov. NCT04926831.

[271] Study Details|NCT06054191|Neoadjuvant and Adjuvant Targeted Treatment in NSCLC with BRAF V600 or MET Exon 14 Mutations|ClinicalTrials.Gov. NCT06054191.

[272] Study Details|NCT07195695|Beamion LUNG-3: A Study to Test Whether Zongertinib Helps People with Surgically Removed, Non-Small Cell Lung Cancer with HER2 Mutations Compared with Standard Treatment|ClinicalTrials.Gov. NCT07195695.

[273] Study Details|NCT06734182|Neoadjuvant Envafolimab Plus Disitamab Vedotin and Carboplatin in Resectable HER2-Mutant Non-Small-Cell Lung Cancer|ClinicalTrials.Gov. NCT06734182.

