# Supplementary material for: Neoadjuvant, Adjuvant and Perioperative Treatment in Early-Stage Non-Small Cell Lung Cancer (NSCLC) with Actionable Genomic Alterations: Current Landscape and Future Perspectives
Source: Cancers (Basel). 2026 Feb 2;18(3):493. doi: 10.3390/cancers18030493 (PMC12897308; doi:10.3390/cancers18030493)
Supplement: Supplementary file 1 [file cancers-18-00493-s001.zip › cancers-4087944-supplementary.pdf]

Supplementary Table S1. Major Phase 2 and 3 trials of neoadjuvant, adjuvant and perioperative use of immunotherapy (checkpoint inhibitors) in early resectable NSCLC

Study, Phase, Stage	Strategy, Study Arms	EFS/DFS/PFS (months or %)	OS (months or %)	pCR (%)	MPR (%)
CheckMate816, 3, IB-IIIA resectable [27,51]	Neoadjuvant, **3 cy Nivolumab + ChT** vs 3 cy Nivolumab + 1 cy Ipilimumab vs ChT	mEFS, **N vs C: 43.8 vs 18.4, HR: 0.66 (CI: 0.49–0.90),** I/N vs C: 54.8 vs 20.9, HR: 0.77 (CI: 0.51–1.15)	**5-year OS, N vs C: 65.4% vs 55%, HR: 0.72 (CI: 0.52-0.99),** 3-year OS I/N vs C: 73% vs 61%, HR: 0.73 (CI: 0.47-1.14)	**N vs C: 24 vs 2.2, OR 13.94 (CI: 3.49–55.75), I/N vs C: 20.4 vs 4.6, OR 5.14 (CI: 1.91-13.8)**	**N vs C: 36.9 vs 8.9, OR 5.70 (CI: 3.16–10.26), I/N vs C: 28.3 vs 14.8, OR 2.15 (CI: 1.13-4.1)**
NEOSTAR, 2, IB-IIIA, operable [52,53]	Neoadjuvant, 3 cy Nivolumab vs 3 cy Nivolumab + 1 cy Ipilimumab vs 3 cy Nivolumab + ChT vs 3 cy Nivolumab + 1 cy Ipilimumab vs ChT	NR	NR	N 2 (CI: 1-28) vs I/N 6 (11-52) vs N + C 18.2 (CI: 5.2–40.3) vs I/N + C 18.2 (CI: 5.2–40.3)	N 22 (CI: 7–44) vs I/N 38 (CI: 18–62) vs N + C 32.1 (80% CI: 18.7-43.1) vs I/N + C 50 (80% CI: 34.6-61.1)
PEARLS/KEY-NOTE-091, 3, IB-IIIA [50]	Adjuvant, 18 cy Pembrolizumab vs placebo (ChT optional)	mDFS, **P vs C, ITT: 53.6 vs 42, HR: 0.76 (CI: 0.63–0.91)**, PD-L1 ≥ 50%: NR vs NR, HR: 0.82 (CI: 0.57–1.18)	Not reported	NA	NA
IMpower010, 3, IB-IIIA [49]	Adjuvant, 16 cy Atezolizumab vs BSC after ChT	mDFS; II-IIIA, **A vs BSC, PD-L1 ≥ 1%: 68.5 vs 37.3, HR: 0.7 (CI: 0.55, 0.91), PD-L1 ≥ 50%: NR vs 41.1, HR: 0.48 (CI: 0.32–0.72)**, all PD-L1: 57.4 vs 40.8, HR: 0.83 (CI: 0.69–1.00)	mOS; II-IIIA, A vs BSC, PD-L1≥1%: NR vs NR, HR: 0.71 (CI: 0.49, 1.03), **PD-L1≥50%: NR vs NR, HR: 0.43 (CI: 0.24–0.78)**, all PD-L1: NR vs NR, HR: 0.95 (CI: 0.74–1.24)	NA	NA
CCTG BR.31, 3, IB-IIIA [54]	Adjuvant, 12 cy Durvalumab vs placebo (ChT optional)	mDFS, D vs C, PD-L1 ≥ 1%: 60 vs 60, HR: 0.99 (CI: 0.79, 1.25), PD-L1 ≥ 25%: 70 vs 60, HR: 0.94 (CI: 0.71–1.25), all PD-L1: 60 vs 54, HR: 0.89 (CI: 0.75–1.07)	Not reported	NA	NA
NADIM ADJUVANT, 2, IB-IIIA [55]	Adjuvant, 4 cy Nivolumab + ChT followed by 6 cy Nivolumab vs 4 cy ChT	mDFS, N + C vs C: NR vs NR, HR: 0.65 (0.40-1.07)	mOS, N + C vs C: NR vs NR, HR: 0.99 (0.49-2.01)	NA	NA
CheckMate 77T, 3, IIA-IIIB, reesectable [26]	Perioperative, 4 cy Nivolumab + ChT→ 1 year Nivolumab vs 4 cy ChT→placebo	mEFS, **N + C vs C: NR vs 18.4, HR: 0.58 (CI: 0.42–0.81)**	Interim mOS, N + C vs C: NR vs NR, HR: 0.85 (CI: 0.58-1.25)	**N + C vs C: 25.3 vs 4.7, OR: 6.64 (CI: 3.4–12.97)**	**N + C vs C: 35.4 vs 12.1, OR: 4.01 (CI: 2.48–6.49)**

KEYNOTE-671, 3, II-IIIB, resectable [28,30]	Perioperative, 4 cy Pembrolizumab + ChT→ 13 cy Pembrolizumab vs 4 cy ChT→placebo	mEFS, **P + C vs C: 47.2 vs 18.3, HR: 0.59 (CI: 0.48–0.72)**	2nd interim mOS, **P + C vs C: NR vs 52.4, HR: 0.72 (CI: 0.56–0.93)**	**P + C vs C: 18.1 vs 4.0, difference: 14.2 (CI: 10.1–18.7)**	**P + C vs C: 30.2 vs 11, difference: 19.2 (CI: 13.9–24.7)**
AEGEAN, 3, II-IIIB (N2), resectable [35]	Perioperative, 4 cy Durvalumab + ChT→ 12 cy Durvalumab vs 4 cy ChT→placebo	mEFS, **D + C vs C: NR vs 25.9, HR: 0.68 (CI: 0.53–0.88)**	NR	**D + C vs C: 17.2 vs 4.3, difference: 13.0 (CI: 8.7-17.6)**	**D + C vs C: 33.3 vs 12.3, difference: 21.0 (CI: 15.1–26.9)**
NEOTORCH, 3, II-IIIB, resectable [34]	Perioperative, 3 cy Toripalimab + ChT→ 1 cy ChT + 13 cy Toripalimab vs 3 cy ChT→1 cy ChT	**Tor + C vs C,** mEFS: **NR vs 15.1, HR: 0.40 (CI: 0.28–0.57)**/mDFS: **NR vs 22.0, HR: 0.49 (CI: 0.31–0.76**	mOS, Tor + C vs C: NR vs 30.4, HR: 0.62 (CI: 0.38–1.00)	**Tor + C vs C: 24.8 vs 1, difference: 23.7 (CI: 17.6–29.8)**	**Tor + C vs C: 48.5 vs 8.4, difference: 40.2 (CI: 32.2–48.1)**
RATIONALE-315, 3, II-IIIA, resectable [33]	Perioperative, 3-4 cy Tislelizumab + ChT→ 1 year Tislelizumab vs 3-4 cy ChT→placebo	mEFS, **Tis + C vs C: NR vs NR, HR: 0.56 (CI: 0.40–0.79)**	mOS, **Tis + C vs C: NR vs NR, HR: 0.62 (CI: 0.39–0.98)**	**Tis + C vs C: 41 vs 6, difference: 35 (CI: 28–42)**	**Tis + C vs C: 56 vs 15, difference: 41 (CI: 33–95)**
NADIM, 2, IIIA, resectable [48]	Perioperative, 3 cy Nivolumab + ChT→ 1 year Nivolumab	5-year PFS: 65% (CI: 49.4-76.9)	5-year OS: 69.3% (CI: 53.7-80.6)	63.4 (CI: 62–91)	83 (CI: 68–93)
NADIM II, 2, IIIA-IIIB, resectable [56]	Perioperative, 3 cy Nivolumab + ChT→ 6 months Nivolumab vs 3 cy ChT→placebo	24-month PFS, **N + C vs C: 67.2% vs 40.9%, HR: 0.47 (CI: 0.25-0.88)**	24-month OS, **N + C vs C: 85% vs 63.6%, HR: 0.43 (CI: 0.19-0.98)**	**N + C vs C: 37 vs 7, RR: 5.34 (CI: 1.34–21.23)**	**N + C vs C: 53 vs 14, RR: 3.82 (CI: 1.49–9.79)**
IMpower030, 3, II-IIIB, resectable [57]	Perioperative, 4 cy Atezolizumab + ChT→16 cy Atezolizumab vs 4 cy ChT→BSC	Ongoing, not reported	Ongoing, not reported	Ongoing, not reported	Ongoing, not reported

Abbreviations: A→Atezolizumab, BSC→best supportive care, ChT/C→chemotherapy, CI→95% confidence interval (except where stated differently), DFS→disease-free survival, D→Durvalumab, EFS→event-free survival, HR→hazard ratio, I→Ipilimumab, MPR→major pathologic response, NA→not applicable, N→Nivolumab, NR→not reached, OR→odds ratio, OS→overall survival, pCR→pathologic complete response, P→Pembrolizumab, PFS→progression-free survival, RR→relative ratio, Tis→Tislelizumab, Tor→Toripalimab,
